# Argonaute Utilization for miRNA Silencing Is Determined by Phosphorylation-Dependent Recruitment of LIM-Domain-Containing Proteins

**DOI:** 10.1016/j.celrep.2017.06.027

**Published:** 2017-07-05

**Authors:** Katherine S. Bridge, Kunal M. Shah, Yigen Li, Daniel E. Foxler, Sybil C.K. Wong, Duncan C. Miller, Kathryn M. Davidson, John G. Foster, Ruth Rose, Michael R. Hodgkinson, Paulo S. Ribeiro, A. Aziz Aboobaker, Kenta Yashiro, Xiaozhong Wang, Paul R. Graves, Michael J. Plevin, Dimitris Lagos, Tyson V. Sharp

**Affiliations:** 1 Centre for Molecular Oncology, Barts Cancer Institute, Queen Mary University of London, John Vane Science Centre, Charterhouse Square, London EC1M 6BQ, UK; 2 School of Biological and Chemical Sciences, Queen Mary University of London, Fogg Building, Mile End Road, London E1 4NS, UK; 3 Department of Biology, University of York, Heslington, York YO10 5DD, UK; 4 Centre for Tumour Biology, Barts Cancer Institute, Queen Mary University of London, John Vane Science Centre, Charterhouse Square, London EC1M 6BQ, UK; 5 Department of Zoology, University of Oxford, The Tinbergen Building, South Parks Road, Oxford OX1 3PS, UK; 6 Department of Radiation Oncology, New York-Presbyterian Brooklyn Methodist Hospital, 506 6th Street, Brooklyn, NY 11215, USA; 7 Department of Biochemistry, Molecular Biology and Cell Biology, Northwestern University, Evanston, IL 60208, USA; 8 Centre for Immunology and Infection, Hull York Medical School and Department of Biology, University of York, Heslington, York YO10 5DD, UK

**Keywords:** microRNA, Argonaute, AGO2, miRISC, LIMD1, WTIP, TNRC6A, GW182, DDX6, AKT

## Abstract

As core components of the microRNA-induced silencing complex (miRISC), Argonaute (AGO) proteins interact with TNRC6 proteins, recruiting other effectors of translational repression/mRNA destabilization. Here, we show that LIMD1 coordinates the assembly of an AGO-TNRC6 containing miRISC complex by binding both proteins simultaneously at distinct interfaces. Phosphorylation of AGO2 at Ser 387 by Akt3 induces LIMD1 binding, which in turn enables AGO2 to interact with TNRC6A and downstream effector DDX6. Conservation of this serine in AGO1 and 4 indicates this mechanism may be a fundamental requirement for AGO function and miRISC assembly. Upon CRISPR-Cas9-mediated knockout of LIMD1, AGO2 miRNA-silencing function is lost and miRNA silencing becomes dependent on a complex formed by AGO3 and the LIMD1 family member WTIP. The switch to AGO3 utilization occurs due to the presence of a glutamic acid residue (E390) on the interaction interface, which allows AGO3 to bind to LIMD1, AJUBA, and WTIP irrespective of Akt signaling.

## Highlights

• LIMD1 is necessary for the Akt3-dependent assembly of an AGO-TNRC6A functional miRISC
• AGO1, 2, and 4 recruit LIMD1 in a phospho-dependent manner
• AGO2 to AGO3 switching occurs upon LIMD1 ablation in HeLa cells
• AGO3-WTIP replaces AGO2-LIMD1 for miRNA silencing in an Akt3-independent manner

## Graphical Abstract

**Figure:**
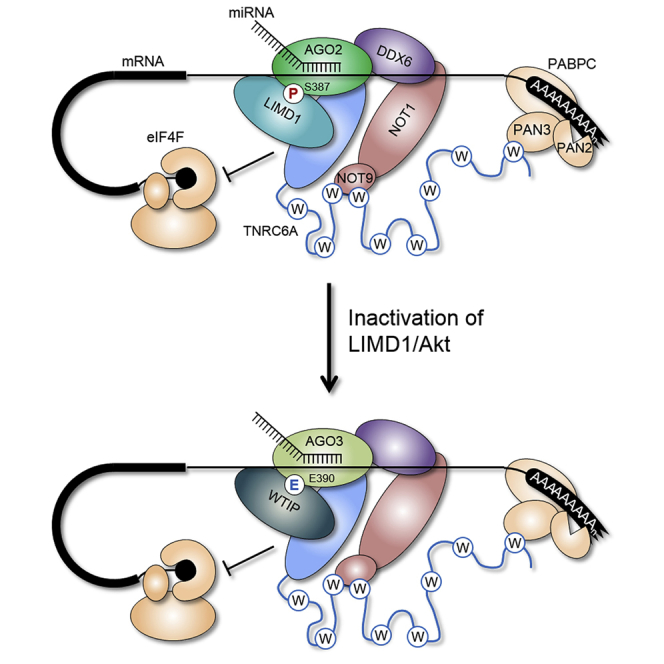

## Teaser

Argonaute (AGO) proteins mediate post-transcriptional gene silencing through formation of the microRNA-induced silencing complex (miRISC). Bridge et al. identify LIM-domain-containing proteins as essential for miRISC formation through a phosphorylation-dependent mechanism. This is critical for post-transcriptional gene silencing and reveals that miRISC functionality is maintained by “AGO switching.”

## Introduction

MicroRNAs are ∼22 nucleotide non-coding RNA molecules that silence gene expression post-transcriptionally (Bartel, 2004). Their loading onto Argonaute (AGO) proteins facilitates base-pairing to mRNA targets with partial complementarity and ultimately translational repression and mRNA degradation (Huntzinger and Izaurralde, 2011, Hutvagner and Simard, 2008). Repression is executed by the microRNA-induced silencing complex (miRISC), a large multi-subunit complex assembled by AGO-mediated recruitment of GW182/TNRC6 proteins and downstream effector complexes (Behm-Ansmant et al., 2006, Chekulaeva et al., 2011, Lian et al., 2009, Liu et al., 2005). TNRC6 proteins facilitate translational repression, mRNA destabilization, and ultimately degradation of target mRNAs via recruitment of effector proteins to the miRISC, such as those involved in decapping (DCP1/2), RNA unwinding (DDX6), and deadenylation (CCR4-NOT) (Bazzini et al., 2012, Chen et al., 2014, Chu and Rana, 2006, Rouya et al., 2014).

Despite significant advances in our understanding of the terminal effects of miRNAs on their targets, the early steps of miRISC assembly and its functional activation remain poorly understood. Recent studies have explored the role of post-translational modification of AGO proteins (Jee and Lai, 2014, Rüdel et al., 2011). For example, phosphorylation of AGO2 Y529 inhibits loading of small RNAs (Rüdel et al., 2011), while EGFR-dependent phosphorylation of AGO2 Y393 prevents processing of looped precursor RNAs into mature miRNAs (Shen et al., 2013). Additionally, Akt3-mediated phosphorylation of AGO2 S387 reduces the mRNA cleavage activity of AGO2 and drives it toward translational repression associated with miRNA-mediated silencing (Horman et al., 2013, Zeng et al., 2008). It has also recently been shown that primary T cells display a signaling-dependent shift in miRISC configuration. While the majority of miRNAs were found in low-molecular-weight miRISC complexes in resting T cells, stimulation of phosphatidylinositol 3-kinase (PI3K) signaling caused an increased association of miRNAs with high-molecular-weight miRISC complexes and an enhancement of miRNA-mediated silencing (La Rocca et al., 2015). These data indicate that miRISC assembly into an active complex and the protein-protein interactions therein are highly dynamic processes, controlled by signal transduction cascades.

To date, the four human AGO proteins (AGO1–4) have been demonstrated to function largely redundantly with regards to miRNA loading and target recognition, with the functionally dominant AGO currently attributed to the expression levels of these proteins (Dueck et al., 2012, Su et al., 2009, Wang et al., 2012). How these overlapping functions are facilitated in light of recent findings demonstrating the role of signaling pathways in the regulation of miRNA biogenesis and function is unclear. Interestingly, plasticity in AGO usage following genetic ablation has been observed (Wang et al., 2012), although the mechanism underpinning this switch remains unknown.

The discovery that AGO2 activity is regulated by phosphorylation, and indeed other post-translational modifications, raises the question as to whether all four human AGO proteins are subject to regulation by signaling, and whether, through differential signal transduction and phosphorylation, may not exhibit complete redundancy (Golden et al., 2017, Lopez-Orozco et al., 2015, Patranabis and Bhattacharyya, 2016, Sahin et al., 2014, Shen et al., 2013, Zeng et al., 2008). Horman and colleagues suggest that the AGO2 S387 phosphorylation diverts its activity from mRNA cleavage to (or toward) miRNA-mediated silencing (Horman et al., 2013). However, this study did not consider the high homology within the S387-containing L2 domain between AGO2 and other human AGO proteins, which are not involved in small interfering RNA (siRNA) silencing. We therefore sought to determine whether signaling regulates miRISC assembly and function across the entire AGO family and whether this regulation would identify an additional dimension of AGO family member functional specificity.

LIMD1 is a component of miRISC required for silencing (James et al., 2010). However, its precise function in miRISC and miRNA silencing is poorly understood. Here, we demonstrate that LIMD1 is crucial for AGO2 miRNA function and that loss of AGO2-LIMD1-mediated miRNA silencing reveals a previously unknown mechanism of AGO selection, with LIMD1 association being a key determinant of AGO utilization. LIMD1 is required for the recruitment of TNRC6A and downstream effectors (DDX6) to AGO2. We show that Akt3-mediated phosphorylation of AGO2 (S387) promotes a phospho-dependent interaction with LIMD1 that is vital for enabling engagement of AGO2 with TNRC6A, revealing the mechanism by which AGO-S387 phosphorylation regulates miRISC function. The phosphorylation-dependent interaction with LIMD1 also extends to the other human AGO family members, with a conserved serine also present in AGO1 and AGO4. AGO3 lacks this conserved serine residue but instead contains a phospho-mimic glutamic acid residue (E390), which facilitates signaling-independent binding to LIMD1 family members. Our findings demonstrate that loss of LIMD1 abolishes AGO2-mediated miRNA silencing and results in redistribution of AGO utilization for miRNA silencing to AGO3.

## Results

### AGO Utilization Is Determined by LIMD1

**Figure 1:**
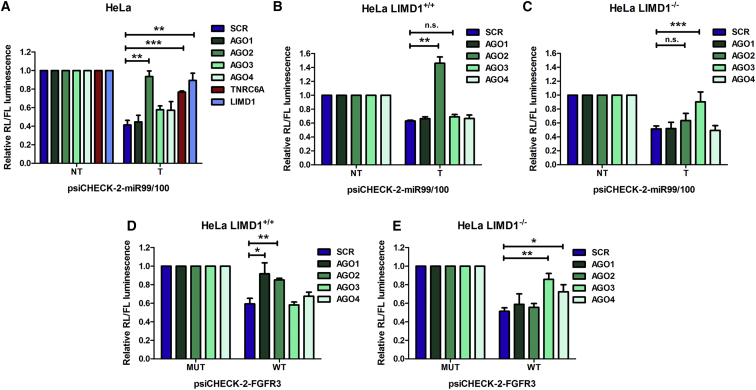
Genetic Ablation of LIMD1 Switches Dependency upon Argonaute Species for miRNA Silencing (A) psiCHECK-2-miR-99/100 luciferase reporter (NT, non-targeting reporter; T, targeting reporter; mean ± SEM; n = 4 treated with indicated siRNAs (SCR, non-targeting control). (B and C) (B) psiCHECK-2-miR99/100 reporter in CRISPR-Cas9 gene-edited HeLa cells with Cas9 alone control (LIMD1^+/+^) or (C) LIMD1 knockout (LIMD1^−/−^) cells, treated with the indicated siRNAs. (D and E) (D) psiCHECK-2-FGFR3 (endogenous mutant [MUT] or wild-type [WT] 3′ UTR) reporter in CRISPR-Cas9 gene-edited HeLa cells with Cas9 alone control (LIMD1^+/+^) or (E) LIMD1 knockout (LIMD1^−/−^) cells, treated with the indicated siRNAs.

Members of the LIMD1 family (LIMD1, AJUBA, and WTIP) of LIM-domain-containing proteins associate with miRISC and are required for miRNA-mediated silencing (James et al., 2010). To examine the role of LIMD1 on AGO2 function, we designed a Renilla luciferase reporter containing five targeted or non-targeted miR-99/100 binding sites in its 3′ UTR. We observed significant de-repression of the targeted reporter upon AGO2, TNRC6A, or LIMD1 siRNA-mediated knockdown in HeLa cells, whereas little or no de-repression was seen with depletion of AGO1, 3, or 4 (Figure 1A and S1A). The high specificity of this reporter for AGO2 is surprising as a previous study did not find evidence for miRNA sorting into AGO1, 2, or 3 in HeLa cells and concluded that association of a particular miRNA with a particular AGO solely reflected AGO protein abundance (Dueck et al., 2012). Moreover, mRNA levels of the targeted reporter were unchanged, indicating that, at the time points analyzed, the reporter is subject to miRNA-mediated translation inhibition rather than target cleavage (Figure S1B).

To investigate the mechanistic function of LIMD1 in miRNA silencing independently of engagement of the RNAi machinery, we genetically ablated *LIMD1* in HeLa cells using CRISPR-Cas9 technology (Figure S1C). Unexpectedly, we found that repression of the miR-99/100 reporter was equivalent in *LIMD1*^*+/+*^ and *LIMD1*^*−/−*^ cell lines (∼50% repression of the targeted reporter), although expression of exogenous LIMD1 enhanced silencing in the *LIMD1*^*−/−*^ line (Figure S1D). To explore the mechanism underlying the maintenance of silencing in the *LIMD1*^*−/−*^ line, we analyzed reporter activity when this isogenic HeLa CRISPR pair was treated with siRNAs targeting AGO1–4 (Figures 1B and 1C). We observed a switch in the requirement for AGO proteins in this pair of HeLa lines. AGO2 depletion only led to de-repression of miRNA silencing in the *LIMD1*^*+/+*^ line, whereas, in the *LIMD1*^*−/−*^ line, AGO3 depletion produced a de-repression. As AGO2 comprises the majority (∼60%) of the AGO pool in HeLa cells (Petri et al., 2011) and its levels remained unchanged upon LIMD1 loss (Figure S1C), the switch in AGO utilization may reflect the importance of LIMD1 specifically for AGO2 function in HeLa.

In order to interrogate the generality of this switch in AGO utilization, we employed a *let-7a*-targeted reporter. Similarly, we observed that loss of LIMD1 (*LIMD1*^*−/−*^) rendered AGO2 unable to contribute to silencing, whereas in *LIMD1*^*+/+*^ cells, de-repression was induced upon AGO2 knockdown (Figures S1E and S1F). In contrast to the miR-99/100 reporter, however, AGO1 and AGO3 knockdown resulted in de-repression of the *let-7a*-targeted reporter, regardless of *LIMD1* genotype, indicating this reporter and miRNA demonstrated a broader specificity for AGO and LIMD1 family proteins. To rule out the possibility that the observed switch in AGO use was due to the use of a synthetic 3′ UTR miRNA reporter, we also examined whether LIMD1 loss resulted in a switch in AGO requirement of two natural 3′ UTRs reporters—FGFR3 and MTOR, which each contain, in addition to other miRNA sites, single miR-99/100 sites (Figures 1H, 1I, S1G, and S1H). Both reporters showed a switch from AGO1/2 dependency, to AGO1/3 and AGO4, respectively, in the absence of LIMD1.

### LIMD1 Is Critical for the Interaction of AGO2 with TNRC6A and DDX6

**Figure 2:**
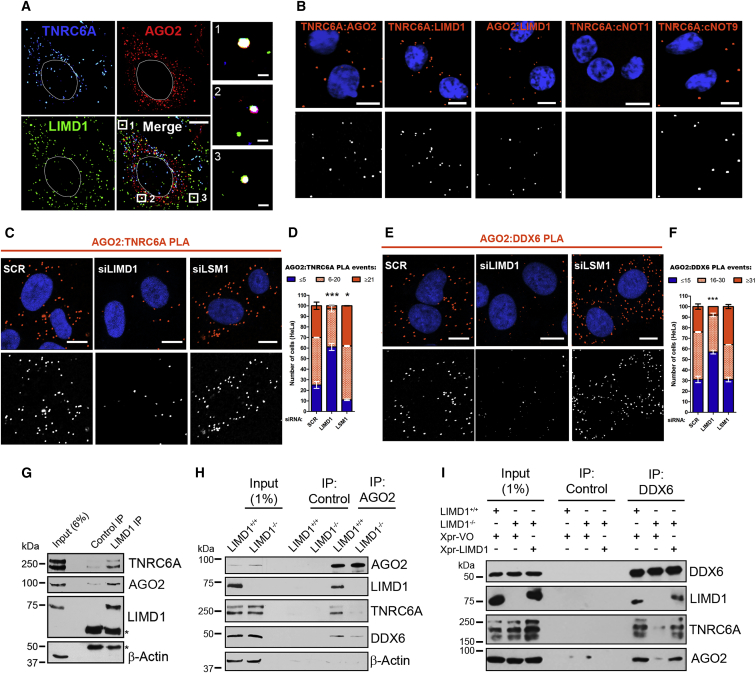
LIMD1 Interacts with miRISC Components AGO2 and TNRC6A and Promotes Their Association (A) Endogenous IF of the indicated proteins in HeLa cells. (B) Endogenous in situ interaction determined by proximity ligation assay (PLA) of the indicated proteins. PLA signal orange, cells stained with DAPI (top); PLA signal white for visual clarity (bottom). Scale bars, 10 μm. (C) PLA analysis of endogenous AGO2 and TNRC6A interaction in HeLa cells treated with SCR (non-targeting), LIMD1, or LSM1 siRNA. PLA signal orange, cells stained with DAPI (top); PLA signal white for visual clarity (bottom). (D) Quantification of PLA interaction events in (C), displayed as a stacked histogram. Data are mean ± SEM, n = 3, ^∗∗^p < 0.001, ^∗∗∗^p < 0.0001, determined using the chi-square test. (E) PLA analysis of endogenous AGO2 and DDX6 interaction, as in (C). (F) Quantification of (E) as in (D). (G) Endogenous co-immunoprecipitation of LIMD1 with the indicated proteins from HeLa cells (^∗^IgG heavy chain). (H) Endogenous coIP of indicated miRISC components with AGO2 from CRISPR-Cas9 gene-edited HeLa LIMD1^+/+^ or LIMD1^−/−^ cells. (I) Endogenous coIP of indicated miRISC components with DDX6 from CRISPR-Cas9 gene-edited HeLa LIMD1^+/+^ or LIMD1^−/−^ cells expressing Xpress (Xpr) vector only (VO) or LIMD1.

We next examined the binding partners and function of LIMD1 in miRISC in more detail to shed light on why loss of LIMD1 appeared to render AGO2 unable to contribute to silencing of both synthetic and natural 3′ UTRs and how the switch in AGO utilization was facilitated. Immunofluorescence (IF) staining of endogenous LIMD1, AGO2, and TNRC6A showed triple colocalization to processing bodies (P-bodies) (Figure 2A). To investigate direct endogenous association of AGO2 and TNRC6A with LIMD1 in situ, we performed proximity ligation assays (PLA) (Söderberg et al., 2006) for AGO2:LIMD1 and TNRC6A:LIMD1 (Figures 2B and S2A). We observed PLA signal for LIMD1 with both AGO2 and TNRC6A. In agreement with recent reports (Chen et al., 2014, Mathys et al., 2014), our PLA analysis demonstrated TNRC6A directly associated with CNOT9 (CNOT9 contains W-binding pockets to accommodate tryptophan residues from TNRC6A). By contrast CNOT1 did not exhibit PLA signal with TNRC6A; these data may be reflective of the lack of direct binding evidence between these proteins and the absence of a structurally resolved binding interface (Chen et al., 2014, Mathys et al., 2014). This supports the validity and specificity of the endogenous LIMD1 direct interactions with AGO2/TNRC6A.

LIM-domain-containing proteins are characterized by their ability to act as scaffolds in multi-protein complexes (Koch et al., 2012). We therefore reasoned that the close proximity and association observed between LIMD1, AGO2, and TNRC6A could indicate a scaffolding role for LIMD1 within the miRISC. We examined the endogenous colocalization of AGO2 with TNRC6A and miRISC component DDX6 by IF in HeLa cells upon treatment with siRNA targeting *LIMD1* (Figures S2B–S2D). LSM1 is required for the assembly of P-bodies but does not affect miRNA-silencing function; we therefore included siRNA targeting LSM1 as a control for P-body disruption. LIMD1 depletion did not disrupt the colocalization of AGO2 with TNRC6A or DDX6. By contrast, and as described in the literature, LSM1 knockdown caused a decrease in visible AGO2/TNRC6A/DDX6 P-bodies (Chu and Rana, 2006).

To rule out a possible false-negative effect due to the limited resolution of confocal microscopy (240 nm) (Nature Photon, 2009), we repeated our analysis using PLA, which detects proteins within 40 nm of each other (Figures 2C–2F). PLA analysis demonstrated that interaction of AGO2 with TNRC6A and DDX6 was significantly reduced upon LIMD1 knockdown, whereas LSM1 knockdown did not reduce this interaction, and in fact increased interaction of AGO2 with TNRC6A. In agreement with these data, reporter assays performed in cells treated with the same siRNAs demonstrated significant de-repression of a miR-99/100 reporter upon loss of LIMD1 but not LSM1 (Figure S2E). These findings confirm that assembly of P-bodies is a consequence rather than a cause of miRNA silencing (Eulalio et al., 2007) and demonstrate that the de-repressive effect of LIMD1 depletion on miRNA silencing is not dependent on the disassembly of P-bodies. Together, our data show that LIMD1 contributes to maintaining the interactions of AGO2 with TNRC6A and DDX6.

In order to further pursue the discrepancy between protein localization to P-bodies and the interaction between miRISC proteins, we performed endogenous IF for AGO1, 2, 3, and 4 with both TNRC6A and DDX6 in the isogenic *LIMD1*^*−/−*^ HeLa CRISPR pair (Figures S3A and S3B). Loss of LIMD1 had no visible effect on AGO1, 2, or 3 colocalization with TNRC6A or DDX6 in P-bodies (AGO4 did not colocalize with TNRC6A/DDX6 in either cell line). Furthermore, we did not detect any change in colocalization of EYFP-tagged AGO2 with endogenous TNRC6A upon inhibition of Akt3 by siRNA, treatment with Akt inhibitor MK-2206, or overexpression of AGO2 S387A point mutant (Figure S4A). We therefore performed complementary PLA and endogenous immunoprecipitation experiments and observed strikingly that *LIMD1* knockout caused significant impairment of endogenous AGO2 interaction with TNRC6A and DDX6 (Figures S4B–S4G), in agreement with functional reporter assays performed in these lines (Figure 1). Furthermore, re-expression of Xpress (Xpr)-tagged LIMD1 in the *LIMD1*^*−/−*^ line restored the interaction of DDX6 with AGO2 and TNRC6A (Figure 2I), potentially explaining the enhancement of silencing observed when LIMD1 is overexpressed in a *LIMD1*-null background (Figure S1D). These data therefore demonstrate that endogenous interaction assays, including PLA and IP, are more accurate methods of determining endogenous miRISC protein-protein interaction, as opposed to visible colocalization within P-bodies. In summary, loss of LIMD1 profoundly impaired the ability of AGO2 to interact with TNRC6A and downstream effectors within miRISC required for miRNA-mediated silencing.

### LIMD1 Interacts with AGO2 and TNRC6A via Specific Domains

**Figure 3:**
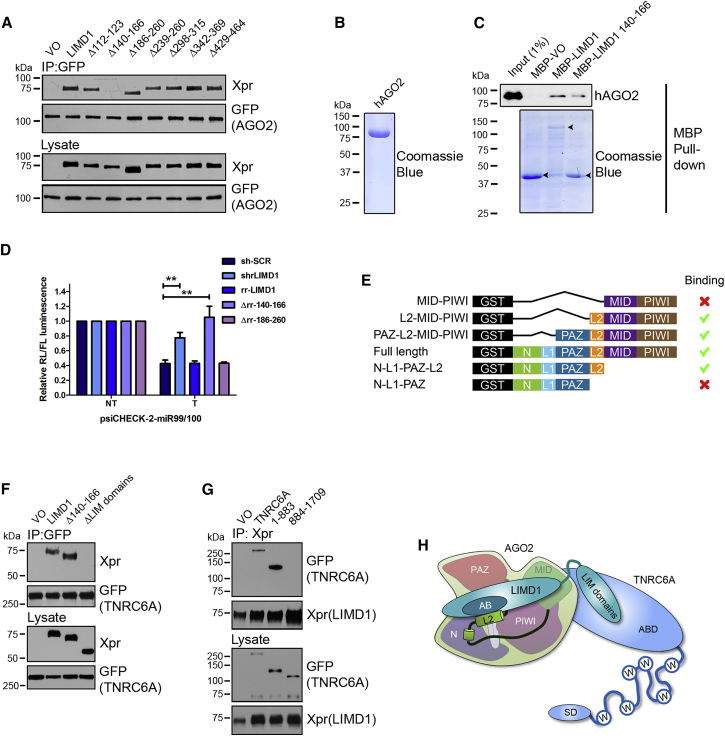
LIMD1 Bridges AGO2 and TNRC6A via Distinct Interaction Sites (A–C) (A) Co-immunoprecipitation (coIP) of Xpress (Xpr)-LIMD1 and internal deletion mutants with GFP-AGO2 from HEK293T lysate. (B) Purified un-tagged crystallography grade hAGO2 used in (C) direct binding assay with MBP-VO, MBP-LIMD1, or MBP-LIMD1 140 166 (AB motif) (highlighted by arrows). (D) psiCHECK-2-miR99/100 luciferase reporter (NT [non-targeting], T [targeting]) assay performed in shRNA knockdown and rescue (RNAi resistant [rr]) LIMD1 HeLa cells (mean ± SEM; n = 3). (E) Schematic of GST-AGO2 truncation mutants and summary of binding to LIMD1 AB motif. (F) CoIP of Xpr-LIMD1 and deletion mutants with GFP-TNRC6A from HEK293T lysate. (G) CoIP of TNRC6A and truncation mutants with Xpr-LIMD1 from HEK293T lysate. (H) Graphical representation of domain interaction of AGO2, LIMD1, and TNRC6A. The AB motif of LIMD1 (aa 140–166) directly binds linker 2 (L2) of AGO2, and the LIM domains of LIMD1 bind the N-terminal AGO-binding domain (ABD) of TNRC6A (SD, silencing domain).

Having demonstrated that LIMD1 is required to scaffold the interaction of AGO2 and TNRC6A, we next sought to map the interaction interfaces on all three proteins. LIMD1 contains three tandemly arrayed LIM domains (protein-protein interacting zinc fingers) in its C-terminal region and a preceding proline/serine-rich unstructured domain referred to as the pre-LIM (residues 1–471). Our previous work demonstrated that AGO2 bound to the pre-LIM portion of LIMD1 (James et al., 2010). To identify the precise AGO Binding (AB) motif in the pre-LIM, we co-immunoprecipitated Xpr-tagged full-length LIMD1 or a series of LIMD1 internal deletion mutants with EYFP-tagged AGO2. We found that deletion of amino acids 140–166 within LIMD1 abrogated the interaction with AGO2 (Figure 3A). The identification of amino acids 140–166 as the AB motif in LIMD1 was confirmed by direct binding assays using purified un-tagged AGO2 (hAGO2) (De and Macrae, 2011) (Figure 3B) and maltose binding protein (MBP)-tagged full-length LIMD1 or AB motif (Figure 3C). Generation of a set of HeLa lines lentivirally transduced to express sh-SCR (non-targeting) or sh-LIMD1 RNA and RNAi-resistant wild-type (WT) LIMD1 (rr-LIMD1) or LIMD1 deletion mutants (Figure S5A) demonstrated loss of silencing of the miR-99/100 reporter in sh-LIMD1 and rr-Δ140–166 lines (Figure 3D). In contrast, we observed that cells expressing the larger adjacent rr-Δ186–260 LIMD1 deletion mutant (which retains AGO2 binding, Figure 3A) displayed equivalent levels of silencing to that seen in rr-LIMD1 cells, thus providing an additional control for the impact of internal deletions on the secondary structure of the pre-LIM domain. These data indicate that, as predicted, the ability of LIMD1 to bind AGO2 via its AB motif is critical for its miRNA-silencing function.

We next performed complementary direct binding assays to identify the corresponding LIMD1-binding domain within AGO2, using a series of GST-AGO2 N- and C-terminal truncation mutants (Figures S5B and S5C). We determined that the Linker 2 (L2) domain of AGO2, which lies between the PAZ and MID domains, was necessary for direct interaction with the AB motif of LIMD1 (MBP-LIMD1 140–166) and full-length LIMD1 (Figure 3E). We note that the additional bands present in these purified AGO2 mutants are either degradation products (as determined by western blot and mass spectrometry (Figures S5D–S5G) or bacterial Hsp70 (DnaK), which did not affect binding to LIMD1 (Figure S5H).

As our PLA experiments also indicated a close association of LIMD1 with TNRC6A, we sought to confirm whether this occurred independently of AGO2. We found that deletion of the LIM domains (C-terminal) of LIMD1 resulted in loss of interaction with EGFP-tagged TNRC6A in co-immunoprecipitation experiments (Figure 3F). Deletion of the LIMD1 AB motif did not affect interaction with TNRC6A, demonstrating that the interaction of LIMD1 with TNRC6A is not dependent upon LIMD1 interaction with AGO2. Next, we determined that the N-terminal AGO-binding domain (ABD) of TNRC6A (Eulalio et al., 2009b) was responsible for binding to LIMD1; coIP experiments in HEK293T cells using EGFP-tagged TNRC6A truncations and Xpr-LIMD1 revealed that deletion of TNRC6A amino acids 1–883 resulted in complete loss of binding to LIMD1 (Figure 3G).

In summary, the pre-LIM region of LIMD1 is responsible for interaction with AGO L2 (via the AB, aa 140–166), and the C-terminal LIM domains interact with the N-terminal portion of TNRC6A. Therefore, we provide evidence that LIMD1 functions as a central scaffold protein and a core component of miRISC, forming an integral link between AGO2 and TNRC6A through simultaneous direct contacts with each (Figure 3H).

### Akt3-Mediated Phosphorylation of S387 in the AGO2 L2 Domain Is Necessary for Interaction with LIMD1

**Figure 4:**
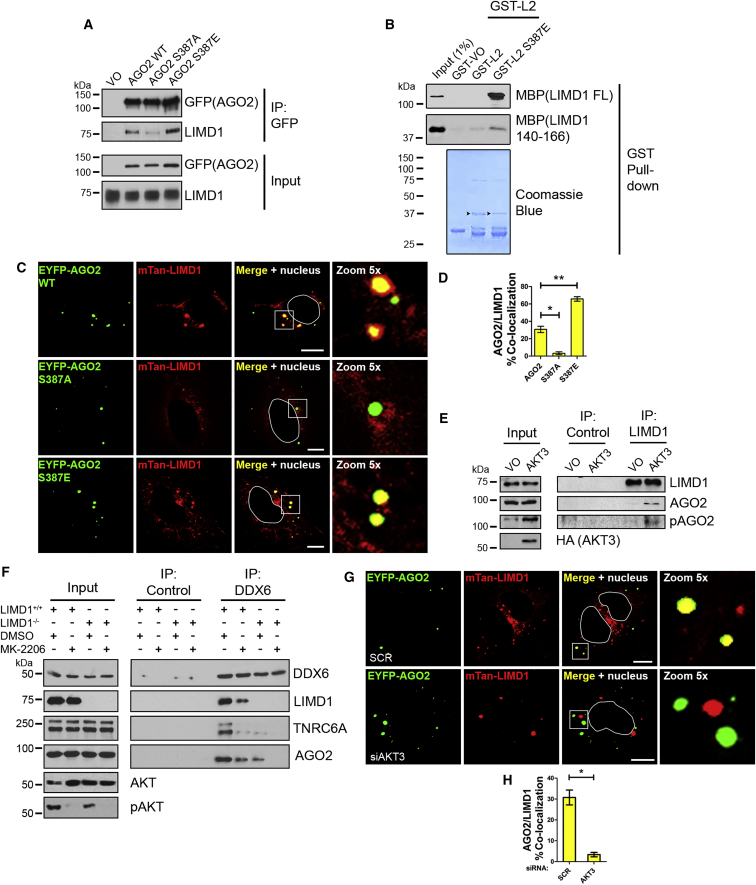
Binding of LIMD1 to AGO2 Is Dependent on Akt3-Mediated S387 Phosphorylation (A) Co-immunoprecipitation (IP) of Xpr-LIMD1 with EYFP-AGO2 WT, AGO2S387A (phospho-deficient), and AGO2S387E (phospho-mimic) point mutants from HEK293T lysate. (B) Purified recombinant GST-AGO2 L2 or L2 S387E were used in direct binding assay with MBP-LIMD1 full-length or AB motif (140–166). (C) Co-localization of EYFP-AGO2/S387 point mutants with mTan-LIMD1 in U2OS cells. Nucleus outline in white. (D) Quantification of percentage of AGO2/S387 P-bodies colocalized with LIMD1 in (C). (E) Immunoprecipitation of endogenous LIMD1 from HeLa cells transfected with HA-VO or HA-myristolated Akt3, and western blot analysis of the indicated proteins; pAGO2 is S387. (F) Immunoprecipitation of DDX6 from HeLa LIMD1^+/+^ or LIMD1^−/−^ treated with DMSO or Akt inhibitor MK-2206 and analyzed for the indicated proteins by western blot; pAKT is S473. (G) Co-expression of EYFP-AGO2 (WT) with mTan-LIMD1 in U2OS cells treated with non-targeting (SCR) or AKT3 siRNA. Nuclear outlines in white. (H) Quantification of number of AGO2 P-bodies colocalized with LIMD1 in (G). Data shown are mean ± SEM, n = 3, ^∗^p < 0.05, ^∗∗^p < 0.001. Scale bars, 10 mm.

It has been proposed that Akt3-dependent phosphorylation of AGO2 S387 promotes its miRNA function and opposes its target cleavage activity (Horman et al., 2013). AGO2 S387 phosphorylation was suggested to increase the association of AGO2 with TNRC6A via an unknown mechanism. As S387 is located within the L2 region of AGO2, we tested whether phosphorylation of this residue could trigger the association between AGO2 and LIMD1 and subsequent recruitment of TNRC6A to AGO2. We generated constructs expressing phospho mutant (AGO2 S387A) or phospho-mimic (AGO2 S387E) versions of EYFP-AGO2 and compared their ability to interact with LIMD1 in immunoprecipitation assays. AGO2 S387A had reduced binding to LIMD1, whereas the phospho-mimic version of AGO2 (AGO2 S387E) rescued interaction with LIMD1 (Figures 4A and S6B). The interaction profile of LIMD1 with these AGO2 constructs was mirrored by endogenous TNRC6A and DDX6, which displayed significantly reduced binding to AGO2 S387A, and rescue of binding with AGO2 S387E (Figures S6A and S6B). The importance of S387 phosphorylation for LIMD1 binding was further confirmed by direct binding assays between the AB motif of LIMD1 and GST-AGO2 WT or GST-AGO2 S387E, which revealed enhanced binding of the AGO2 phospho mimic to LIMD1 AB motif (Figure 4B). The dependency on S387 for LIMD1 association was also evident in the degree of colocalization of AGO2 and LIMD1 in U2OS cells transfected with mTan-LIMD1 and the EYFP-AGO2 constructs (Figures 4C, 4D, and S6C). Compared to WT AGO2, AGO2 S387A and S387E showed a 9.4-fold decrease and 2.1-fold increase in colocalization with LIMD1, respectively. These data indicate that the interaction of AGO2 with LIMD1 is dependent on the phosphorylation status of AGO2 S387.

Next, we tested whether the interaction between LIMD1 and AGO2 could be directly promoted by overexpression of Akt3, thought to be the primary kinase responsible for phosphorylation of AGO2 S387 (Horman et al., 2013). We immunoprecipitated endogenous LIMD1 from serum-starved HeLa cells transfected with an empty vector or a constitutively active myristoylated Akt3 (HA-myr-Akt3) (Song et al., 2008) and found that Akt3 significantly enhanced both the levels of phospho-S387 AGO2 (using a phospho-S387-specific antibody (Zeng et al., 2008; Figure S6D) and the interaction of AGO2 with LIMD1 (Figure 4E). In a complementary assay, treatment of the HeLa CRISPR-Cas9 isogenic pair with the Akt inhibitor MK-2206 demonstrated that interaction of AGO2, LIMD1, and TNRC6A with DDX6 was significantly impaired in *LIMD1*^+/+^ cells upon Akt inhibition (Figure 4F); under these conditions, the interaction of the complex was equivalent to that of the *LIMD1*^−/−^ line (Figure S6E). Treatment of the *LIMD1*^−/−^ cells with MK-2206 entirely abolished the complex interactions, indicating that there is some residual interaction of AGO2 with miRISC in the absence of LIMD1, which is also Akt dependent. In agreement with these assays, siRNA-mediated knockdown of Akt3 in U2OS cells resulted in a 9-fold reduction in colocalization between EYFP-AGO2 and mTan-LIMD1 (Figures 4G, 4H, and S6F). In summary, these data confirm that Akt3-mediated phosphorylation of AGO2 S387 is required for AGO2-LIMD1 interaction.

### Activated Phospho-S387-AGO2 Requires LIMD1 for Formation of a Functional AGO2-miRISC

**Figure 5:**
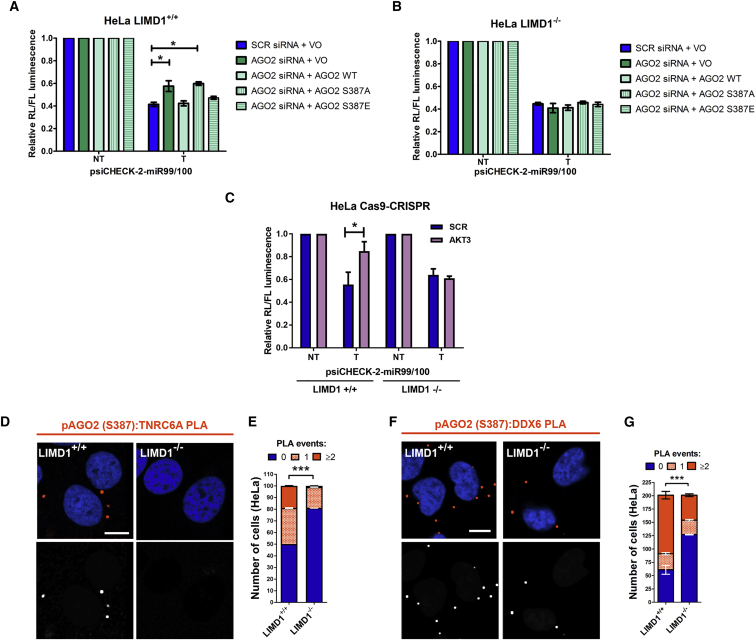
LIMD1 Is Required for Phospho-S387-AGO2 to Engage TNRC6A/DDX6 for miRNA-Mediated Silencing (A and B) (A) psiCHECK-2-miR99/100 reporter assay in HeLa CRISPR-Cas9 LIMD1^+/+^ or (B) LIMD1^−/−^ cell lines transfected with non-targeting (SCR) or AGO2 siRNA and EYFP-AGO2, EYFP-AGO2S387A, or EYFP-AGO2S387E. (C) miR99/100 reporter assay in in above HeLa CRISPR-Cas9 cell lines transfected with non-targeting (SCR) or AKT3 siRNA. (D) PLA analysis of endogenous phospho-AGO2 (S387):TNRC6A interaction in situ in HeLa CRISPR-Cas9 LIMD1^+/+^ or LIMD1^−/−^ lines. PLA signal orange, cells stained with DAPI (top); PLA signal white for visual clarity (bottom). Scale bars, 10 μm. (E) Quantification of PLA interaction events in (D), displayed as stacked histograms. Data are mean ± SEM, n = 3, total of 200 cells determined using the chi-square test. (F) PLA analysis of phospho-AGO2 (S387):DDX6 interaction in above HeLa CRISPR-Cas9 cell lines. (G) Quantification of (F) as in (E). Unless otherwise stated, data are mean ± SEM, n = 3, ^∗^p < 0.05, ^∗∗^p < 0.001, ^∗∗∗^p < 0.0001 according to the Student’s t test.

To determine whether phosphorylated AGO2 (S387) is dependent on LIMD1 for miRISC formation and function, we examined the activity of the miR-99/100 reporter in HeLa CRISPR lines treated with siRNA against AGO2, where WT AGO2 or S387 phospho-site mutants were re-expressed (Figures 5A, 5B, and S6G). In *LIMD1*^*+/+*^ cells, AGO2 knockdown caused the expected de-repression. Re-expression of AGO2 WT and S387E rescued silencing, whereas AGO2 S387A was unable to rescue silencing, in agreement with published data (Horman et al., 2013). In contrast, in the *LIMD1*^*−/−*^ line, AGO2 knockdown did not cause any de-repression, and re-expression of any AGO2 construct (WT or S387 mutant) similarly had no effect.

We next determined whether the previously established regulation of AGO2 miRNA silencing by Akt3 was in fact mediated by LIMD1 (Horman et al., 2013). Upon siRNA-mediated depletion of Akt3, we observed de-repression of the miR-99/100 reporter in *LIMD1*^*+/+*^ cells, but this had no effect in the *LIMD1*^*−/−*^ line, where silencing was maintained (Figures 5C and S6H). Taken together, these data demonstrate that the activating effect of Akt3-mediated phosphorylation on AGO2 is dependent on subsequent interaction with LIMD1. In the absence of LIMD1, miRNA silencing still occurs independently of Akt3. To investigate how LIMD1 loss caused this striking inhibition of phospho-AGO2 activity, we examined the interaction of phospho-AGO2 with TNRC6A and DDX6 upon loss of LIMD1. PLA analysis with the phospho-AGO2 (S387)-specific antibody demonstrated that loss of LIMD1 significantly disrupts the ability of phospho-AGO2 to bind both TNRC6A and DDX6 endogenously (Figures 5D–5G, S6I, and S6J). These data therefore demonstrate that AGO2 phosphorylation by Akt3 has no activating effect on miRNA silencing in the absence of LIMD1 due to the fact that the loss of this scaffold protein dissociates phospho-AGO2 from TNRC6A/miRISC and precludes AGO2-dependent miRNA silencing. These data are in agreement with the functional observations made upon LIMD1 loss (Figure 1), where we observed functional replacement by AGO3. We therefore went on to examine whether this mechanism of phospho-dependent recruitment of LIMD1 family proteins was conserved across all human AGO proteins.

### AGO1 and AGO4 Exhibit Phosphorylation-Dependent Interaction with LIMD1 Family Proteins

**Figure 6:**
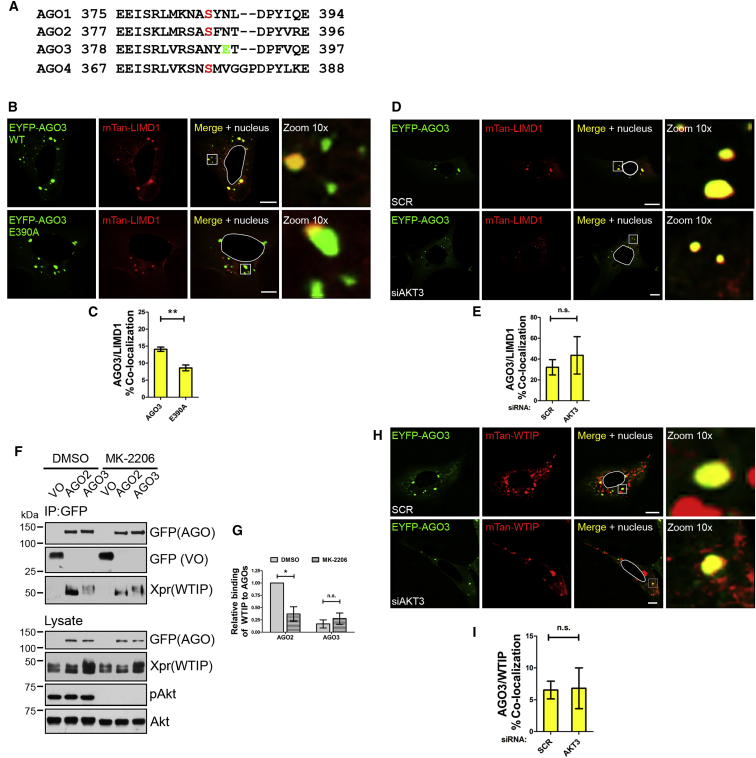
AGO3 E390 Acts as a Phospho-mimic, Facilitating Interaction with LAW Independently of Akt Signaling (A) AGO2 S387 is highly conserved within AGO1 (S385) and AGO4 (S377). AGO3 does not contain an equivalent serine residue but instead an adjacent phospho-mimic glutamic acid residue (E390). (B) Colocalization of EYFP-AGO3/E390 point mutants with mTan-LIMD1 in U2OS cells. (C) Quantification of percentage of AGO3 P-bodies colocalized with LIMD1 in (B). (D) Colocalization of EYFP-AGO3 with LIMD1 in U2OS cells treated with non-targeting (SCR) or AKT3 siRNA. (E) Quantification of percentage of AGO3 P-bodies colocalized with LIMD1 in (D), n = 2. (F) Immunoprecipitation (IP) of EYFP-VO, -AGO2, or -AGO3 from HEK293T lysate co-transfected with Xpress (Xpr)-WTIP and treated with DMSO or Akt inhibitor MK-2206. (G) Quantification of WTIP interaction with AGO2 and 3, in (F). Data are mean densitometry relative to AGO2 (DMSO), ±SEM, n = 3. (H) Colocalization of EYFP-AGO3 with WTIP in U2OS cells treated with non-targeting (SCR) or AKT3 siRNA. (I) Quantification of percentage of AGO3 P-bodies colocalized with WTIP in (H), n = 2. Unless otherwise stated, quantification data are mean ± SEM, n = 3, ^∗^p < 0.05, ^∗∗^p < 0.01, ^∗∗∗^p < 0.001, n.s., not significant.

A significant structural homology exists between the four human AGO proteins; our alignment analysis revealed that AGO2 S387 is conserved in the L2 domains of AGO1 and AGO4 (Figure 6A). We therefore investigated whether there was a conserved phospho-dependent mechanism of AGO1, 2, and 4 interaction with LIMD1 family members. First, we found that LIMD1, Ajuba, and WTIP interacted with all four AGOs (Figures S7A, S7B, and data not shown). LIMD1 also directly bound to the L2 domain of AGO1 (Figure S7C), and, in a similar manner to AGO2, mutation of the conserved serine in AGO1 (S385) to alanine inhibited colocalization with LIMD1, whereas mutation to glutamic acid rescued this colocalization (Figures S7D and S7F). Furthermore, knockdown of Akt3 by siRNA significantly reduced AGO1:LIMD1 colocalization, suggesting that Akt3 may also phosphorylate AGO1 S385 (Figures S7E and S7G). Point mutation of the conserved serine (S377) in AGO4 produced the same disruption and rescue of colocalization with LIMD1 as observed with AGO1 and 2 (Figures S7H and S7J). Taken together these data suggest a common mechanism may exist whereby human AGO 1, 2 and 4 are activated for miRNA-silencing function by phosphorylation of a conserved serine residue, which directs their interaction with LIMD1 and family members and subsequent recruitment to TNRC6A/miRISC. Given the ability of LIMD1 to colocalize with three AGO proteins in a phospho-dependent manner, we also demonstrated that the LIMD1 family member Ajuba, also colocalized with AGO1 and that this was dependent on the phosphorylation status of S385 (Figure S7I). These data support a fundamentally conserved mechanism of phosphorylation-dependent activation of AGO proteins by recruitment and regulation of all three members of this LIM-domain-containing family, LIMD1, Ajuba, and WTIP.

### AGO3 Interacts with LIMD1 Family Proteins Independently of Akt3 Phosphorylation

In contrast to AGO1 and AGO4, AGO3 possesses a phosphorylation-mimicking glutamic acid residue at amino acid position 390 (Figure 6A). Given that we found AGO3 can rescue loss of AGO2 miRNA function in the absence of LIMD1, we postulated that this phospho-mimic residue may facilitate interaction with LIMD1 and/or its closely related family members Ajuba/WTIP independently of Akt3 signaling. This could be a mechanism explaining the switch to AGO3 utilization upon deletion of LIMD1 (Figure 1).

To interrogate this hypothesis, we first determined that AGO3 colocalizes with LIMD1, consistent with IP data (Figure S7A), and that this is significantly reduced upon mutation of the phospho-mimic residue to alanine (E390A) (Figures 6B and 6C). Similarly, colocalization of AGO3 with Ajuba was reduced by mutation of E390 to alanine (Figures S7K and S7L). In striking contrast to AGO2, siRNA-mediated knockdown of Akt3 did not affect AGO3/LIMD1 colocalization (Figures 6D and 6E). WTIP interaction with AGO3 was also unaffected by Akt inhibition (MK-2206), as opposed to AGO2, which demonstrated loss of interaction (Figures 6F and 6G), and siRNA-mediated knockdown of Akt3 similar had no effect on AGO3 colocalization with WTIP (Figures 6H and 6I). These data therefore demonstrate that AGO3 interaction with LIMD1 family proteins occurs independently of Akt signaling as a result of the naturally occurring phospho-mimic residue E390.

**Figure 7:**
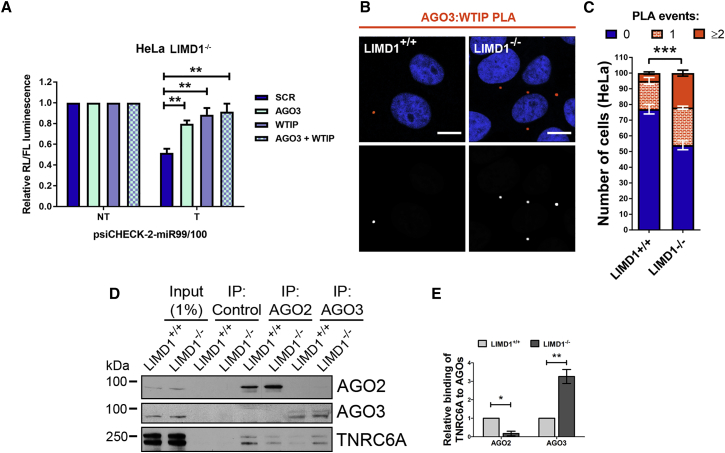
AGO2 miRNA Function Switches to AGO3 with LIM Family Member WTIP in the Absence of LIMD1 (A) LIMD1^−/−^ HeLa cells transfected with non-targeting (SCR) and the indicated siRNAs (^∗∗^p<0.01 according to student's T-test). (B) PLA of AGO3 and WTIP in HeLa CRISPR-Cas9 cell lines. (C) Quantification of PLA interaction events in (B), displayed as a stacked histogram. (D) IP of endogenous AGO2 or AGO3 from HeLa CRISPR-Cas9 cells, coIP of endogenous TNRC6A. (E) Quantification of TNRC6A coIP with AGO2 and 3 in HeLa CRISPR-Cas9 cells in (D). Data are mean densitometry relative to LIMD1^+/+^ AGO2/3. Unless otherwise stated, all data are ±SEM, n = 3, ^∗^p < 0.05, ^∗∗^p < 0.001, ^∗∗∗^p < 0.0001, n.s., not significant, according to the Student’s t test.

We observed activation of AGO3 miRNA function when LIMD1 protein expression was entirely ablated (Figure 1), and phosphorylated AGO2 was unable to engage with TNRC6A and silencing effectors (Figure 5). In the *LIMD1*^*−/−*^ HeLa cell line, siRNA depletion of WTIP yielded a significant de-repression of the miR-99/100 reporter, whereas Ajuba knockdown did not (data not shown). Therefore, to determine whether WTIP was responsible for engaging AGO3 in the absence of LIMD1, we performed siRNA knockdown of AGO3 or WTIP, singularly or in combination, in the *LIMD1*^*−/−*^ HeLa cell line. We discovered that WTIP is required for silencing of a miR-99/100 reporter in these cells (Figure 7A). Furthermore, depletion of WTIP and AGO3 in combination led to an almost complete de-repression of silencing (Figure 7A). Concurrent with this, we discovered an increase in the number of AGO3 P-bodies in the *LIMD1*^*−/−*^ line compared to *LIMD1*^*+/+*^ (Figures S7M and S7N), indicating that while a reduction in an AGO miRNA-silencing function is not necessarily associated with dissociation from P-bodies (as is the case with AGO2), an increase in AGO3 silencing function may be associated with an increase in its recruitment to P-bodies. Furthermore, we observed an increase in AGO3:WTIP interaction in the *LIMD1*^*−/−*^ line compared to *LIMD1*^*+/+*^ (Figures 7B and 7C).

AGO function in miRNA silencing is critically dependent on association with TNRC6 proteins, which enable recruitment of several effector complexes that mediate target mRNA decapping and deadenylation. We therefore examined whether TNRC6A recruitment to AGO2 and AGO3 was altered in the presence and absence of LIMD1, which would explain the apparent switch in AGO function. Endogenous immunoprecipitation of AGO2 or AGO3 from the HeLa *LIMD1*^*−/−*^ CRISPR cell line pair demonstrated that, in contrast to the loss of TNRC6A interaction with AGO2, AGO3 interaction with TNRC6A was significantly enhanced upon loss of LIMD1 (Figures 7D and 7E).

These interaction data elucidate the mechanism by which loss of LIMD1 causes a switch in cellular AGO dependency (Figure 1). We have discovered that, in HeLa cells, loss of LIMD1 causes dependency upon AGO2 to switch to AGO3, and this is associated with enhanced (phosphorylation-independent) interaction of AGO3 with LIMD1 family member WTIP, and with TNRC6A as the effector of silencing. These data therefore define the mechanism by which AGO2-S387 phosphorylation is an activating event for silencing. Additionally, as all LIMD1 family proteins interact with AGO1–4 in a phospho-dependent manner, these data suggest a general mechanism whereby specific AGO proteins are activated for miRNA function by their phosphorylation and subsequent recruitment of LIMD1 family proteins, thus enabling miRISC formation.

## Discussion

### LIMD1 Acts as a Molecular Clamp to Secure Phospho-AGO2 and TNRC6A Together within miRISC

We determined that LIMD1 binds directly to AGO2 and TNRC6A at distinct interfaces: the AGO-binding motif (AB motif) of LIMD1 binds the L2 domain of AGO2, and the C-terminal LIM domains of LIMD1 bind the amino-terminal ABD of TNRC6A, independently of AGO binding (Figure 3) (Eulalio et al., 2009a, Pfaff et al., 2013, Takimoto et al., 2009). It is important to note that our data do not suggest the AGO-TNRC6A association can only occur via LIMD1; the MID/PIWI (P-element induced wimpy testis) domains of AGO2 that directly bind TNRC6A tryptophan residues do not bind to LIMD1 (Figure 3E) (Schirle and MacRae, 2012). Additionally, the association of the C-terminal silencing domain of TNRC6A with PABP/CNOT9/CNOT1/DDX6 does not preclude the binding of LIMD1, which interacts with the N-terminal half of TNRC6A (Figure 3D) (Eulalio et al., 2009a). Therefore, our data indicate that LIMD1 acts as a molecular “clamp” by binding to both AGO and TNRC6A proteins simultaneously to secure their association and commit both proteins and associated effectors to the miRNA-silencing pathway: AGO2 association with TNRC6A/miRISC and its miRNA-silencing activity is significantly impaired upon LIMD1 loss (Figures 1A–1C and 2).

It has been demonstrated that activation of miRNA silencing is dependent on Akt3-mediated phosphorylation of AGO2 at S387 (Horman et al., 2013). We have determined that this phosphorylation promotes association of AGO2 with LIMD1 and subsequent recruitment of TNRC6A and downstream silencing effector complexes (Figures 4 and 5). Our findings therefore reveal the functional consequence of S387 phosphorylation, which underpins an apparent preference of AGO utilization directed by LIMD1 family member proteins. In agreement with our findings, La Rocca et al. recently identified phosphorylation-dependent activation of miRNA silencing by PI3K, a kinase that lies upstream of Akt3 (La Rocca et al., 2015). Although this study did not examine the precise changes in AGO2 phosphorylation, it discovered an increase in the molecular mass of AGO2 upon pathway activation, most likely via the recruitment of other effector proteins, which would include TNRC6A, DDX6, and, from our data, LIMD1.

### AGO Interaction with LIMD1 Family Proteins Occurs via a Common Phosphorylation-Dependent Mechanism

The AGO L2 domain is a 98-residue, extended L-shaped coil that connects the PAZ and MID domains of AGO proteins (Elkayam et al., 2012, Schirle and MacRae, 2012). The AGO3 S387 residue phosphorylated by Akt3 is conserved in human AGO1 and 4 (Figure 6A). Our data demonstrate that LIMD1 can interact with all four AGO proteins (Figure S8A) and that association with AGO1 and 4 is also dependent on the ability of this conserved serine residue to be phosphorylated (Figures S8D–S8I). Taken together with previous studies demonstrating that AGO1 is phosphorylated (Rüdel et al., 2011), these data support a hypothesis of a common AGO-activating mechanism by Akt3 and/or other unidentified kinases. AGO3 does not contain this conserved serine residue but rather contains a phospho-mimic glutamic acid residue, which directs interaction with LIMD1 family members independently of Akt signaling (Figure 7).

Horman et al. postulated that phosphorylation of S387 represented a molecular switch between the siRNA and miRNA function of AGO2 (Horman et al., 2013). However, our data indicate the Akt3-mediated phosphorylation of AGO1, 2 (and possibly AGO4) could also be considered as a switch from OFF to ON for miRNA-silencing function. Furthermore, if one were to consider the possibility of a cell state with no Akt3 signaling, where silencing may only be possible through AGO3 function, then an activation of Akt3 could activate a switch from the default AGO3 silencing to any or all of the AGO1-, 2-, or 4-dependent mechanisms. Our work also opens up the possibility for functional differences between the four human AGO proteins that have been previously been described to act redundantly in the miRNA pathway (Dueck et al., 2012, Su et al., 2009). Future work will address this possibility.

Although many components and processes of the miRNA pathway are highly conserved between humans and *Drosophila*, an important difference is that, in *Drosophila*, only one of two AGO proteins, AGO1, functions in the miRNA pathway (Förstemann et al., 2007, Tomari et al., 2007). Within the L2 domain of *Drosophila* AGO1, S387 is not conserved. This may suggest that the evolution of S387 in human AGO2 (and equivalent serines in AGO1 and 4) was necessary for coordinating the responses of multiple AGOs to signaling inputs. It remains to be determined whether a protein in *Drosophila* fulfills the role of LIMD1 with regard to promoting interaction between AGO1 and GW182. Recent data from Golden et al. have shown that cyclic phosphorylation at highly conserved serine residues in the PIWI domain of human AGO2 (S824–S834) promotes efficient miRNA silencing (Golden et al., 2017). They noted that these residues are conserved in human AGOs 1–4 and *Drosophila* AGO1 but not in *Drosophila* AGO2, which only functions in the siRNA pathway. Thus, the evolution of several regulatory phospho sites, including S387, may be integral to the expansion of the AGO gene family between fly and human, all of which can function in miRNA-mediated silencing.

### LIMD1 Directs AGO Family Member Utilization for miRNA Silencing

While siRNA and shRNA-mediated knockdown of proteins is able to provide functional insights, the complete genetic ablation of a protein can reveal longer-term adaptations in cells and alternative pathways to maintain homeostasis. Through the use of CRISPR-Cas9 technology, we ablated LIMD1 expression and observed a switch in AGO dependency for silencing from AGO2 to AGO3. In the *LIMD1*^*−/−*^ cell line, we found that phospho-AGO2 S387 and total AGO2 interaction with TNRC6A and DDX6 was significantly impaired, and AGO2 miRNA function was entirely ablated irrespective of its phosphorylation status (Figure 5). In parallel assays, LSM1, which is required for P-body assembly but not miRNA silencing (Chu and Rana, 2006, Eulalio et al., 2007, Eulalio et al., 2009a), affected neither AGO2 association with TNRC6A/DDX6 nor AGO2-dependent miRNA silencing (Figures 2C–2F). These data indicate that the effects observed upon LIMD1 loss occur specifically as a result of its scaffold role in the AGO2:TNRC6A association and not as a general effect of loss of P-body structural integrity. Indeed, re-expression of LIMD1 in the *LIMD1*^*−/−*^ line not only enhanced silencing, but also rescued the interaction of AGO2 with TNRC6A and DDX6 (Figure 2J).

The contributions of each AGO protein to miRNA-mediated silencing has been attributed to their expression levels, with AGO2 being the most abundant and therefore of greatest importance (Wang et al., 2012). However, our data reveal that LIMD1 family proteins are important determinants of AGO utilization and show that AGO levels per se may not necessarily determine functional importance. The typically low AGO3 expression in various tissues and cell types has been ascribed to the high proportion of rarely used codons in its mRNA (Valdmanis et al., 2012). Despite this, we have shown AGO3 does function in miRNA silencing and specifically functions in the absence of the Akt3-AGO2-LIMD1 signaling axis. Determination of AGO utilization and the cooperation of different AGOs may, in addition to specific pairing with LIMD1 family proteins, be context dependent with respect to miRNA site geometry in the 3′ UTR and the distribution of a particular miRNA among AGO proteins expressed in the cell. These considerations warrant further investigation to more clearly define the regulation of miRNA-dependent silencing.

We may speculate that, for our miR-99/100 reporter, in the presence of LIMD1, the majority of targeted sites on the reporter mRNA are occupied by AGO2-LIMD1 with a minor contribution of AGO3-WTIP. Potential interactions between LIMD1 and WTIP may be required for overall stability of the multiple RISCs assembled on this 3′ UTR, and short-term loss of either one (in the context of siRNA knockdown) would lead to significant de-repression of silencing. With LIMD1 loss, AGO3-WTIP may increase in occupancy of remaining/free sites on the reporter thereby rendering silencing highly dependent on AGO3.

Loss of LIMD1 by genetic ablation did not affect silencing of the *let-7a* reporter, but the underlying AGO utilization was altered, with AGO2 no longer contributing to silencing (Figures S1E and S1F). In this respect, as seen with the miR-99/100 reporter, long-term loss of LIMD1 led to functional adaptation to maintain miRNA silencing and also revealed the particular involvement of LIMD1 in AGO2-dependent miRNA silencing. The *let-7a* reporter contained six tandem *let-7a* sites, and its repression was dependent on AGO1, 2, and 3. This result suggests that, for this particular synthetic 3′ UTR, a broad cooperative alliance of AGO1–3 and LIM domain proteins is necessary for silencing and that short-term loss of any component by siRNA knockdown is highly unfavorable.

Post-transcriptional gene silencing involving miRNAs was first discovered over 20 years ago but only now are its precise mechanism and place within the array of cellular signaling networks becoming more clearly defined. There is now intense interest in how post-translational modification of components of miRNA biogenesis and miRISC can regulate and fine-tune both siRNA and miRNA-mediated gene silencing. A particularly active topic in the field is how phosphorylation of miRISC proteins can affect miRNA loading and recruitment to miRISC, ultimately affecting functionality (Golden et al., 2017, Horman et al., 2013, Lopez-Orozco et al., 2015, McKenzie et al., 2016, Rüdel et al., 2011, Shen et al., 2013, Warner et al., 2016, Yang et al., 2014, Zeng et al., 2008). Our findings identify that phosphorylation causes AGO selection for silencing by recruitment of a LIMD1 family member and sequential assembly of AGO-TNRC6A miRISC.

## Experimental Procedures

### Cell Culture

HEK293T, HeLa, and U2OS cells were routinely cultured in DMEM (Sigma) supplemented with 10% fetal calf serum (FCS), 100 U/mL penicillin, and 100 μg/mL streptomycin. Akt inhibitor MK-2206 was used at a final concentration of 10 μM in complete DMEM, for 16 hr.

### Plasmids

The generation of constructs used in this study are detailed in the Supplemental Experimental Procedures.

### Luciferase Reporter Assays

Firefly and Renilla luciferase activities were assayed with the Dual-Luciferase Reporter Assay System (Promega) according to the manufacturer’s instructions. Further details are provided in the Supplemental Experimental Procedures.

### Immunoprecipitation and Western Blotting

Routine methods to immunoprecipitate proteins were employed and detailed in the Supplemental Experimental Procedures. Antibodies used in the study are detailed in Table S2.

### Expression of Recombinant Proteins and Purification

Details are provided in Supplemental Experimental Procedures.

### MBP Direct Binding Assay

Details are provided in Supplemental Experimental Procedures.

### GST-Pull-Down Assays

Details are provided in Supplemental Experimental Procedures.

### Real-Time qPCR

Details are provided in Supplemental Experimental Procedures.

### Lentiviral Line Generation

To obtain pseudotyped lentivirus (recombinant HIV-1 with vesicular stomatitis virus G [VSV-G] envelope protein), we used the gene delivery and production system developed by Naldini et al. (1996). Details for selection are in the Supplemental Experimental Procedures.

### Protein Mass Spectrometry

Details are provided in Supplemental Experimental Procedures.

### IF Microscopy

For analysis of miRISC protein localization, cells were fixed with 4% paraformaldehyde and stained according to standard procedures, which are detailed in the Supplemental Experimental Procedures.

### PLA

PLAs were performed on HeLa cells prepared as for IF assays, including primary antibody incubation. Subsequently, Duolink In Situ PLA probes (1:10 in 2% BSA/0.025% Tween PBS) and orange detection reagents were used in accordance with the manufacturer’s instructions. For negative controls, each of the primary antibodies was added with an immunoglobulin G (IgG) antibody for the corresponding antibody species (rabbit, mouse, or goat, as appropriate). PLA signal in images has been digitally intensified for visual clarity in printed figures.

### CRISPR-Cas9 Cell Line Generation

*LIMD1* knockout CRISPR cell lines (HeLa) were generated using the lentiCRISPR v2 plasmid (Sanjana et al., 2014), acquired from Addgene (plasmid 52961). Further details for guide RNA sequence and clonal selection are in the Supplemental Experimental Procedures.

### Quantification and Statistical Analysis

PLA interaction events were counted using the ImageJ “Analyze Particles” function. Data were stratified according to quartiles for the control condition and displayed as a stacked histogram. Statistical significance was calculated using chi-square analysis (for grouped data).

Quantification of western blots was performed by densitometric analysis using ImageJ software. For quantification of relative binding in co-immunoprecipitation assays, co-immunoprecipitated proteins were double normalized first against their input levels and second against levels of immunoprecipitated protein species. Binding data displayed are relative to WT or untreated conditions.

Statistical significance was calculated using the Student’s t test, unless otherwise specified. Significance is represented as ^∗^p < 0.05, ^∗∗^p < 0.001, or ^∗∗∗^p < 0.0001 throughout.

## Author Contributions

K.S.B., K.M.S., Y.L., S.C.K.W., D.E.F., D.C.M., M.R.H., M.J.P., D.L., and T.V.S. designed and performed experiments and analyzed the data. K.M.D., J.G.F., P.R.G., K.Y., R.R., P.S.R., X.W., A.A.A., and M.J.P. provided reagents, experimental advice, and design. All authors contributed to editing and proofreading the manuscript. K.S.B., K.M.S., M.J.P., D.L., and T.V.S. wrote the manuscript. T.V.S. supervised and managed all research.

## Supplemental Information

Document S1. Supplemental Experimental Procedures, Figures S1–S7, and Table S2

Table S1. Luciferase Assay Data

Document S2. Article plus Supplemental Information
