# Supplementary material for: Argonaute Utilization for miRNA Silencing Is Determined by Phosphorylation-Dependent Recruitment of LIM-Domain-Containing Proteins
Source: Cell Rep. 2017 Jul 5;20(1):173–87. doi: 10.1016/j.celrep.2017.06.027 (PMC5507773; doi:10.1016/j.celrep.2017.06.027)
Supplement: Document S1. Supplemental Experimental Procedures, Figures S1–S7, and Table S2 [file mmc1.pdf]

Cell Reports, Volume *20*

Supplemental Information

Argonaute Utilization for miRNA Silencing Is Determined by Phosphorylation-Dependent Recruitment of LIM-Domain-Containing Proteins

Katherine S. Bridge, Kunal M. Shah, Yigen Li, Daniel E. Foxler, Sybil C.K. Wong, Duncan C. Miller, Kathryn M. Davidson, John G. Foster, Ruth Rose, Michael R. Hodgkinson, Paulo S. Ribeiro, A. Aziz Aboobaker, Kenta Yashiro, Xiaozhong Wang, Paul R. Graves, Michael J. Plevin, Dimitris Lagos, and Tyson V. Sharp

Figure S1.

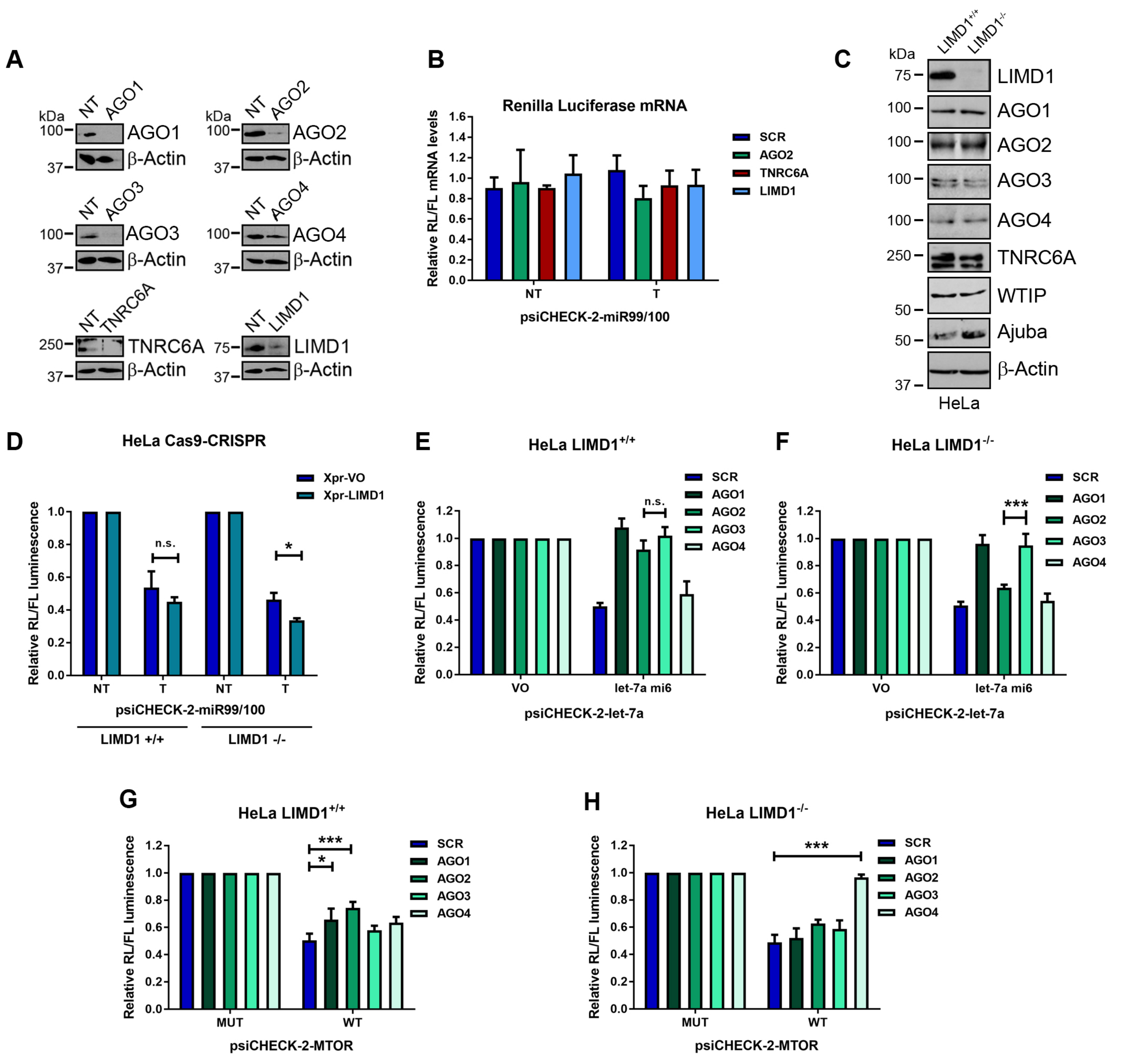

Figure S1. Related to Figure 1. Genetic ablation of LIMD1 switches AGO dependency within miRNA silencing. **(A)** Western blot analysis of siRNA-mediated knockdown of the indicated proteins of interest in HeLa cells. β-Actin loading control. **(B)** Quantitative real-time PCR (qRT-PCR) of Renilla Luciferase (RL) mRNA levels, normalised to Firefly Luciferase (FL) mRNA in HeLa cells. **(C)** Western blot analysis of the indicated proteins in CRISPR-Cas9 gene-edited HeLa cells; LIMD1 +/+ (Cas9 alone control) or LIMD1 -/-. **(D)** psiCHECK-2-miR-99/100 reporter assay in CRISPR-Cas9 gene-edited HeLa transfected with Xpress (Xpr)-tagged vector only (VO) or LIMD1. **(E)** psiCHECK-2-*let-7a* (non-targeting [NT], targeting [T]) reporter in CRISPR-Cas9 gene-edited HeLa cells with Cas9 alone control ($LIMD1^{+/+}$) or **(F)** LIMD1 knockout ($LIMD1^{-/-}$) cells, treated with the indicated siRNAs. **(G)** psiCHECK-2-MTOR (endogenous mutant [MUT] or wild type [WT] 3'-UTR) reporter in CRISPR-Cas9 gene-edited HeLa cells with Cas9 alone control ($LIMD1^{+/+}$) or **(H)** LIMD1 knockout ($LIMD1^{-/-}$) cells, treated with the indicated siRNAs. Unless otherwise stated, data shown are mean ±SEM, n=3,* $p<0.05$, ** $p<0.001$, *** $p<0.0001$.

Figure S2.

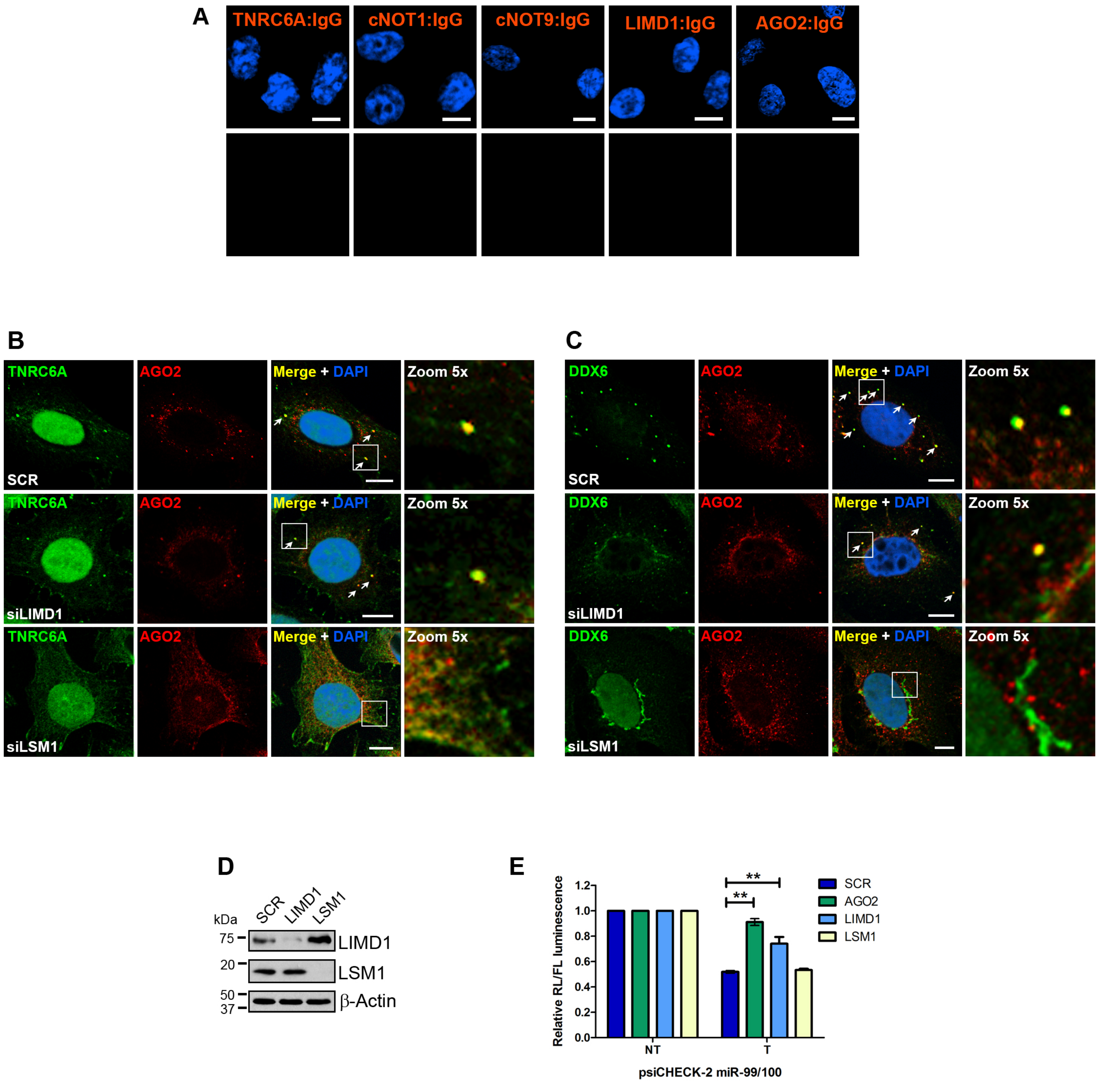

Figure S2. Related to Figure 2. Loss of P-body component LSM1 does not affect AGO2 interaction with TNRC6A/DDX6 or miRNA silencing activity.
(A) Negative control for endogenous *in situ* interaction determined by proximity ligation assay (PLA) of the indicated proteins. Cells stained with DAPI (top). **(B)** Endogenous immunofluorescence analysis of AGO2 with TNRC6A or **(C)** DDX6 in HeLa cells treated with the indicated siRNAs (SCR, non-targeting control). **(D)** Western blot analysis of HeLa cells in (B) and (C). **(E)** psiCHECK-2-miR-99/100 (NT [non-targeting], T [targeting]) reporter assay in HeLa cells treated with the indicated siRNAs. Data shown are mean ±SEM, n=3,* p<0.05, ** p<0.001, *** p<0.0001. Scale bars, 10 μm.

Figure S3.

A

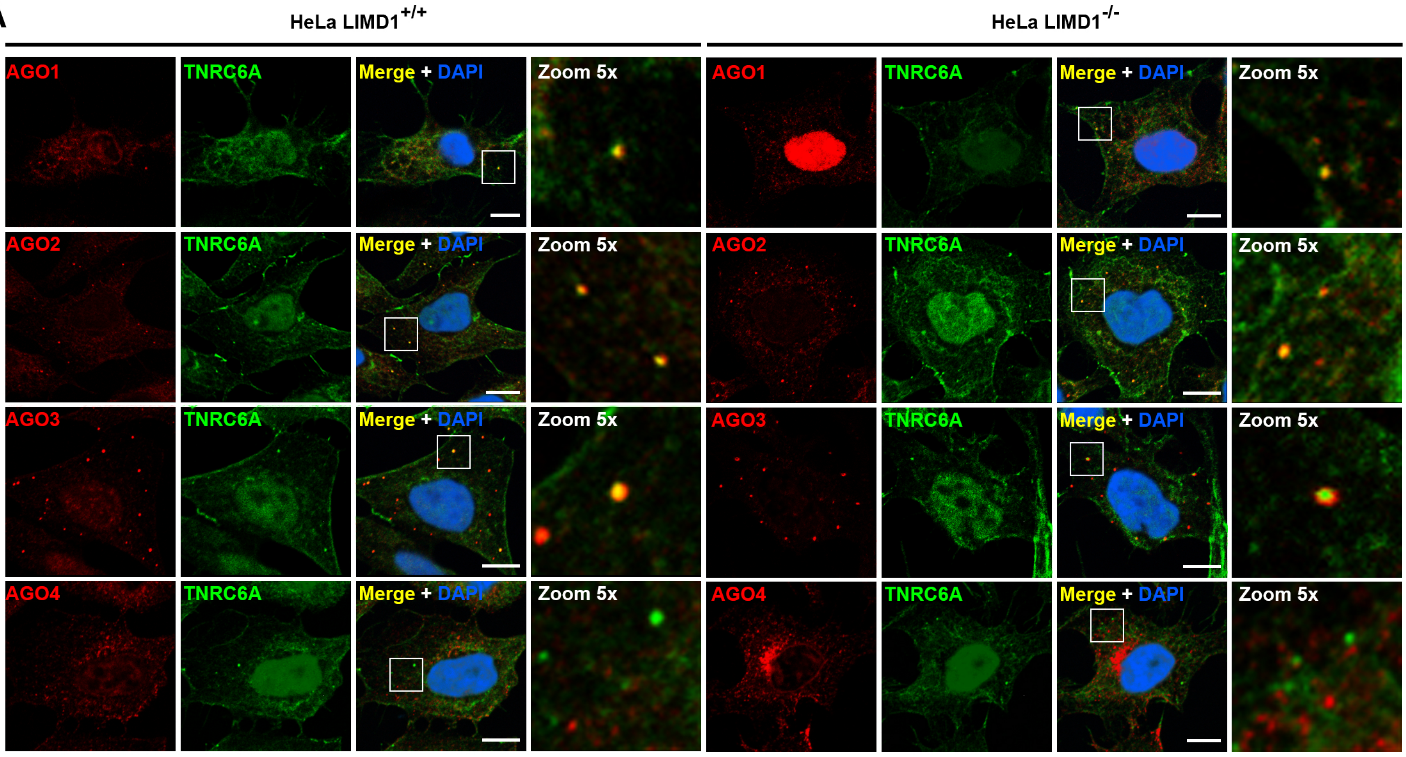

B

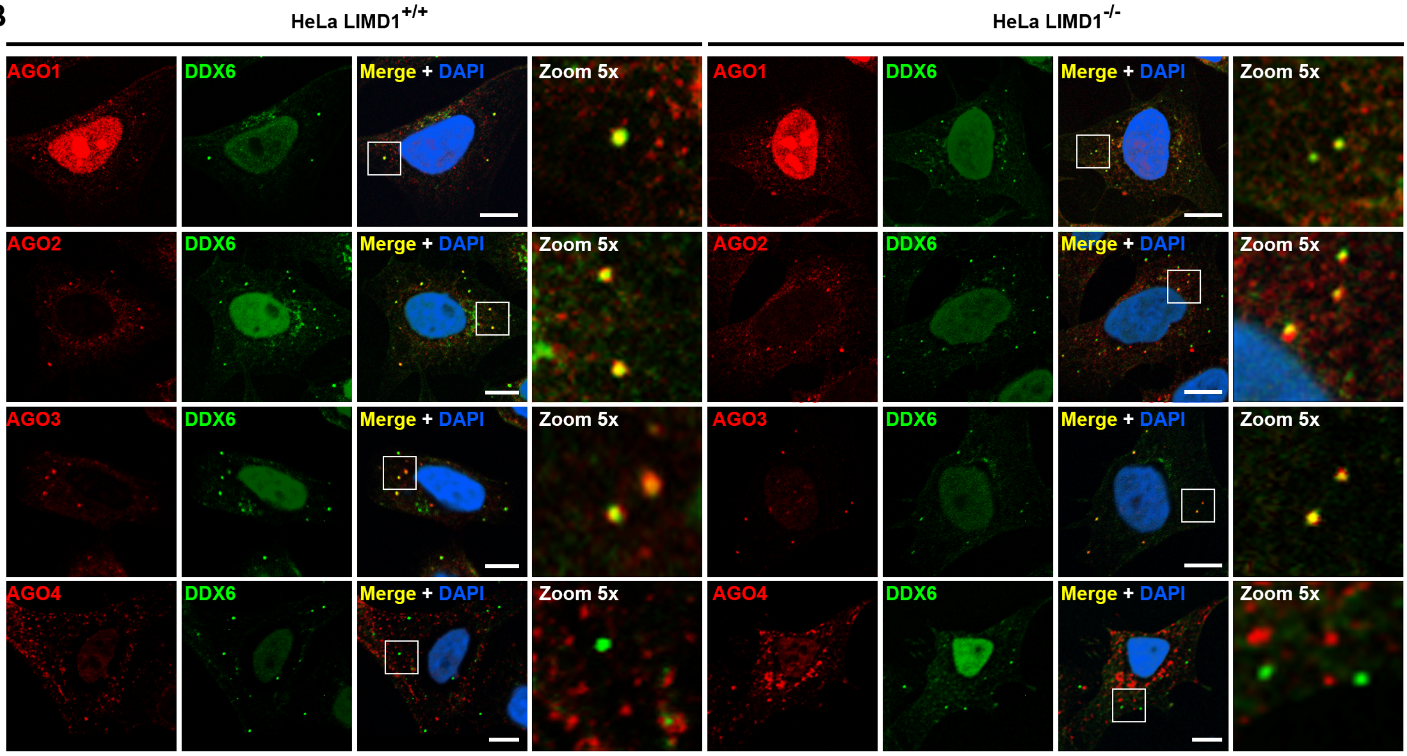

Figure S3. Related to Figure 2. Immunofluorescent colocalization of miRISC components is not a definitive indication of direct interactions.

(A) Endogenous immunofluorescence analysis in HeLa CRISPR LIMD1$^{+/+}$ and LIMD1$^{-/-}$ cells of AGO1, 2, 3 and 4 with TNRC6A and **(B)** DDX6. Scale bars, 10μm.

Figure S4.

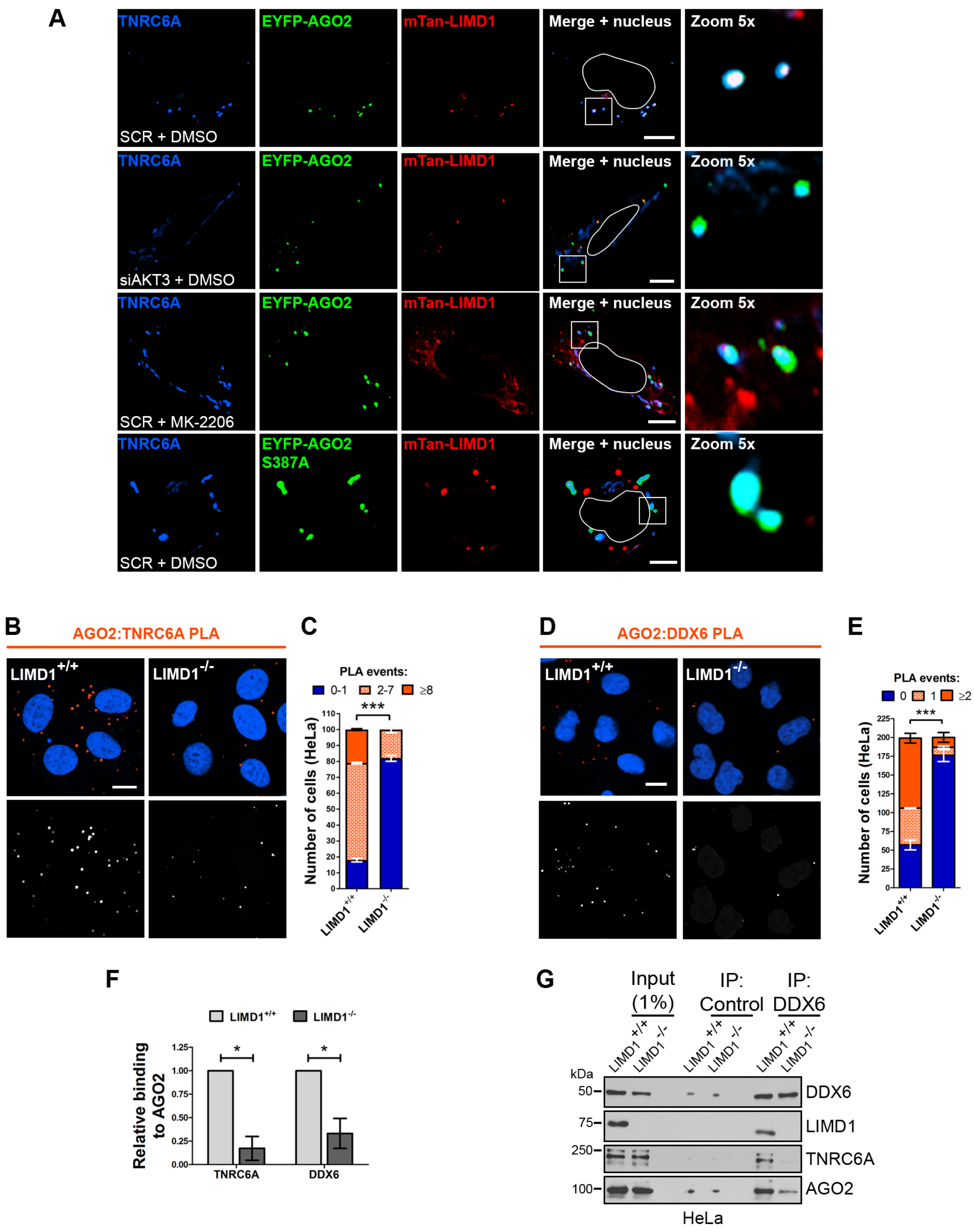

Figure S4. Related to Figure 2. Loss of LIMD1 significantly impairs AGO2 interaction with miRISC components TNRC6A and DDX6

A) Immunofluorescence of endogenous TNRC6A with EYFP-AGO2 or -AGO2 S387A and mTan-LIMD1. Cells treated with the indicated siRNAs (SCR, non-targeting control) or pan Akt-inhibitor (MK-2206). Scale bars, 10µm. **(B)** PLA analysis of endogenous AGO2 and TNRC6A interaction in CRISPR-Cas9 gene-edited HeLa cells. PLA signal orange, cells stained with DAPI (top); PLA signal white for visual clarity (bottom). **(C)** Quantification of PLA interaction events in (B), displayed as a stacked histogram. Data shown is mean ±SEM, n=3, total of 100 cells. ** $p<0.001$, *** $p<0.0001$, determined using the Chi-squared test. Scale bars, 10µm. **(D)** PLA analysis of AGO2:DDX6 interaction in above CRISPR-Cas9 gene-edited HeLa cell lines. **(E)** Quantification of (D) as in (C). **(F)** Quantification of relative binding of TNRC6A and DDX6 to AGO2 from CRISPR-Cas9 gene-edited HeLa LIMD1$^{+/+}$ or LIMD1$^{-/-}$ cells. Data shown is mean ±SEM, n=3, * $p<0.05$, determined using the Student's t-test **(G)** Immunoprecipiration of DDX6 from HeLa LIMD1$^{+/+}$ or LIMD1$^{-/-}$, analysed for the indicated proteins by western blot.

Figure S5.

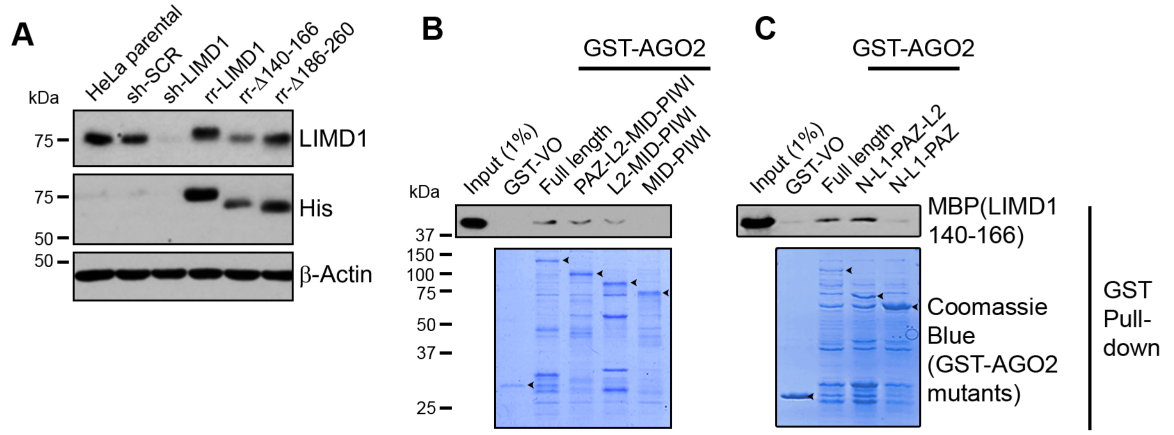

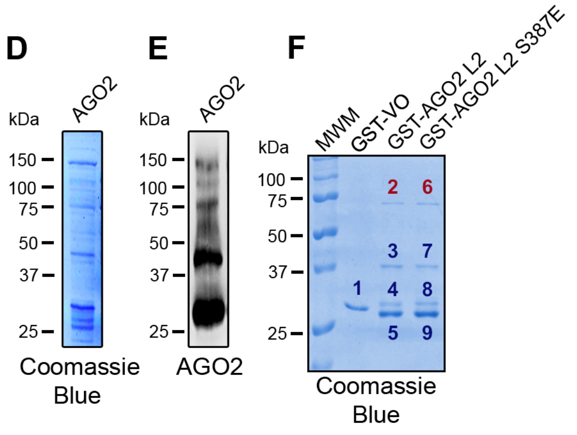

G

Band	Protein ID	# peptides	Score	Peptide sequences
1	GST	6	563	LTQSMAIIR<br>AEISMLEGAVLDIR<br>ERAEISMLEGAVLDIR<br>LLLEYLEEKYEEHLYER<br>YIAWPLQGWQATFGGGDHPPK<br>KFELGLEFPNLPYYIDGDVK
2	*E. Coli* Chaperone protein DNA K	5	626	TAEDYLGEPVTEAVITVPAYFNDAQR<br>AKLESLVEDLVNR<br>VALQDAGLSVSDIDDVILVGGQTR<br>HSQVFSTAEDNQSAVTIHVLQGER<br>SLGQFNLDGINPAPR
3	GST	4	427	AEISMLEGAVLDIR<br>ERAEISMLEGAVLDIR<br>LLLEYLEEKYEEHLYER<br>YIAWPLQGWQATFGGGDHPPK
	AGO2	3	289	SASFNTDPYVR<br>VLQPPSILYGGR<br>AIATPVQGVWDMR
4	GST	6	624	YEEHLYER<br>AEISMLEGAVLDIR<br>ERAEISMLEGAVLDIR<br>LLLEYLEEKYEEHLYER<br>YIAWPLQGWQATFGGGDHPPK<br>KFELGLEFPNLPYYIDGDVK
5	GST	6	565	LTQSMAIIR<br>AEISMLEGAVLDIR<br>ERAEISMLEGAVLDIR<br>LLLEYLEEKYEEHLYER<br>YIAWPLQGWQATFGGGDHPPK<br>KFELGLEFPNLPYYIDGDVK
6	*E. Coli* Chaperone protein DNA K	9	928	TAEDYLGEPVTEAVITVPAYFNDAQR<br>LINYLVEEFKK<br>AKLESLVEDLVNR<br>LESLVEDLVNR<br>VALQDAGLSVSDIDDVILVGGQTR<br>HSQVFSTAEDNQSAVTIHVLQGER<br>SLGQFNLDGINPAPR<br>KFEELVQTR<br>NQGDHLLHSTR
7	GST	5	477	YEEHLYER<br>AEISMLEGAVLDIR<br>ERAEISMLEGAVLDIR<br>LLLEYLEEKYEEHLYER<br>YIAWPLQGWQATFGGGDHPPK
	AGO2	2	180	VLQPPSILYGGR<br>AIATPVQGVWDMR
8	GST	6	543	LTQSMAIIR<br>AEISMLEGAVLDIR<br>ERAEISMLEGAVLDIR<br>LLLEYLEEKYEEHLYER<br>YIAWPLQGWQATFGGGDHPPK<br>KFELGLEFPNLPYYIDGDVK
9	GST	5	579	AEISMLEGAVLDIR<br>ERAEISMLEGAVLDIR<br>LLLEYLEEKYEEHLYER<br>YIAWPLQGWQATFGGGDHPPK<br>KFELGLEFPNLPYYIDGDVK

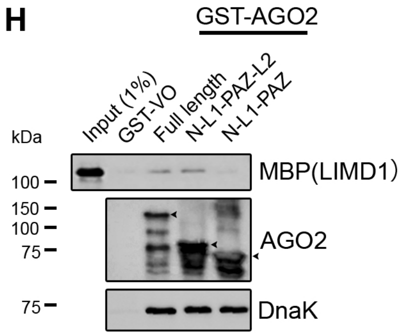

Figure S5. Related to Figure 3. LIMD1 AB-motif (a.a. 140-166) directly binds AGO2 L2 domain.
(A) sh-RNA mediated knock-down and rescue of RNAi resistant (rr) LIMD1 or deletion mutants, HeLa cell lines. sh-SCR is non-targeting control. **(B)** Direct binding assay of N-terminal and **(C)** C-terminal truncation mutants of GST-AGO2 with MBP-LIMD1 140-166 (ABD). **(D)** Purified GST-AGO2 analysed by Coomassie Blue staining and **(E)** western blot analysis for AGO2. **(F)** Degradation products of GST-AGO2 L2/ L2 S387E were identified by **(G)** mass spectrometry analysis. **(H)** DnaK identified as present in purified GST-AGO2 samples did not affect binding to LIMD1.

Figure S6.

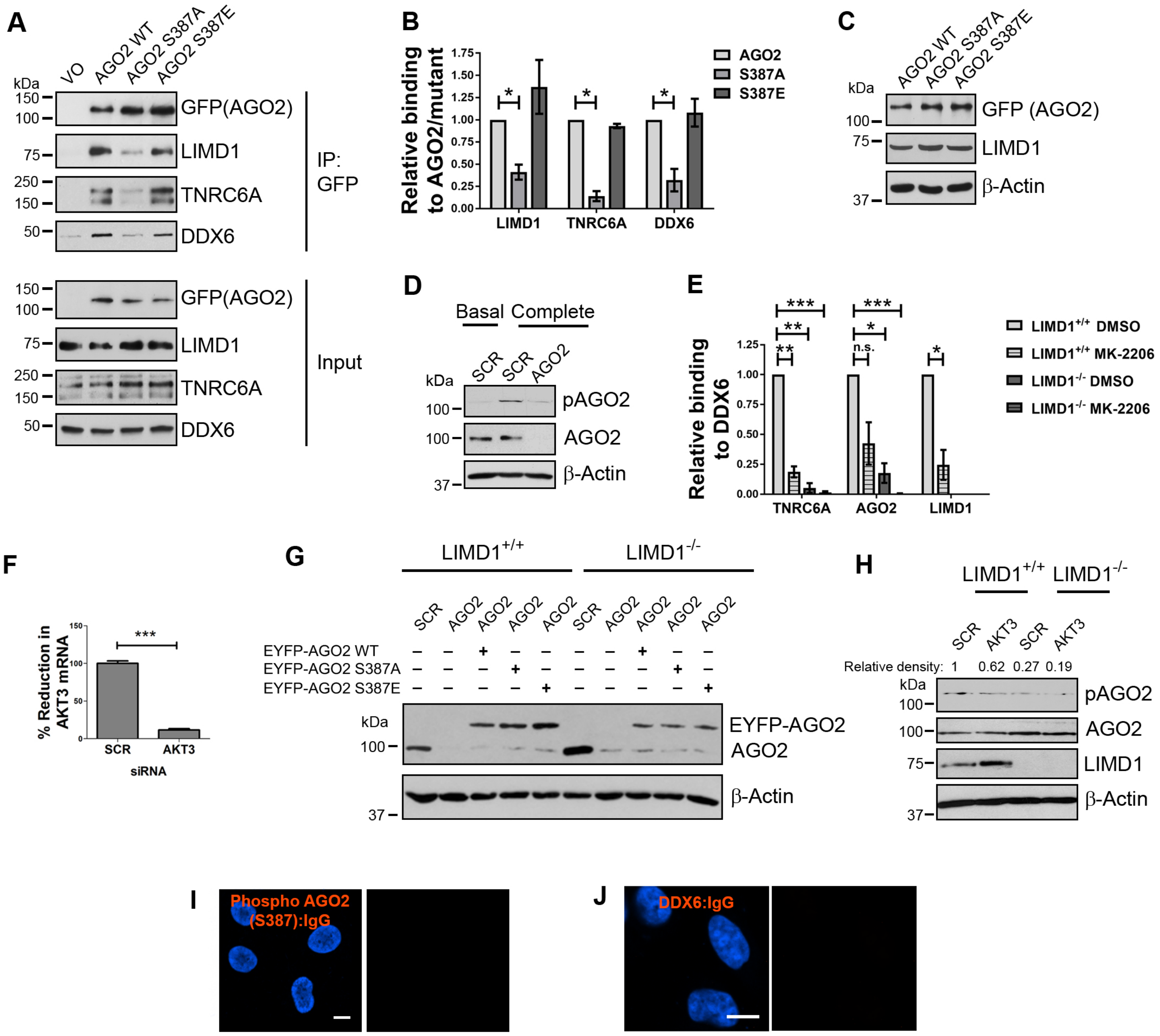

Figure S6. Related to Figures 4 and 5. Akt3-mediated phosphorylation of AGO2 S387 directs interaction with LIMD1 and recruitment of miRISC components TNRC6A and DDX6.

(A) Immunoprecipiration of GFP-VO, AGO2 and S387A/S387E point mutants from HEK293T lysate co-transfected with Xpr-LIMD1, analysed for the indicated proteins by western blot. **(B)** Quantification of co-IP in (A), data shown is mean ±SEM, n=4. **(C)** Western blot analysis of indicated overexpressed proteins in U2OS cells used for immunofluorescence analysis. β-Actin loading control. **(D)** Western blot analysis of phospho-AGO2 (S387) antibody specificity, in serum starved (basal) and complete medium conditions, treated with the indicated siRNAs. **(E)** Quantification of TNRC6A, AGO2 and LIMD1 interaction with DDX6 immunoprecipitated from HeLa LIMD1$^{+/+}$ or LIMD1$^{-/-}$ cells treated with DMSO or MK-2206. **(F)** qRT-PCR analysis of AKT3 mRNA in U2OS upon treatment with non-targeting (SCR) or AKT3 siRNA. **(G)** Western blot analysis of Cas9 control (LIMD1$^{+/+}$) and LIMD1$^{-/-}$ HeLa cell lines transfected with the indicated siRNAs and EYFP-VO/AGO2 constructs. AGO2 antibody detects both endogenous and EYFP-tagged AGO2. β-Actin loading control. **(H)** Western blot analysis of above HeLa CRISPR-Cas9 cell lines transfected with non-targeting (SCR) or AKT3 siRNA. Density of phospho-AGO2 (S387) band is calculated relative to SCR in the LIMD1$^{+/+}$ line. **(I)** Negative control PLA for the Phospho-S387-Ago2 antibody performed with goat IgG. PLA signal orange, cells stained with DAPI (top); PLA signal white for visual clarity (bottom). **(J)** Negative control PLA for the DDX6 antibody performed with rabbit IgG. Unless otherwise stated, data shown is mean ±SEM, n=3, * $p<0.05$, ** $p<0.01$, *** $p<0.001$, n.s. = not significant. Scale bars, 10μm.

Figure S7.

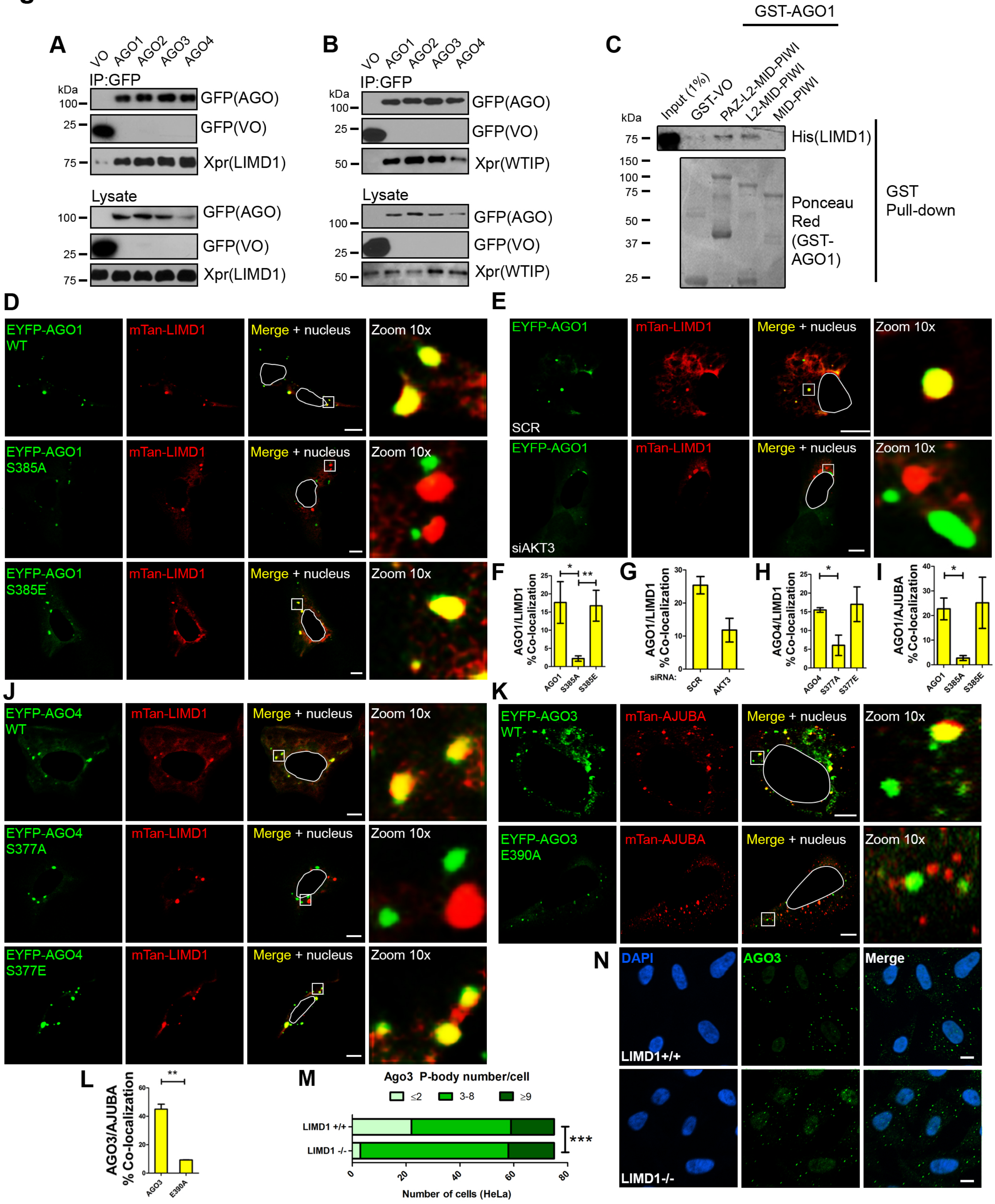

Figure S7. Related to Figures 6 and 7. AGO1-4 interact with LIMD1/Ajuba/WTIP in a phospho-dependent mechanism.

(A) Xpress-tagged (Xpr) LIMD1 co-immunoprecipitates with GFP-AGO1-4 from HEK293T lysate. **(B)** Xpr-WTIP co-IPs with GFP-AGO1-3 from HEK293T lysate. **(C)** The L2 domain of purified GST-AGO1 is required to directly bind His-LIMD1. **(D)** Co-localization of EYFP-AGO1/S385 point mutants with mTan-LIMD1 in U2OS cells. Nucleus outline in white. **(E)** Co-expression of EYFP-AGO1 with mTan-LIMD1 in U20S treated with non-targeting (SCR) or AKT3 siRNA. **(F)** Quantification of (D). **(G)** Quantification of (E). **(H)** Quantification of (J). **(I)** Quantification of Ago1/point mutant colocalization with AJUBA (images not shown). **(J)** Co-localization of EYFP-AGO4/S377 point mutants with mTan-LIMD1 in U2OS cells. **(K)** Co-expression of mTan-AJUBA with EYFP-AGO3 WT/E390 point mutant. **(L)** Quantification of (K). **(M)** Stacked histogram quantification of number of AGO3 P-bodies in (N). **(N)** Immunofluorescence staining for endogenous AGO3 in HeLa CRISPR-Cas9 cell lines. DAPI, nuclear staining. Unless otherwise stated, scale bars, 10µm. p<0.001, *** p<0.0001.

Supplemental Experimental Procedures

Luciferase Reporter Assays

For siRNA knockdown experiments, cells were reverse transfected in 96 well plates with siRNA at 40 nM using INTERFERin reagent (Polyplus transfection Illkirch, France). After 48h cells were transfected with 20 ng/well of psiCheck2 plasmid using Fugene 6 reagent or Viafect reagent (Promega). After a further 24h cells were harvested in 1x Passive Lysis buffer. Lysates were assayed for Firefly and Renilla luciferase activities with the Dual-Luciferase Reporter Assay System (Promega) according to the manufacturer's instructions on the Wallac Victor2 1420 multilabel counter (Perkin-Elmer, Waltham, MA, USA).

For reporter assays without prior siRNA treatment, cells were plated in 96 well plates and 24h later transfected with the reporter plasmids as above. After 24h cells were harvested in 1x Passive Lysis buffer and assayed as above.

For reporter assays that included co-transfection of miRNA mimic, cells were transfected with reporter plasmids and miRNA mimic at 20 nM using JetPrime Reagent (Polyplus) and harvested 24h later in 1X Passive Lysis buffer.

Plasmids

Annealed oligonucleotides containing five tandem miR-99/100 sites with seed matches (T) or seed mismatches (NT) were cloned into the 3' UTR region of the Renilla luciferase cassette in psiCheck2 (Promega, WI Madison USA) using the XhoI and PmeI restriction sites. Matched sites contained the sequence 5'AGCAAGTGTAACGG**TACGGGT**A-3' (seed sequence in bold). Mismatched sites contained the sequence 5'AGCAAGTGTAACGG**TAATAAC**A-3'. For the artificial let-7a reporter six seed-matched let-7a sites were cloned into the XhoI and NotI sites of psiCheck2 5'-AACTATACAACGT**CTACCTC**A-3' (seed sequence in bold). For FGFR3 and MTOR 3'UTR reporters, the 3'UTRs were PCR amplified using Phusion PCR mastermix using human genomic DNA as a template and primers containing XhoI and PmeI sites. PCR products were cloned into XhoI and PmeI sites of psiCheck2. miR-99/100 site mutants were

generated by site-directed mutagenesis using the Q5 site directed mutagenesis kit (New England Biolabs), according to the manufacturer's instructions. psiCheck2-TGFBR2 3'UTR WT and mutant plasmids were obtained from Addgene (31882 and 31883)

pFLRu *h*LIMD1 shRNA rrLIMD1-FH construct has previously been described (Foxler et al., 2012). Deletion mutants for the rrLIMD1 were made by site directed mutagenesis.

Ligation-independent cloning was carried out as described by Eschenfeldt *et al* (Eschenfeldt et al., 2009). Briefly, PCR products were separated on 1% agarose gels and purified. pMCSG7 or pMCSG10 vectors were linearized by digestion with SspI, and pETFPP_2 by BseRI (for cloning of MBP-LIMD1). Both PCR products and vectors were then treated with T4 DNA polymerase in the presence of the specific single deoxynucleotide. The transformation was followed by the usual protocol. Positive clones were identified with colony PCR, using T7 promoter and T7 terminator primers. Positive colonies were grown in 5 ml of LB media overnight and plasmids were prepared using a Plasmid Miniprep Kit (Roche).

Plasmids expressing mTan-LIMD1, mTan-WTIP and mTan-Ajuba have been described previously (James et al., 2010). pEYFP-C1-Ago1 and Ago2 were obtained from Dr. Tariq Rana (Chu and Rana, 2006) . Ago3 and Ago4 cDNAs were cloned into the EcoRI and XbaI sites of pEYFP-C1.

Expression of recombinant proteins and purification

Recombinant plasmids containing the target clones were transformed into Rosetta (DE3) expression strain. Single colonies from each plate were transferred into 5 ml of LB and incubated at 37°C, 250 rpm for 2.5 h until the optical density at 600 nm reached 0.6-0.8. IPTG was then added to reach a final concentration of 1 mmol/L to induce the protein expression at 25°C overnight. After overnight expression, the bacteria were collected and frozen on dry ice. The pellets were then thawed on ice and re-suspended in RIPA buffer, followed by sonication.

The supernatant was collected after centrifugation at 4°C for 15 min at 21,500 g and stored at -80°C for later use. For MBP-tagged proteins, a small amount of supernatant was incubated with 25 µl of His-Select Nickel Affinity Gel (SIGMA, P6611) and incubated at 4°C rotating for 30 min. The resin was then washed three times with His binding buffer (50 mM Tris-HCL, pH 8.0; 500 mM NaCl; 5 mM imidazole), followed by eluting in elution buffer (50 mM Tris-HCL, pH 8.0; 500 mM NaCl; 50 mM imidazole) by rotating at 4°C overnight. After overnight incubation, the supernatant was collected after centrifugation at 4°C for 1 min at 500 g and the imidazole was reduced by Amicon Ultra (Amicon, UFC501024). The purified lysates was then stored at -20°C for later use.

MBP direct binding assay

10ul of anti-MBP magnetic beads was washed once in PBS and incubated with cell lysates at 4 °C with rotation for 30 min to conjugate the MBP-tagged proteins onto the beads. The beads was then washed three times with 200µl RIPA with vortex and applied to magnet for 30s to remove the supernatant, followed by incubating with crystallography grade un-tagged AGO2 at 4 °C with rotation for 2h. After incubation, the beads were washed three times with 500µl binding buffer with vortex and the supernatant was removed. Proteins were finally eluted in 20µl 2xSDS-PAGE sample loading buffer and then analysed by Western Blot.

GST-pulldown assays

20µl of pre-blocked Glutathione Sepharose 4B resin (GE Healthcare, 17-0756-01) was blocked at 4°C with rotation for overnight. Bacterial cell lysates were incubated with the resin at 4°C with rotation for 30min. The resin was then washed three times with 200µl RIPA with gentle vortex and centrifugation at 4 °C for 1 min, 500 g. The supernatant was carefully removed. The pre-purified MBP-tagged protein in binding buffer (150 mM NaCl, 20 mM Tris (pH 8.0), 1 mM $MgCl_2$, 0.1 % (v/v) IGEPAL CA-630 and 10 % (v/v) glycerol (Fisher Chemical, G/0650/17)) was added and incubated at 4 °C with rotation for 2 h. After incubation, the beads were washed three times with 500µl the binding buffer with vortex and centrifugation at 4 °C

for 1 min, 500 g to remove the supernatant. Proteins were finally eluted in 20µl 2xSDS-PAGE sample loading buffer and then analysed by Western Blot.

Lentiviral Line Generation

HeLa cells were transduced with viral supernatants and after three days, cells were subcultured into medium containing puromycin (1.0 µg/ml) to select for transduced cells. Medium containing puromycin was replaced every two to three days and following ten days of selection frozen stocks were made. Cells were maintained in medium containing puromycin, but when performing experiments with the lines, puromycin was removed. Western blotting was used to validate the lentiviral lines.

Immunofluorescence microscopy

For endogenous protein detection, following fixation cells were permeabilised with 0.3% Triton and blocked with 2% BSA/0.025% Tween. Primary antibodies were diluted in 2% BSA/0.025% Tween in PBS as follows: TNRC6A (1:300), AGO1 (1:400 Millipore) AGO2 (Rabbit 1:200, Goat 1:100, Mouse 1:400), AGO3 (1:400 Millipore), AGO4 (1:400 Millipore) LIMD1 (1:300), cNOT1 (1:100), cNOT9 (1:50), DDX6 (1:200), Phospho-AGO2 (1:100). Cells were washed in PBS and stained with Alexa-Fluor conjugated secondary antibodies (1:500 or 1:2000) (Life Technologies). Images were captured at x40 or x100 magnification with a Zeiss LSM 710 confocal microscope.

Immunoprecipitation

For immunoprecipitation of endogenous proteins, Dynabeads® Co-immunoprecipitation Kit including Dynabeads® M-270 Epoxy beads was used. 1.5mg of beads/IP were conjugated with 7.5µg/mg beads of immunoprecipitating antibody overnight at 37ºC with shaking. Cells were collected, centrifuged, and resuspended in P-body lysis buffer (Chu and Rana, 2006) supplemented with 'Complete' protease inhibitors (Roche) and PhosSTOP phosphatase inhibitors (Roche) at a 1:9 ratio of cell pellet weight (mg) to lysis buffer volume (µl). Lysates

were incubated on ice for 15 minutes and centrifuged at 2,600 rpm for 5 minutes. The cleared lysate was rotated at 4ºC for 30 minutes with the antibody-conjugated Dynabeads®, which were subsequently washed 4 times with unsupplemented P-body lysis buffer. Protein complexes were eluted in 2x SDS-PAGE sample buffer or 0.1M glycine pH 2.5 and analysed by western blot.

For immunoprecipitation of ectopically expressed proteins, transfected HEK293T cells were lysed by the addition of ice-cold RIPA buffer (150mM NaCl, 1%(v/v) IGEPAL-630, 0.5%(w/v) sodium deoxycholate, 0.1%(w/v) SDS, 50mM Tris, pH 8) supplemented with 'Complete' protease inhibitors (Roche) and PhosSTOP phosphatase inhibitors (Roche) and scraped using a cell scraper. Lysates were rotated at 4°C for 20min and centrifuged at 14,800 rpm for 10min to pellet debris. Protein concentration of cleared lysate was determined by Bradford assay and 500 µg of protein was used for IP. The cleared lysate was rotated at 4°C for 4h with immunoprecipitation matrix (Santa Cruz sc-45042) previously conjugated to 2µg of immunoprecipitating antibody (2% BSA/PBS, 4ºC for 2 hours). The immunoprecipitation matrix–antibody complex was then washed three times with ice-cold RIPA, and protein complexes were eluted in 5×SDS–PAGE sample buffer and analysed by Western blot.

Protein Mass Spectrometry

Protein bands were excised from the gel, destained, reduced, alkylated and digested with trypsin following standard protocols. The resulting peptide solutions were desalted using U-C18 ZipTips (Millipore) following the manufacturer's instructions. Samples were spotted directly onto the MALDI target plate in addition to matrix solution (α-Cyano-4-hydroxycinnamic acid).

MS/MS analysis was performed on a Bruker Ultraflex in positive ion, reflector mode. MS spectra were acquired in the mass range m/z 800-4000. The 10 most intense peaks in the MS spectra for each sample were then selected for MS/MS analysis. The MS/MS datawere submitted to database searching against NCBInr, using a locally running copy of the Mascot

software (Matrix Science) through a Biotools (Bruker Daltonics) interface. Scores greater than 62 indicate identity or extensive homology ($p<0.05$).

Real-time quantitative PCR

RNA was extracted from cells using TriReagent (Sigma) or Reliaprep RNA miniprep (Promega) according to manufacturers' instructions. Following treatment with DNase I, RNA was diluted and added to GoTaq 1-step RT-qPCR (Promega) reactions along with specific primers. RT-qPCR was performed on the Applied Biosystems 7500 Real-Time PCR System and relative amounts of targets were quantified using the $2^{-\Delta\Delta Ct}$ method.

CRISPR-Cas9 Cell Line Generation

The gRNA sequence (5'-GGTCTTCCAAGATCAAACTC) targets Exon 1 of LIMD1. Transduced HeLa cells were initially selected with puromycin (1 µg/ml) prior to screening of single cell colonies for LIMD1 knockout by Western blot. Genetic mutation was confirmed by Sanger sequencing of genomic DNA utilising the primers 5' GAGTAGAGGCCCTGTCAATGG and 5' CACAGATCCCAGGCTACCATC.
