# Supplementary material for: Argonaute Utilization for miRNA Silencing Is Determined by Phosphorylation-Dependent Recruitment of LIM-Domain-Containing Proteins
Source: Cell Rep. 2017 Jul 5;20(1):173–87. doi: 10.1016/j.celrep.2017.06.027 (PMC5507773; doi:10.1016/j.celrep.2017.06.027)
Supplement: Document S2. Article plus Supplemental Information [file mmc3.pdf]

Cell Reports

Article

Argonaute Utilization for miRNA Silencing Is Determined by Phosphorylation-Dependent Recruitment of LIM-Domain-Containing Proteins

Graphical Abstract

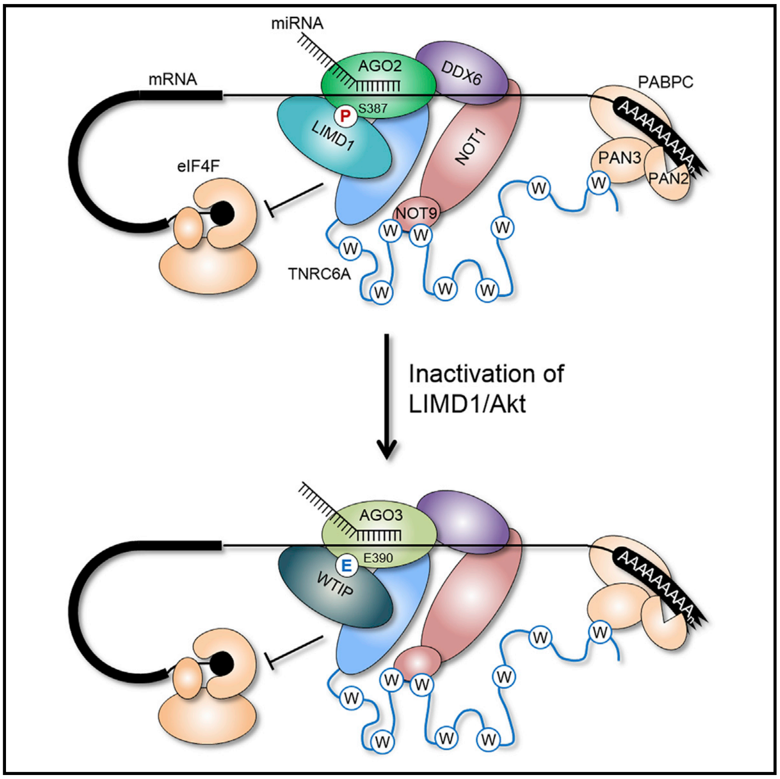

Highlights

- LIMD1 is necessary for the Akt3-dependent assembly of an AGO-TNRC6A functional miRISC
- AGO1, 2, and 4 recruit LIMD1 in a phospho-dependent manner
- AGO2 to AGO3 switching occurs upon LIMD1 ablation in HeLa cells
- AGO3-WTIP replaces AGO2-LIMD1 for miRNA silencing in an Akt3-independent manner

Authors

Katherine S. Bridge, Kunal M. Shah, Yigen Li, ..., Michael J. Plevin, Dimitris Lagos, Tyson V. Sharp

Correspondence

t.sharp@qmul.ac.uk

In Brief

Argonaute (AGO) proteins mediate post-transcriptional gene silencing through formation of the microRNA-induced silencing complex (miRISC). Bridge et al. identify LIM-domain-containing proteins as essential for miRISC formation through a phosphorylation-dependent mechanism. This is critical for post-transcriptional gene silencing and reveals that miRISC functionality is maintained by “AGO switching.”

Bridge et al., 2017, Cell Reports *20*, 173–187
July 5, 2017 © 2017 The Authors.
http://dx.doi.org/10.1016/j.celrep.2017.06.027

Cell Reports
Article

OPEN ACCESS
CellPress

Argonaute Utilization for miRNA Silencing Is Determined by Phosphorylation-Dependent Recruitment of LIM-Domain-Containing Proteins

Katherine S. Bridge,[1,9] Kunal M. Shah,[1,9] Yigen Li,[1,9] Daniel E. Foxler,[1] Sybil C.K. Wong,[1] Duncan C. Miller,[1] Kathryn M. Davidson,[1] John G. Foster,[1] Ruth Rose,[2] Michael R. Hodgkinson,[3] Paulo S. Ribeiro,[4] A. Aziz Aboobaker,[5] Kenta Yashiro,[6] Xiaozhong Wang,[7] Paul R. Graves,[6] Michael J. Plevin,[3] Dimitris Lagos,[8] and Tyson V. Sharp[1,10,*]
[1]Centre for Molecular Oncology, Barts Cancer Institute, Queen Mary University of London, John Vane Science Centre, Charterhouse Square, London EC1M 6BQ, UK
[2]School of Biological and Chemical Sciences, Queen Mary University of London, Fogg Building, Mile End Road, London E1 4NS, UK
[3]Department of Biology, University of York, Heslington, York YO10 5DD, UK
[4]Centre for Tumour Biology, Barts Cancer Institute, Queen Mary University of London, John Vane Science Centre, Charterhouse Square, London EC1M 6BQ, UK
[5]Department of Zoology, University of Oxford, The Tinbergen Building, South Parks Road, Oxford OX1 3PS, UK
[6]Department of Radiation Oncology, New York-Presbyterian Brooklyn Methodist Hospital, 506 6th Street, Brooklyn, NY 11215, USA
[7]Department of Biochemistry, Molecular Biology and Cell Biology, Northwestern University, Evanston, IL 60208, USA
[8]Centre for Immunology and Infection, Hull York Medical School and Department of Biology, University of York, Heslington, York YO10 5DD, UK
[9]These authors contributed equally
[10]Lead Contact
*Correspondence: t.sharp@qmul.ac.uk
http://dx.doi.org/10.1016/j.celrep.2017.06.027

SUMMARY

As core components of the microRNA-induced silencing complex (miRISC), Argonaute (AGO) proteins interact with TNRC6 proteins, recruiting other effectors of translational repression/mRNA destabilization. Here, we show that LIMD1 coordinates the assembly of an AGO-TNRC6 containing miRISC complex by binding both proteins simultaneously at distinct interfaces. Phosphorylation of AGO2 at Ser 387 by Akt3 induces LIMD1 binding, which in turn enables AGO2 to interact with TNRC6A and downstream effector DDX6. Conservation of this serine in AGO1 and 4 indicates this mechanism may be a fundamental requirement for AGO function and miRISC assembly. Upon CRISPR-Cas9-mediated knockout of LIMD1, AGO2 miRNA-silencing function is lost and miRNA silencing becomes dependent on a complex formed by AGO3 and the LIMD1 family member WTIP. The switch to AGO3 utilization occurs due to the presence of a glutamic acid residue (E390) on the interaction interface, which allows AGO3 to bind to LIMD1, AJUBA, and WTIP irrespective of Akt signaling.

INTRODUCTION

MicroRNAs are ~22 nucleotide non-coding RNA molecules that silence gene expression post-transcriptionally (Bartel, 2004). Their loading onto Argonaute (AGO) proteins facilitates base-pairing to mRNA targets with partial complementarity and ultimately translational repression and mRNA degradation (Huntzinger and Izaurralde, 2011; Hutvagner and Simard, 2008). Repression is executed by the microRNA-induced silencing complex (miRISC), a large multi-subunit complex assembled by AGO-mediated recruitment of GW182/TNRC6 proteins and downstream effector complexes (Behm-Ansmant et al., 2006; Chekulaeva et al., 2011; Lian et al., 2009; Liu et al., 2005). TNRC6 proteins facilitate translational repression, mRNA destabilization, and ultimately degradation of target mRNAs via recruitment of effector proteins to the miRISC, such as those involved in decapping (DCP1/2), RNA unwinding (DDX6), and deadenylation (CCR4-NOT) (Bazzini et al., 2012; Chen et al., 2014; Chu and Rana, 2006; Rouya et al., 2014).

Despite significant advances in our understanding of the terminal effects of miRNAs on their targets, the early steps of miRISC assembly and its functional activation remain poorly understood. Recent studies have explored the role of post-translational modification of AGO proteins (Jee and Lai, 2014; Rüdel et al., 2011). For example, phosphorylation of AGO2 Y529 inhibits loading of small RNAs (Rüdel et al., 2011), while EGFR-dependent phosphorylation of AGO2 Y393 prevents processing of looped precursor RNAs into mature miRNAs (Shen et al., 2013). Additionally, Akt3-mediated phosphorylation of AGO2 S387 reduces the mRNA cleavage activity of AGO2 and drives it toward translational repression associated with miRNA-mediated silencing (Horman et al., 2013; Zeng et al., 2008). It has also recently been shown that primary T cells display a signaling-dependent shift in miRISC configuration. While the majority of miRNAs were found in low-molecular-weight miRISC complexes in resting T cells, stimulation of phosphatidylinositol 3-kinase (PI3K) signaling caused an increased association of miRNAs with high-molecular-weight miRISC complexes and an enhancement of miRNA-mediated silencing (La Rocca et al., 2015). These data indicate that miRISC assembly into an active complex and the

This is an open access article under the CC BY license (http://creativecommons.org/licenses/by/4.0/).

protein-protein interactions therein are highly dynamic processes, controlled by signal transduction cascades.

To date, the four human AGO proteins (AGO1–4) have been demonstrated to function largely redundantly with regards to miRNA loading and target recognition, with the functionally dominant AGO currently attributed to the expression levels of these proteins (Dueck et al., 2012; Su et al., 2009; Wang et al., 2012). How these overlapping functions are facilitated in light of recent findings demonstrating the role of signaling pathways in the regulation of miRNA biogenesis and function is unclear. Interestingly, plasticity in AGO usage following genetic ablation has been observed (Wang et al., 2012), although the mechanism underpinning this switch remains unknown.

The discovery that AGO2 activity is regulated by phosphorylation, and indeed other post-translational modifications, raises the question as to whether all four human AGO proteins are subject to regulation by signaling, and whether, through differential signal transduction and phosphorylation, may not exhibit complete redundancy (Golden et al., 2017; Lopez-Orozco et al., 2015; Patranabis and Bhattacharyya, 2016; Sahin et al., 2014; Shen et al., 2013; Zeng et al., 2008). Horman and colleagues suggest that the AGO2 S387 phosphorylation diverts its activity from mRNA cleavage to (or toward) miRNA-mediated silencing (Horman et al., 2013). However, this study did not consider the high homology within the S387-containing L2 domain between AGO2 and other human AGO proteins, which are not involved in small interfering RNA (siRNA) silencing. We therefore sought to determine whether signaling regulates miRISC assembly and function across the entire AGO family and whether this regulation would identify an additional dimension of AGO family member functional specificity.

LIMD1 is a component of miRISC required for silencing (James et al., 2010). However, its precise function in miRISC and miRNA silencing is poorly understood. Here, we demonstrate that LIMD1 is crucial for AGO2 miRNA function and that loss of AGO2-LIMD1-mediated miRNA silencing reveals a previously unknown mechanism of AGO selection, with LIMD1 association being a key determinant of AGO utilization. LIMD1 is required for the recruitment of TNRC6A and downstream effectors (DDX6) to AGO2. We show that Akt3-mediated phosphorylation of AGO2 (S387) promotes a phospho-dependent interaction with LIMD1 that is vital for enabling engagement of AGO2 with TNRC6A, revealing the mechanism by which AGO-S387 phosphorylation regulates miRISC function. The phosphorylation-dependent interaction with LIMD1 also extends to the other human AGO family members, with a conserved serine also present in AGO1 and AGO4. AGO3 lacks this conserved serine residue but instead contains a phospho-mimic glutamic acid residue (E390), which facilitates signaling-independent binding to LIMD1 family members. Our findings demonstrate that loss of LIMD1 abolishes AGO2-mediated miRNA silencing and results in redistribution of AGO utilization for miRNA silencing to AGO3.

RESULTS

AGO Utilization Is Determined by LIMD1

Members of the LIMD1 family (LIMD1, AJUBA, and WTIP) of LIM-domain-containing proteins associate with miRISC and are required for miRNA-mediated silencing (James et al., 2010). To examine the role of LIMD1 on AGO2 function, we designed a Renilla luciferase reporter containing five targeted or non-targeted miR-99/100 binding sites in its 3′ UTR. We observed significant de-repression of the targeted reporter upon AGO2, TNRC6A, or LIMD1 siRNA-mediated knockdown in HeLa cells, whereas little or no de-repression was seen with depletion of AGO1, 3, or 4 (Figure 1A and S1A). The high specificity of this reporter for AGO2 is surprising as a previous study did not find evidence for miRNA sorting into AGO1, 2, or 3 in HeLa cells and concluded that association of a particular miRNA with a particular AGO solely reflected AGO protein abundance (Dueck et al., 2012). Moreover, mRNA levels of the targeted reporter were unchanged, indicating that, at the time points analyzed, the reporter is subject to miRNA-mediated translation inhibition rather than target cleavage (Figure S1B).

To investigate the mechanistic function of LIMD1 in miRNA silencing independently of engagement of the RNAi machinery, we genetically ablated *LIMD1* in HeLa cells using CRISPR-Cas9 technology (Figure S1C). Unexpectedly, we found that repression of the miR-99/100 reporter was equivalent in *LIMD1*$^{+/+}$ and *LIMD1*$^{-/-}$ cell lines (~50% repression of the targeted reporter), although expression of exogenous LIMD1 enhanced silencing in the *LIMD1*$^{-/-}$ line (Figure S1D). To explore the mechanism underlying the maintenance of silencing in the *LIMD1*$^{-/-}$ line, we analyzed reporter activity when this isogenic HeLa CRISPR pair was treated with siRNAs targeting AGO1–4 (Figures 1B and 1C). We observed a switch in the requirement for AGO proteins in this pair of HeLa lines. AGO2 depletion only led to de-repression of miRNA silencing in the *LIMD1*$^{+/+}$ line, whereas, in the *LIMD1*$^{-/-}$ line, AGO3 depletion produced a de-repression. As AGO2 comprises the majority (~60%) of the AGO pool in HeLa cells (Petri et al., 2011) and its levels remained unchanged upon LIMD1 loss (Figure S1C), the switch in AGO utilization may reflect the importance of LIMD1 specifically for AGO2 function in HeLa.

In order to interrogate the generality of this switch in AGO utilization, we employed a *let-7a*-targeted reporter. Similarly, we observed that loss of LIMD1 (*LIMD1*$^{-/-}$) rendered AGO2 unable to contribute to silencing, whereas in *LIMD1*$^{+/+}$ cells, de-repression was induced upon AGO2 knockdown (Figures S1E and S1F). In contrast to the miR-99/100 reporter, however, AGO1 and AGO3 knockdown resulted in de-repression of the *let-7a*-targeted reporter, regardless of *LIMD1* genotype, indicating this reporter and miRNA demonstrated a broader specificity for AGO and LIMD1 family proteins. To rule out the possibility that the observed switch in AGO use was due to the use of a synthetic 3′ UTR miRNA reporter, we also examined whether LIMD1 loss resulted in a switch in AGO requirement of two natural 3′ UTRs reporters—FGFR3 and MTOR, which each contain, in addition to other miRNA sites, single miR-99/100 sites (Figures 1H, 1I, S1G, and S1H). Both reporters showed a switch from AGO1/2 dependency, to AGO1/3 and AGO4, respectively, in the absence of LIMD1.

LIMD1 Is Critical for the Interaction of AGO2 with TNRC6A and DDX6

We next examined the binding partners and function of LIMD1 in miRISC in more detail to shed light on why loss of LIMD1

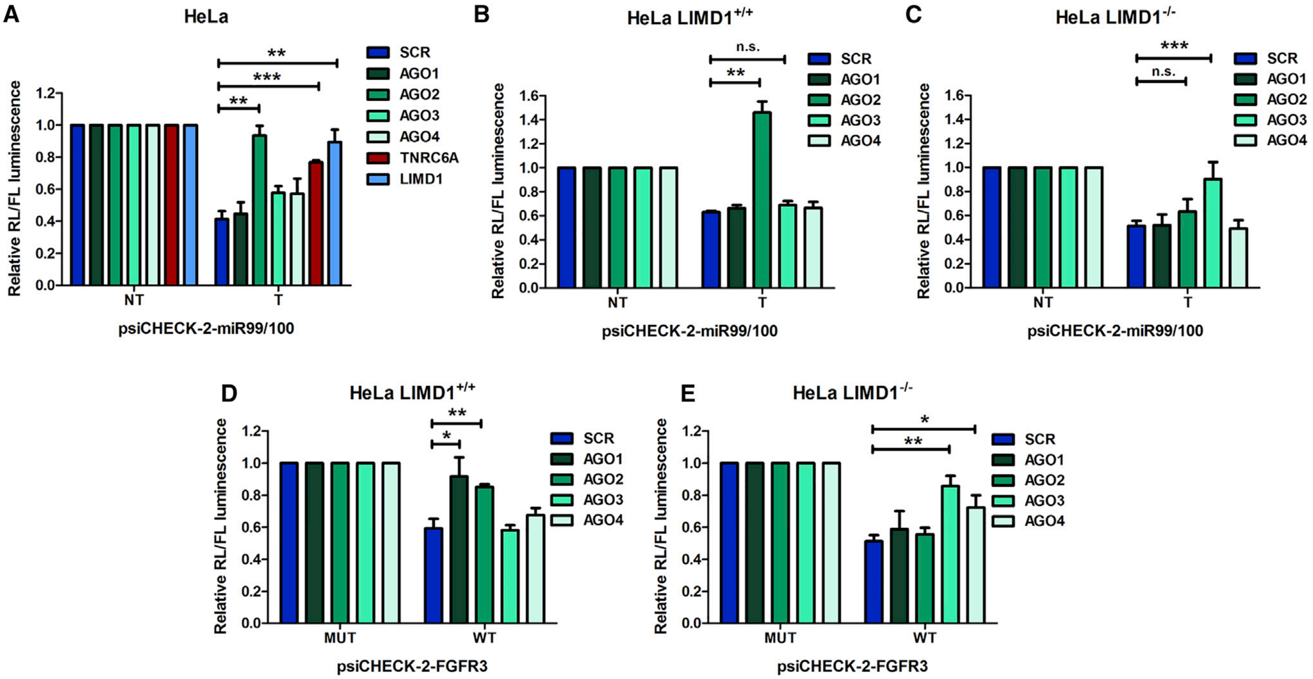

Figure 1. Genetic Ablation of LIMD1 Switches Dependency upon Argonaute Species for miRNA Silencing

(A) psiCHECK-2-miR-99/100 luciferase reporter (NT, non-targeting reporter; T, targeting reporter; mean ± SEM; n = 4 treated with indicated siRNAs (SCR, non-targeting control).

(B and C) (B) psiCHECK-2-miR99/100 reporter in CRISPR-Cas9 gene-edited HeLa cells with Cas9 alone control ($LIMD1^{+/+}$) or (C) LIMD1 knockout ($LIMD1^{-/-}$) cells, treated with the indicated siRNAs.

(D and E) (D) psiCHECK-2-FGFR3 (endogenous mutant [MUT] or wild-type [WT] 3′ UTR) reporter in CRISPR-Cas9 gene-edited HeLa cells with Cas9 alone control ($LIMD1^{+/+}$) or (E) LIMD1 knockout ($LIMD1^{-/-}$) cells, treated with the indicated siRNAs.

appeared to render AGO2 unable to contribute to silencing of both synthetic and natural 3′ UTRs and how the switch in AGO utilization was facilitated. Immunofluorescence (IF) staining of endogenous LIMD1, AGO2, and TNRC6A showed triple colocalization to processing bodies (P-bodies) (Figure 2A). To investigate direct endogenous association of AGO2 and TNRC6A with LIMD1 in situ, we performed proximity ligation assays (PLA) (Söderberg et al., 2006) for AGO2:LIMD1 and TNRC6A:LIMD1 (Figures 2B and S2A). We observed PLA signal for LIMD1 with both AGO2 and TNRC6A. In agreement with recent reports (Chen et al., 2014; Mathys et al., 2014), our PLA analysis demonstrated TNRC6A directly associated with CNOT9 (CNOT9 contains W-binding pockets to accommodate tryptophan residues from TNRC6A). By contrast CNOT1 did not exhibit PLA signal with TNRC6A; these data may be reflective of the lack of direct binding evidence between these proteins and the absence of a structurally resolved binding interface (Chen et al., 2014; Mathys et al., 2014). This supports the validity and specificity of the endogenous LIMD1 direct interactions with AGO2/TNRC6A.

LIM-domain-containing proteins are characterized by their ability to act as scaffolds in multi-protein complexes (Koch et al., 2012). We therefore reasoned that the close proximity and association observed between LIMD1, AGO2, and TNRC6A could indicate a scaffolding role for LIMD1 within the miRISC. We examined the endogenous colocalization of AGO2 with TNRC6A and miRISC component DDX6 by IF in HeLa cells upon treatment with siRNA targeting *LIMD1* (Figures S2B–S2D). LSM1 is required for the assembly of P-bodies but does not affect miRNA-silencing function; we therefore included siRNA targeting LSM1 as a control for P-body disruption. LIMD1 depletion did not disrupt the colocalization of AGO2 with TNRC6A or DDX6. By contrast, and as described in the literature, LSM1 knockdown caused a decrease in visible AGO2/TNRC6A/DDX6 P-bodies (Chu and Rana, 2006).

To rule out a possible false-negative effect due to the limited resolution of confocal microscopy (240 nm) (Nature Photon, 2009), we repeated our analysis using PLA, which detects proteins within 40 nm of each other (Figures 2C–2F). PLA analysis demonstrated that interaction of AGO2 with TNRC6A and DDX6 was significantly reduced upon LIMD1 knockdown, whereas LSM1 knockdown did not reduce this interaction, and in fact increased interaction of AGO2 with TNRC6A. In agreement with these data, reporter assays performed in cells treated with the same siRNAs demonstrated significant de-repression of a miR-99/100 reporter upon loss of LIMD1 but not LSM1 (Figure S2E). These findings confirm that assembly of P-bodies is a consequence rather than a cause of miRNA silencing (Eulalio et al., 2007) and demonstrate that the de-repressive effect of LIMD1 depletion on miRNA silencing is not dependent on the disassembly of P-bodies. Together, our data show that LIMD1 contributes to maintaining the interactions of AGO2 with TNRC6A and DDX6.

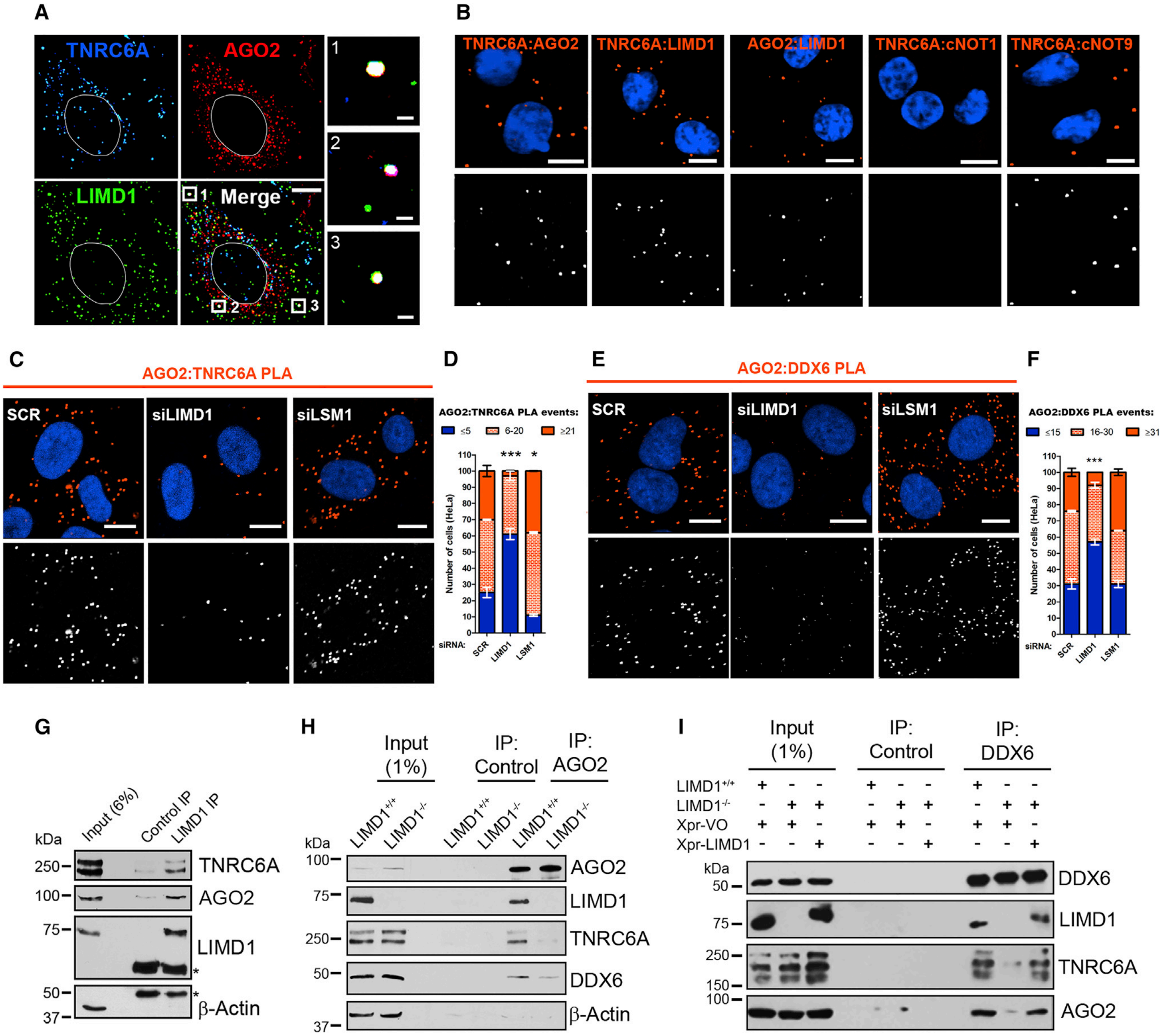

Figure 2. LIMD1 Interacts with miRISC Components AGO2 and TNRC6A and Promotes Their Association

(A) Endogenous IF of the indicated proteins in HeLa cells.

(B) Endogenous in situ interaction determined by proximity ligation assay (PLA) of the indicated proteins. PLA signal orange, cells stained with DAPI (top); PLA signal white for visual clarity (bottom). Scale bars, 10 μm.

(C) PLA analysis of endogenous AGO2 and TNRC6A interaction in HeLa cells treated with SCR (non-targeting), LIMD1, or LSM1 siRNA. PLA signal orange, cells stained with DAPI (top); PLA signal white for visual clarity (bottom).

(D) Quantification of PLA interaction events in (C), displayed as a stacked histogram. Data are mean ± SEM, n = 3, **$p < 0.001$, ***$p < 0.0001$, determined using the chi-square test.

(E) PLA analysis of endogenous AGO2 and DDX6 interaction, as in (C).

(F) Quantification of (E) as in (D).

(G) Endogenous co-immunoprecipitation of LIMD1 with the indicated proteins from HeLa cells (*IgG heavy chain).

(H) Endogenous coIP of indicated miRISC components with AGO2 from CRISPR-Cas9 gene-edited HeLa LIMD1$^{+/+}$ or LIMD1$^{-/-}$ cells.

(I) Endogenous coIP of indicated miRISC components with DDX6 from CRISPR-Cas9 gene-edited HeLa LIMD1$^{+/+}$ or LIMD1$^{-/-}$ cells expressing Xpress (Xpr) vector only (VO) or LIMD1.

In order to further pursue the discrepancy between protein localization to P-bodies and the interaction between miRISC proteins, we performed endogenous IF for AGO1, 2, 3, and 4 with both TNRC6A and DDX6 in the isogenic *LIMD1*$^{-/-}$ HeLa CRISPR pair (Figures S3A and S3B). Loss of LIMD1 had no visible effect on AGO1, 2, or 3 colocalization with TNRC6A or

DDX6 in P-bodies (AGO4 did not colocalize with TNRC6A/DDX6 in either cell line). Furthermore, we did not detect any change in colocalization of EYFP-tagged AGO2 with endogenous TNRC6A upon inhibition of Akt3 by siRNA, treatment with Akt inhibitor MK-2206, or overexpression of AGO2 S387A point mutant (Figure S4A). We therefore performed complementary PLA and endogenous immunoprecipitation experiments and observed strikingly that *LIMD1* knockout caused significant impairment of endogenous AGO2 interaction with TNRC6A and DDX6 (Figures S4B–S4G), in agreement with functional reporter assays performed in these lines (Figure 1). Furthermore, re-expression of Xpress (Xpr)-tagged LIMD1 in the *LIMD1*$^{-/-}$ line restored the interaction of DDX6 with AGO2 and TNRC6A (Figure 2I), potentially explaining the enhancement of silencing observed when LIMD1 is overexpressed in a *LIMD1*-null background (Figure S1D). These data therefore demonstrate that endogenous interaction assays, including PLA and IP, are more accurate methods of determining endogenous miRISC protein-protein interaction, as opposed to visible colocalization within P-bodies. In summary, loss of LIMD1 profoundly impaired the ability of AGO2 to interact with TNRC6A and downstream effectors within miRISC required for miRNA-mediated silencing.

LIMD1 Interacts with AGO2 and TNRC6A via Specific Domains

Having demonstrated that LIMD1 is required to scaffold the interaction of AGO2 and TNRC6A, we next sought to map the interaction interfaces on all three proteins. LIMD1 contains three tandemly arrayed LIM domains (protein-protein interacting zinc fingers) in its C-terminal region and a preceding proline/serine-rich unstructured domain referred to as the pre-LIM (residues 1–471). Our previous work demonstrated that AGO2 bound to the pre-LIM portion of LIMD1 (James et al., 2010). To identify the precise AGO Binding (AB) motif in the pre-LIM, we co-immunoprecipitated Xpr-tagged full-length LIMD1 or a series of LIMD1 internal deletion mutants with EYFP-tagged AGO2. We found that deletion of amino acids 140–166 within LIMD1 abrogated the interaction with AGO2 (Figure 3A). The identification of amino acids 140–166 as the AB motif in LIMD1 was confirmed by direct binding assays using purified un-tagged AGO2 (hAGO2) (De and Macrae, 2011) (Figure 3B) and maltose binding protein (MBP)-tagged full-length LIMD1 or AB motif (Figure 3C). Generation of a set of HeLa lines lentivirally transduced to express sh-SCR (non-targeting) or sh-LIMD1 RNA and RNAi-resistant wild-type (WT) LIMD1 (rr-LIMD1) or LIMD1 deletion mutants (Figure S5A) demonstrated loss of silencing of the miR-99/100 reporter in sh-LIMD1 and rr-Δ140–166 lines (Figure 3D). In contrast, we observed that cells expressing the larger adjacent rr-Δ186–260 LIMD1 deletion mutant (which retains AGO2 binding, Figure 3A) displayed equivalent levels of silencing to that seen in rr-LIMD1 cells, thus providing an additional control for the impact of internal deletions on the secondary structure of the pre-LIM domain. These data indicate that, as predicted, the ability of LIMD1 to bind AGO2 via its AB motif is critical for its miRNA-silencing function.

We next performed complementary direct binding assays to identify the corresponding LIMD1-binding domain within AGO2, using a series of GST-AGO2 N- and C-terminal truncation mutants (Figures S5B and S5C). We determined that the Linker 2 (L2) domain of AGO2, which lies between the PAZ and MID domains, was necessary for direct interaction with the AB motif of LIMD1 (MBP-LIMD1 140–166) and full-length LIMD1 (Figure 3E). We note that the additional bands present in these purified AGO2 mutants are either degradation products (as determined by western blot and mass spectrometry (Figures S5D–S5G) or bacterial Hsp70 (DnaK), which did not affect binding to LIMD1 (Figure S5H).

As our PLA experiments also indicated a close association of LIMD1 with TNRC6A, we sought to confirm whether this occurred independently of AGO2. We found that deletion of the LIM domains (C-terminal) of LIMD1 resulted in loss of interaction with EGFP-tagged TNRC6A in co-immunoprecipitation experiments (Figure 3F). Deletion of the LIMD1 AB motif did not affect interaction with TNRC6A, demonstrating that the interaction of LIMD1 with TNRC6A is not dependent upon LIMD1 interaction with AGO2. Next, we determined that the N-terminal AGO-binding domain (ABD) of TNRC6A (Eulalio et al., 2009b) was responsible for binding to LIMD1; coIP experiments in HEK293T cells using EGFP-tagged TNRC6A truncations and Xpr-LIMD1 revealed that deletion of TNRC6A amino acids 1–883 resulted in complete loss of binding to LIMD1 (Figure 3G).

In summary, the pre-LIM region of LIMD1 is responsible for interaction with AGO L2 (via the AB, aa 140–166), and the C-terminal LIM domains interact with the N-terminal portion of TNRC6A. Therefore, we provide evidence that LIMD1 functions as a central scaffold protein and a core component of miRISC, forming an integral link between AGO2 and TNRC6A through simultaneous direct contacts with each (Figure 3H).

Akt3-Mediated Phosphorylation of S387 in the AGO2 L2 Domain Is Necessary for Interaction with LIMD1

It has been proposed that Akt3-dependent phosphorylation of AGO2 S387 promotes its miRNA function and opposes its target cleavage activity (Horman et al., 2013). AGO2 S387 phosphorylation was suggested to increase the association of AGO2 with TNRC6A via an unknown mechanism. As S387 is located within the L2 region of AGO2, we tested whether phosphorylation of this residue could trigger the association between AGO2 and LIMD1 and subsequent recruitment of TNRC6A to AGO2. We generated constructs expressing phospho mutant (AGO2 S387A) or phospho-mimic (AGO2 S387E) versions of EYFP-AGO2 and compared their ability to interact with LIMD1 in immunoprecipitation assays. AGO2 S387A had reduced binding to LIMD1, whereas the phospho-mimic version of AGO2 (AGO2 S387E) rescued interaction with LIMD1 (Figures 4A and S6B). The interaction profile of LIMD1 with these AGO2 constructs was mirrored by endogenous TNRC6A and DDX6, which displayed significantly reduced binding to AGO2 S387A, and rescue of binding with AGO2 S387E (Figures S6A and S6B). The importance of S387 phosphorylation for LIMD1 binding was further confirmed by direct binding assays between the AB motif of LIMD1 and GST-AGO2 WT or GST-AGO2 S387E, which revealed enhanced binding of the AGO2 phospho mimic to LIMD1 AB motif (Figure 4B). The dependency on S387 for LIMD1 association was also evident in the degree of colocalization of AGO2 and LIMD1 in U2OS cells transfected with

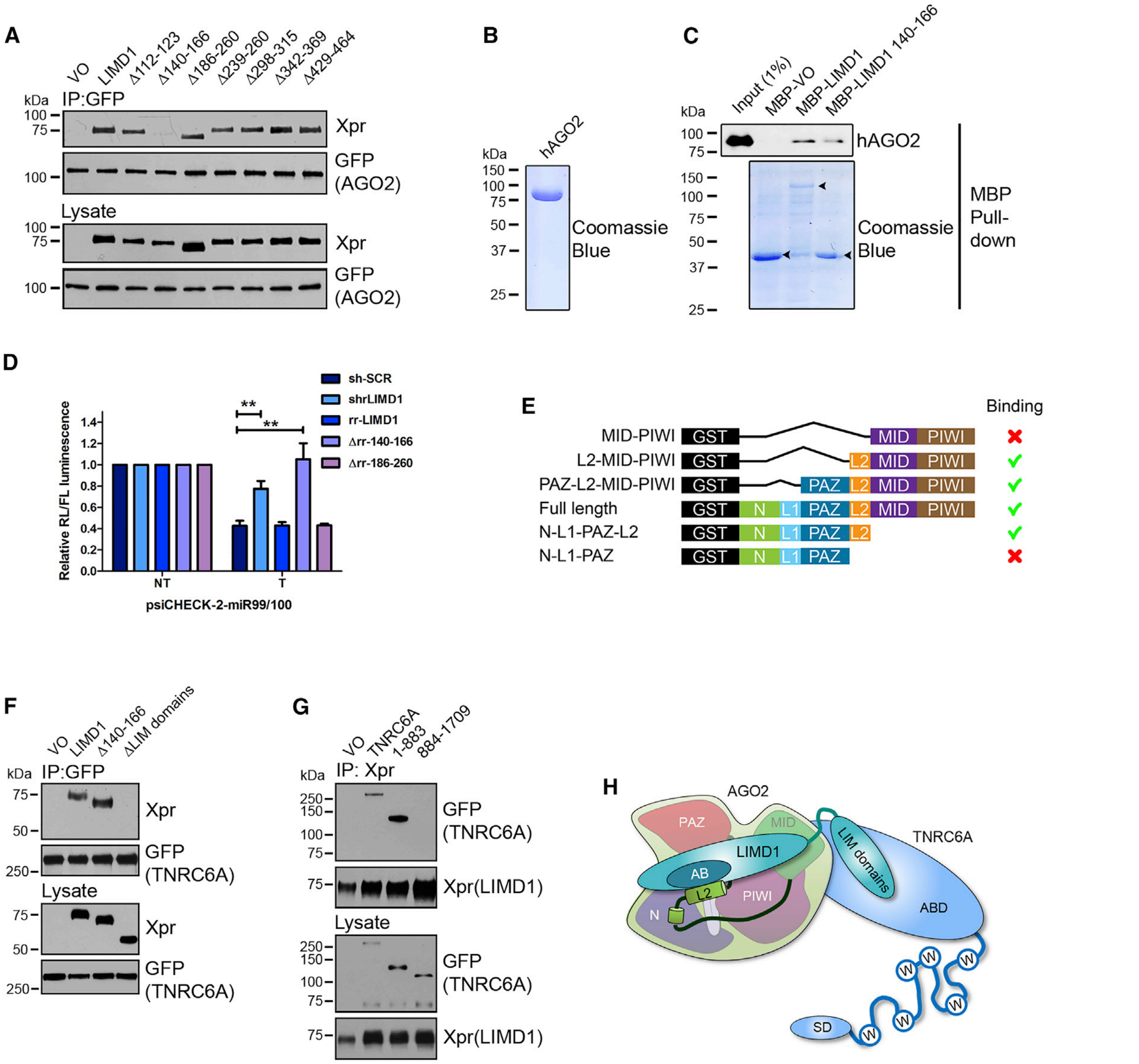

Figure 3. LIMD1 Bridges AGO2 and TNRC6A via Distinct Interaction Sites

(A–C) (A) Co-immunoprecipitation (coIP) of Xpress (Xpr)-LIMD1 and internal deletion mutants with GFP-AGO2 from HEK293T lysate. (B) Purified un-tagged crystallography grade hAGO2 used in (C) direct binding assay with MBP-VO, MBP-LIMD1, or MBP-LIMD1 140 166 (AB motif) (highlighted by arrows).

(D) psiCHECK-2-miR99/100 luciferase reporter (NT [non-targeting], T [targeting]) assay performed in shRNA knockdown and rescue (RNAi resistant [rr]) LIMD1 HeLa cells (mean ± SEM; n = 3).

(E) Schematic of GST-AGO2 truncation mutants and summary of binding to LIMD1 AB motif.

(F) CoIP of Xpr-LIMD1 and deletion mutants with GFP-TNRC6A from HEK293T lysate.

(G) CoIP of TNRC6A and truncation mutants with Xpr-LIMD1 from HEK293T lysate.

(H) Graphical representation of domain interaction of AGO2, LIMD1, and TNRC6A. The AB motif of LIMD1 (aa 140–166) directly binds linker 2 (L2) of AGO2, and the LIM domains of LIMD1 bind the N-terminal AGO-binding domain (ABD) of TNRC6A (SD, silencing domain).

mTan-LIMD1 and the EYFP-AGO2 constructs (Figures 4C, 4D, and S6C). Compared to WT AGO2, AGO2 S387A and S387E showed a 9.4-fold decrease and 2.1-fold increase in colocalization with LIMD1, respectively. These data indicate that the interaction of AGO2 with LIMD1 is dependent on the phosphorylation status of AGO2 S387.

Next, we tested whether the interaction between LIMD1 and AGO2 could be directly promoted by overexpression of Akt3, thought to be the primary kinase responsible for phosphorylation of AGO2 S387 (Horman et al., 2013). We immunoprecipitated endogenous LIMD1 from serum-starved HeLa cells transfected with an empty vector or a constitutively active myristoylated Akt3 (HA-myr-Akt3) (Song et al., 2008) and found that Akt3 significantly enhanced both the levels of phospho-S387 AGO2 (using a phospho-S387-specific antibody (Zeng et al., 2008; Figure S6D) and the interaction of AGO2 with LIMD1 (Figure 4E).

In a complementary assay, treatment of the HeLa CRISPR-Cas9 isogenic pair with the Akt inhibitor MK-2206 demonstrated that interaction of AGO2, LIMD1, and TNRC6A with DDX6 was significantly impaired in *LIMD1*$^{+/+}$ cells upon Akt inhibition (Figure 4F); under these conditions, the interaction of the complex was equivalent to that of the *LIMD1*$^{-/-}$ line (Figure S6E). Treatment of the *LIMD1*$^{-/-}$ cells with MK-2206 entirely abolished the complex interactions, indicating that there is some residual interaction of AGO2 with miRISC in the absence of LIMD1, which is also Akt dependent. In agreement with these assays, siRNA-mediated knockdown of Akt3 in U2OS cells resulted in a 9-fold reduction in colocalization between EYFP-AGO2 and mTan-LIMD1 (Figures 4G, 4H, and S6F). In summary, these data confirm that Akt3-mediated phosphorylation of AGO2 S387 is required for AGO2-LIMD1 interaction.

Activated Phospho-S387-AGO2 Requires LIMD1 for Formation of a Functional AGO2-miRISC

To determine whether phosphorylated AGO2 (S387) is dependent on LIMD1 for miRISC formation and function, we examined the activity of the miR-99/100 reporter in HeLa CRISPR lines treated with siRNA against AGO2, where WT AGO2 or S387 phospho-site mutants were re-expressed (Figures 5A, 5B, and S6G). In *LIMD1*$^{+/+}$ cells, AGO2 knockdown caused the expected de-repression. Re-expression of AGO2 WT and S387E rescued silencing, whereas AGO2 S387A was unable to rescue silencing, in agreement with published data (Horman et al., 2013). In contrast, in the *LIMD1*$^{-/-}$ line, AGO2 knockdown did not cause any de-repression, and re-expression of any AGO2 construct (WT or S387 mutant) similarly had no effect.

We next determined whether the previously established regulation of AGO2 miRNA silencing by Akt3 was in fact mediated by LIMD1 (Horman et al., 2013). Upon siRNA-mediated depletion of Akt3, we observed de-repression of the miR-99/100 reporter in *LIMD1*$^{+/+}$ cells, but this had no effect in the *LIMD1*$^{-/-}$ line, where silencing was maintained (Figures 5C and S6H). Taken together, these data demonstrate that the activating effect of Akt3-mediated phosphorylation on AGO2 is dependent on subsequent interaction with LIMD1. In the absence of LIMD1, miRNA silencing still occurs independently of Akt3. To investigate how LIMD1 loss caused this striking inhibition of phospho-AGO2 activity, we examined the interaction of phospho-AGO2 with TNRC6A and DDX6 upon loss of LIMD1. PLA analysis with the phospho-AGO2 (S387)-specific antibody demonstrated that loss of LIMD1 significantly disrupts the ability of phospho-AGO2 to bind both TNRC6A and DDX6 endogenously (Figures 5D–5G, S6I, and S6J). These data therefore demonstrate that AGO2 phosphorylation by Akt3 has no activating effect on miRNA silencing in the absence of LIMD1 due to the fact that the loss of this scaffold protein dissociates phospho-AGO2 from TNRC6A/miRISC and precludes AGO2-dependent miRNA silencing. These data are in agreement with the functional observations made upon LIMD1 loss (Figure 1), where we observed functional replacement by AGO3. We therefore went on to examine whether this mechanism of phospho-dependent recruitment of LIMD1 family proteins was conserved across all human AGO proteins.

AGO1 and AGO4 Exhibit Phosphorylation-Dependent Interaction with LIMD1 Family Proteins

A significant structural homology exists between the four human AGO proteins; our alignment analysis revealed that AGO2 S387 is conserved in the L2 domains of AGO1 and AGO4 (Figure 6A). We therefore investigated whether there was a conserved phospho-dependent mechanism of AGO1, 2, and 4 interaction with LIMD1 family members. First, we found that LIMD1, Ajuba, and WTIP interacted with all four AGOs (Figures S7A, S7B, and data not shown). LIMD1 also directly bound to the L2 domain of AGO1 (Figure S7C), and, in a similar manner to AGO2, mutation of the conserved serine in AGO1 (S385) to alanine inhibited colocalization with LIMD1, whereas mutation to glutamic acid rescued this colocalization (Figures S7D and S7F). Furthermore, knockdown of Akt3 by siRNA significantly reduced AGO1:LIMD1 colocalization, suggesting that Akt3 may also phosphorylate AGO1 S385 (Figures S7E and S7G). Point mutation of the conserved serine (S377) in AGO4 produced the same disruption and rescue of colocalization with LIMD1 as observed with AGO1 and 2 (Figures S7H and S7J). Taken together these data suggest a common mechanism may exist whereby human AGO 1, 2 and 4 are activated for miRNA-silencing function by phosphorylation of a conserved serine residue, which directs their interaction with LIMD1 and family members and subsequent recruitment to TNRC6A/miRISC. Given the ability of LIMD1 to colocalize with three AGO proteins in a phospho-dependent manner, we also demonstrated that the LIMD1 family member Ajuba, also colocalized with AGO1 and that this was dependent on the phosphorylation status of S385 (Figure S7I). These data support a fundamentally conserved mechanism of phosphorylation-dependent activation of AGO proteins by recruitment and regulation of all three members of this LIM-domain-containing family, LIMD1, Ajuba, and WTIP.

AGO3 Interacts with LIMD1 Family Proteins Independently of Akt3 Phosphorylation

In contrast to AGO1 and AGO4, AGO3 possesses a phosphorylation-mimicking glutamic acid residue at amino acid position 390 (Figure 6A). Given that we found AGO3 can rescue loss of AGO2 miRNA function in the absence of LIMD1, we postulated that this phospho-mimic residue may facilitate interaction with LIMD1 and/or its closely related family members Ajuba/WTIP independently of Akt3 signaling. This could be a mechanism explaining the switch to AGO3 utilization upon deletion of LIMD1 (Figure 1).

To interrogate this hypothesis, we first determined that AGO3 colocalizes with LIMD1, consistent with IP data (Figure S7A), and that this is significantly reduced upon mutation of the phosphomimic residue to alanine (E390A) (Figures 6B and 6C). Similarly, colocalization of AGO3 with Ajuba was reduced by mutation of E390 to alanine (Figures S7K and S7L). In striking contrast to AGO2, siRNA-mediated knockdown of Akt3 did not affect AGO3/LIMD1 colocalization (Figures 6D and 6E). WTIP interaction with AGO3 was also unaffected by Akt inhibition (MK-2206), as opposed to AGO2, which demonstrated loss of interaction (Figures 6F and 6G), and siRNA-mediated knockdown of Akt3 similar had no effect on AGO3 colocalization with WTIP

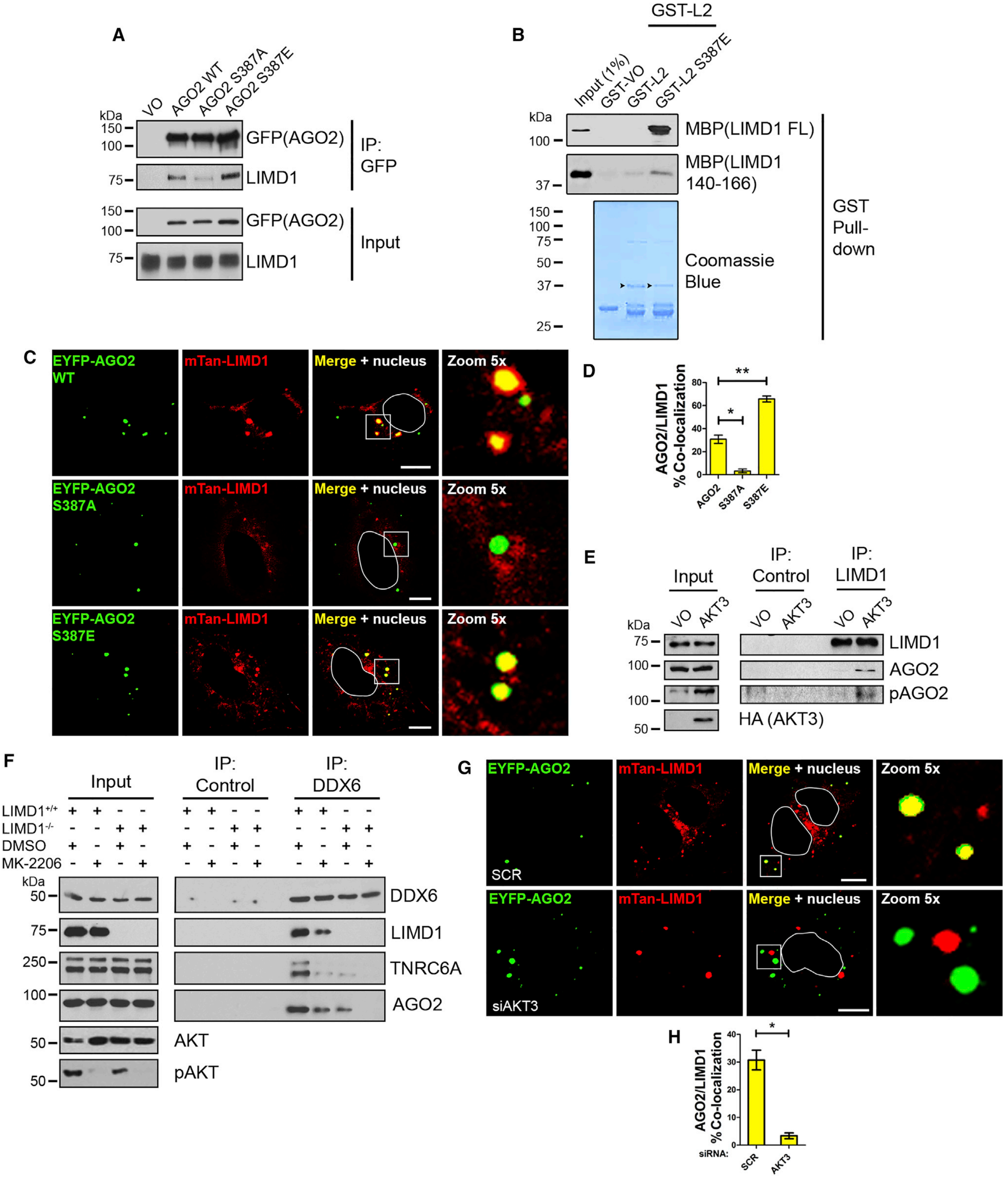

Figure 4. Binding of LIMD1 to AGO2 Is Dependent on Akt3-Mediated S387 Phosphorylation

(A) Co-immunoprecipitation (IP) of Xpr-LIMD1 with EYFP-AGO2 WT, AGO2S387A (phospho-deficient), and AGO2S387E (phospho-mimic) point mutants from HEK293T lysate.

(B) Purified recombinant GST-AGO2 L2 or L2 S387E were used in direct binding assay with MBP-LIMD1 full-length or AB motif (140–166).

(legend continued on next page)

(Figures 6H and 6I). These data therefore demonstrate that AGO3 interaction with LIMD1 family proteins occurs independently of Akt signaling as a result of the naturally occurring phospho-mimic residue E390.

We observed activation of AGO3 miRNA function when LIMD1 protein expression was entirely ablated (Figure 1), and phosphorylated AGO2 was unable to engage with TNRC6A and silencing effectors (Figure 5). In the *LIMD1*$^{-/-}$ HeLa cell line, siRNA depletion of WTIP yielded a significant de-repression of the miR-99/100 reporter, whereas Ajuba knockdown did not (data not shown). Therefore, to determine whether WTIP was responsible for engaging AGO3 in the absence of LIMD1, we performed siRNA knockdown of AGO3 or WTIP, singularly or in combination, in the *LIMD1*$^{-/-}$ HeLa cell line. We discovered that WTIP is required for silencing of a miR-99/100 reporter in these cells (Figure 7A). Furthermore, depletion of WTIP and AGO3 in combination led to an almost complete de-repression of silencing (Figure 7A). Concurrent with this, we discovered an increase in the number of AGO3 P-bodies in the *LIMD1*$^{-/-}$ line compared to *LIMD1*$^{+/+}$ (Figures S7M and S7N), indicating that while a reduction in an AGO miRNA-silencing function is not necessarily associated with dissociation from P-bodies (as is the case with AGO2), an increase in AGO3 silencing function may be associated with an increase in its recruitment to P-bodies. Furthermore, we observed an increase in AGO3:WTIP interaction in the *LIMD1*$^{-/-}$ line compared to *LIMD1*$^{+/+}$ (Figures 7B and 7C).

AGO function in miRNA silencing is critically dependent on association with TNRC6 proteins, which enable recruitment of several effector complexes that mediate target mRNA decapping and deadenylation. We therefore examined whether TNRC6A recruitment to AGO2 and AGO3 was altered in the presence and absence of LIMD1, which would explain the apparent switch in AGO function. Endogenous immunoprecipitation of AGO2 or AGO3 from the HeLa *LIMD1*$^{-/-}$ CRISPR cell line pair demonstrated that, in contrast to the loss of TNRC6A interaction with AGO2, AGO3 interaction with TNRC6A was significantly enhanced upon loss of LIMD1 (Figures 7D and 7E).

These interaction data elucidate the mechanism by which loss of LIMD1 causes a switch in cellular AGO dependency (Figure 1). We have discovered that, in HeLa cells, loss of LIMD1 causes dependency upon AGO2 to switch to AGO3, and this is associated with enhanced (phosphorylation-independent) interaction of AGO3 with LIMD1 family member WTIP, and with TNRC6A as the effector of silencing. These data therefore define the mechanism by which AGO2-S387 phosphorylation is an activating event for silencing. Additionally, as all LIMD1 family proteins interact with AGO1–4 in a phospho-dependent manner, these data suggest a general mechanism whereby specific AGO proteins are activated for miRNA function by their phosphorylation and subsequent recruitment of LIMD1 family proteins, thus enabling miRISC formation.

DISCUSSION

LIMD1 Acts as a Molecular Clamp to Secure Phospho-AGO2 and TNRC6A Together within miRISC

We determined that LIMD1 binds directly to AGO2 and TNRC6A at distinct interfaces: the AGO-binding motif (AB motif) of LIMD1 binds the L2 domain of AGO2, and the C-terminal LIM domains of LIMD1 bind the amino-terminal ABD of TNRC6A, independently of AGO binding (Figure 3) (Eulalio et al., 2009a; Pfaff et al., 2013; Takimoto et al., 2009). It is important to note that our data do not suggest the AGO-TNRC6A association can only occur via LIMD1; the MID/PIWI (P-element induced wimpy testis) domains of AGO2 that directly bind TNRC6A tryptophan residues do not bind to LIMD1 (Figure 3E) (Schirle and MacRae, 2012). Additionally, the association of the C-terminal silencing domain of TNRC6A with PABP/CNOT9/CNOT1/DDX6 does not preclude the binding of LIMD1, which interacts with the N-terminal half of TNRC6A (Figure 3D) (Eulalio et al., 2009a). Therefore, our data indicate that LIMD1 acts as a molecular "clamp" by binding to both AGO and TNRC6A proteins simultaneously to secure their association and commit both proteins and associated effectors to the miRNA-silencing pathway: AGO2 association with TNRC6A/miRISC and its miRNA-silencing activity is significantly impaired upon LIMD1 loss (Figures 1A–1C and 2).

It has been demonstrated that activation of miRNA silencing is dependent on Akt3-mediated phosphorylation of AGO2 at S387 (Horman et al., 2013). We have determined that this phosphorylation promotes association of AGO2 with LIMD1 and subsequent recruitment of TNRC6A and downstream silencing effector complexes (Figures 4 and 5). Our findings therefore reveal the functional consequence of S387 phosphorylation, which underpins an apparent preference of AGO utilization directed by LIMD1 family member proteins. In agreement with our findings, La Rocca et al. recently identified phosphorylation-dependent activation of miRNA silencing by PI3K, a kinase that lies upstream of Akt3 (La Rocca et al., 2015). Although this study did not examine the precise changes in AGO2 phosphorylation, it discovered an increase in the molecular mass of AGO2 upon pathway activation, most likely via the recruitment of other effector proteins, which would include TNRC6A, DDX6, and, from our data, LIMD1.

(C) Co-localization of EYFP-AGO2/S387 point mutants with mTan-LIMD1 in U2OS cells. Nucleus outline in white.
(D) Quantification of percentage of AGO2/S387 P-bodies colocalized with LIMD1 in (C).
(E) Immunoprecipitation of endogenous LIMD1 from HeLa cells transfected with HA-VO or HA-myristolated Akt3, and western blot analysis of the indicated proteins; pAGO2 is S387.
(F) Immunoprecipitation of DDX6 from HeLa LIMD1$^{+/+}$ or LIMD1$^{-/-}$ treated with DMSO or Akt inhibitor MK-2206 and analyzed for the indicated proteins by western blot; pAKT is S473.
(G) Co-expression of EYFP-AGO2 (WT) with mTan-LIMD1 in U2OS cells treated with non-targeting (SCR) or AKT3 siRNA. Nuclear outlines in white.
(H) Quantification of number of AGO2 P-bodies colocalized with LIMD1 in (G).
Data shown are mean ± SEM, n = 3, *p < 0.05, **p < 0.001. Scale bars, 10 mm.

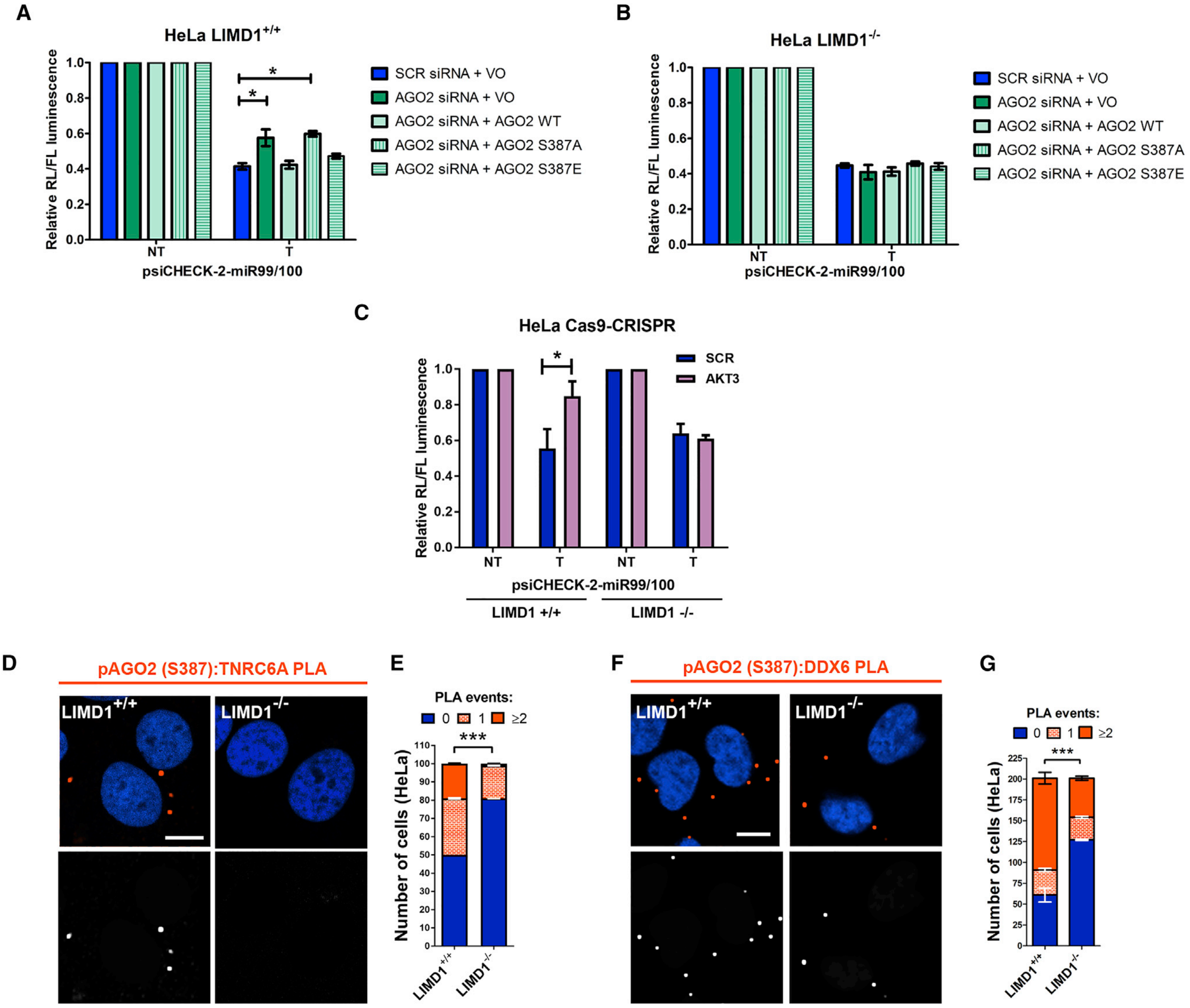

Figure 5. LIMD1 Is Required for Phospho-S387-AGO2 to Engage TNRC6A/DDX6 for miRNA-Mediated Silencing

(A and B) (A) psiCHECK-2-miR99/100 reporter assay in HeLa CRISPR-Cas9 LIMD1$^{+/+}$ or (B) LIMD1$^{-/-}$ cell lines transfected with non-targeting (SCR) or AGO2 siRNA and EYFP-AGO2, EYFP-AGO2S387A, or EYFP-AGO2S387E.

(C) miR99/100 reporter assay in in above HeLa CRISPR-Cas9 cell lines transfected with non-targeting (SCR) or AKT3 siRNA.

(D) PLA analysis of endogenous phospho-AGO2 (S387):TNRC6A interaction in situ in HeLa CRISPR-Cas9 LIMD1$^{+/+}$ or LIMD1$^{-/-}$ lines. PLA signal orange, cells stained with DAPI (top); PLA signal white for visual clarity (bottom). Scale bars, 10 μm.

(E) Quantification of PLA interaction events in (D), displayed as stacked histograms. Data are mean ± SEM, n = 3, total of 200 cells determined using the chi-square test.

(F) PLA analysis of phospho-AGO2 (S387):DDX6 interaction in above HeLa CRISPR-Cas9 cell lines.

(G) Quantification of (F) as in (E).

Unless otherwise stated, data are mean ± SEM, n = 3, *p < 0.05, **p < 0.001, ***p < 0.0001 according to the Student's t test.

AGO Interaction with LIMD1 Family Proteins Occurs via a Common Phosphorylation-Dependent Mechanism

The AGO L2 domain is a 98-residue, extended L-shaped coil that connects the PAZ and MID domains of AGO proteins (Elkayam et al., 2012; Schirle and MacRae, 2012). The AGO3 S387 residue phosphorylated by Akt3 is conserved in human AGO1 and 4 (Figure 6A). Our data demonstrate that LIMD1 can interact with all four AGO proteins (Figure S8A) and that association with AGO1 and 4 is also dependent on the ability of this conserved serine residue to be phosphorylated (Figures S8D–S8I). Taken together with previous studies demonstrating that AGO1 is phosphorylated (Rüdel et al., 2011), these data support a hypothesis of a common AGO-activating mechanism by Akt3 and/or other unidentified kinases. AGO3 does not contain this

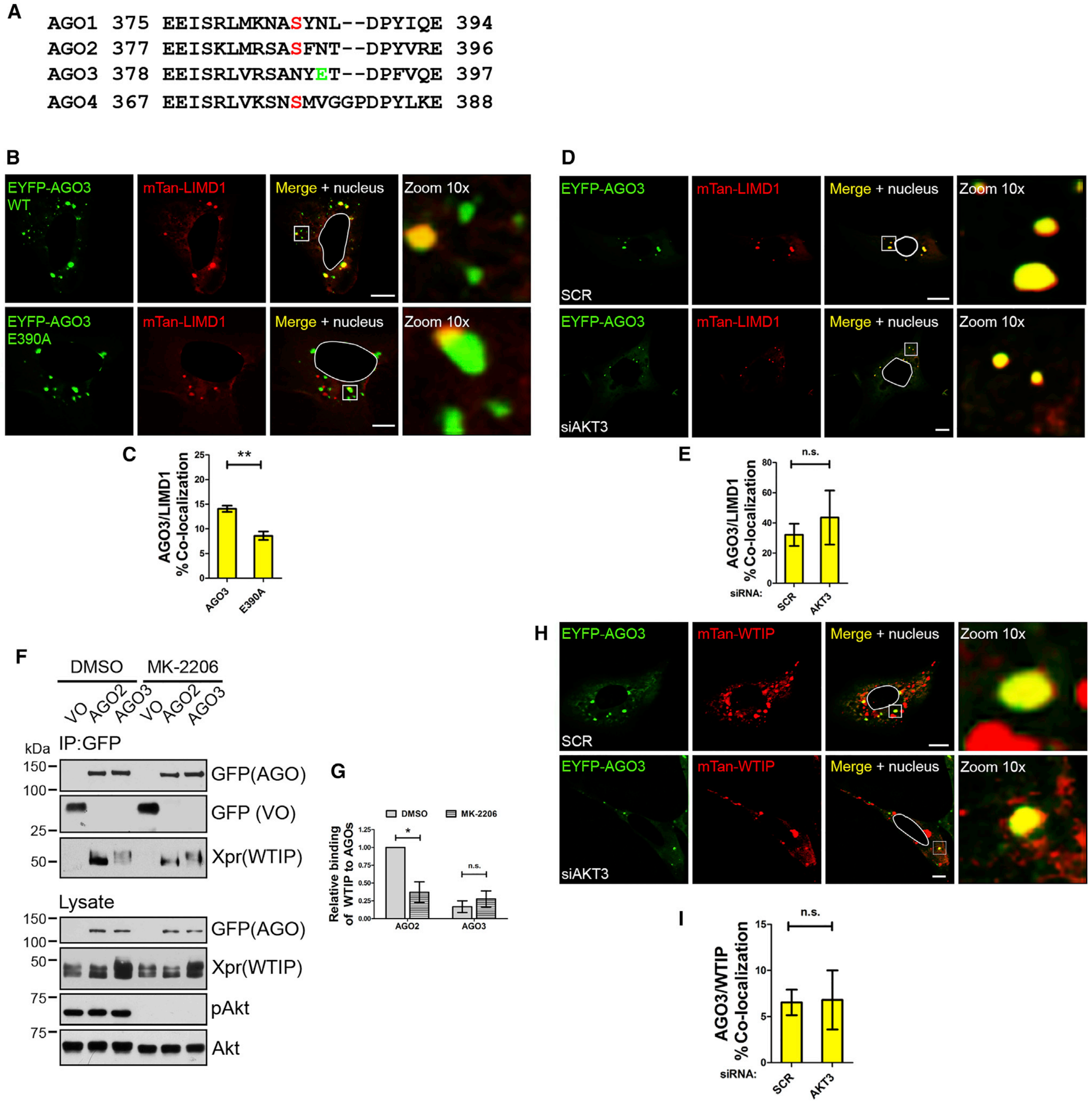

Figure 6. AGO3 E390 Acts as a Phospho-mimic, Facilitating Interaction with LAW Independently of Akt Signaling

(A) AGO2 S387 is highly conserved within AGO1 (S385) and AGO4 (S377). AGO3 does not contain an equivalent serine residue but instead an adjacent phospho-mimic glutamic acid residue (E390).

(B) Colocalization of EYFP-AGO3/E390 point mutants with mTan-LIMD1 in U2OS cells.

(C) Quantification of percentage of AGO3 P-bodies colocalized with LIMD1 in (B).

(D) Colocalization of EYFP-AGO3 with LIMD1 in U2OS cells treated with non-targeting (SCR) or AKT3 siRNA.

(E) Quantification of percentage of AGO3 P-bodies colocalized with LIMD1 in (D), n = 2.

(F) Immunoprecipitation (IP) of EYFP-VO, -AGO2, or -AGO3 from HEK293T lysate co-transfected with Xpress (Xpr)-WTIP and treated with DMSO or Akt inhibitor MK-2206.

(G) Quantification of WTIP interaction with AGO2 and 3, in (F). Data are mean densitometry relative to AGO2 (DMSO), ±SEM, n = 3.

(H) Colocalization of EYFP-AGO3 with WTIP in U2OS cells treated with non-targeting (SCR) or AKT3 siRNA.

(I) Quantification of percentage of AGO3 P-bodies colocalized with WTIP in (H), n = 2.

Unless otherwise stated, quantification data are mean ± SEM, n = 3, *p < 0.05, **p < 0.01, ***p < 0.001, n.s., not significant.

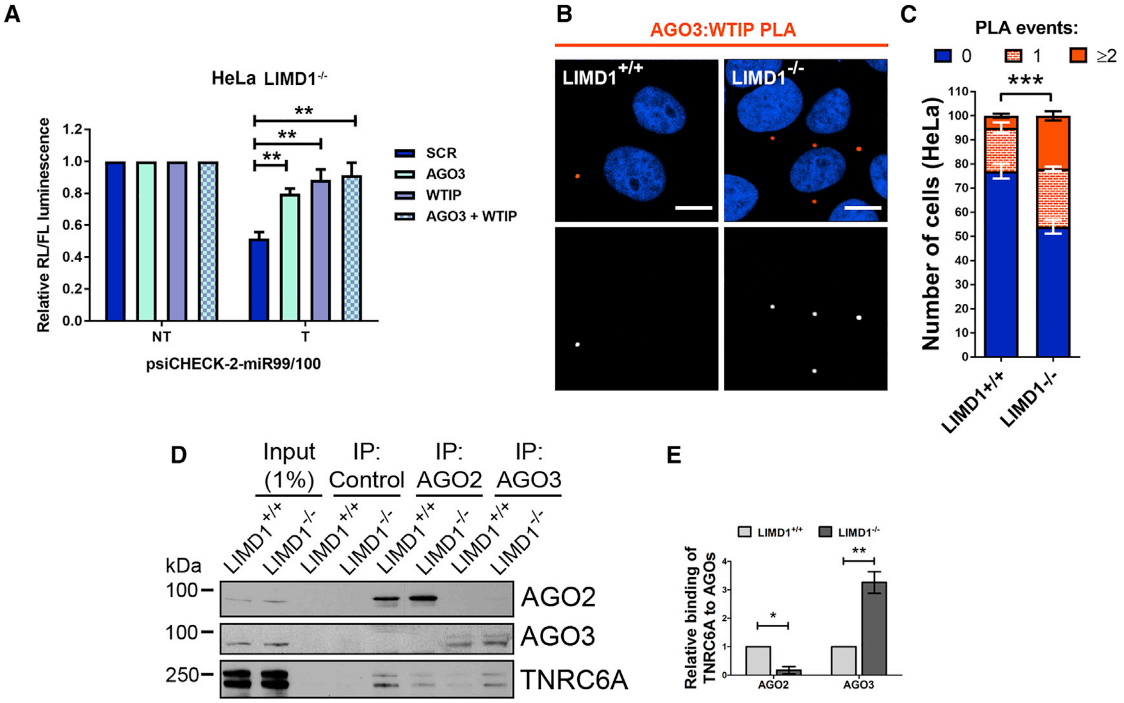

Figure 7. AGO2 miRNA Function Switches to AGO3 with LIM Family Member WTIP in the Absence of LIMD1
(A) LIMD1$^{-/-}$ HeLa cells transfected with non-targeting (SCR) and the indicated siRNAs (**p<0.01 according to student's T-test).
(B) PLA of AGO3 and WTIP in HeLa CRISPR-Cas9 cell lines.
(C) Quantification of PLA interaction events in (B), displayed as a stacked histogram.
(D) IP of endogenous AGO2 or AGO3 from HeLa CRISPR-Cas9 cells, coIP of endogenous TNRC6A.
(E) Quantification of TNRC6A coIP with AGO2 and 3 in HeLa CRISPR-Cas9 cells in (D). Data are mean densitometry relative to LIMD1$^{+/+}$ AGO2/3.
Unless otherwise stated, all data are ±SEM, n = 3, *p < 0.05, **p < 0.001, ***p < 0.0001, n.s., not significant, according to the Student's t test.

conserved serine residue but rather contains a phospho-mimic glutamic acid residue, which directs interaction with LIMD1 family members independently of Akt signaling (Figure 7).

Horman et al. postulated that phosphorylation of S387 represented a molecular switch between the siRNA and miRNA function of AGO2 (Horman et al., 2013). However, our data indicate the Akt3-mediated phosphorylation of AGO1, 2 (and possibly AGO4) could also be considered as a switch from OFF to ON for miRNA-silencing function. Furthermore, if one were to consider the possibility of a cell state with no Akt3 signaling, where silencing may only be possible through AGO3 function, then an activation of Akt3 could activate a switch from the default AGO3 silencing to any or all of the AGO1-, 2-, or 4-dependent mechanisms. Our work also opens up the possibility for functional differences between the four human AGO proteins that have been previously been described to act redundantly in the miRNA pathway (Dueck et al., 2012; Su et al., 2009). Future work will address this possibility.

Although many components and processes of the miRNA pathway are highly conserved between humans and *Drosophila*, an important difference is that, in *Drosophila*, only one of two AGO proteins, AGO1, functions in the miRNA pathway (Förstemann et al., 2007; Tomari et al., 2007). Within the L2 domain of *Drosophila* AGO1, S387 is not conserved. This may suggest that the evolution of S387 in human AGO2 (and equivalent serines in AGO1 and 4) was necessary for coordinating the responses of multiple AGOs to signaling inputs. It remains to be determined whether a protein in *Drosophila* fulfills the role of LIMD1 with regard to promoting interaction between AGO1 and GW182. Recent data from Golden et al. have shown that cyclic phosphorylation at highly conserved serine residues in the PIWI domain of human AGO2 (S824–S834) promotes efficient miRNA silencing (Golden et al., 2017). They noted that these residues are conserved in human AGOs 1–4 and *Drosophila* AGO1 but not in *Drosophila* AGO2, which only functions in the siRNA pathway. Thus, the evolution of several regulatory phospho sites, including S387, may be integral to the expansion of the AGO gene family between fly and human, all of which can function in miRNA-mediated silencing.

LIMD1 Directs AGO Family Member Utilization for miRNA Silencing

While siRNA and shRNA-mediated knockdown of proteins is able to provide functional insights, the complete genetic ablation of a protein can reveal longer-term adaptations in cells and alternative pathways to maintain homeostasis. Through the use of CRISPR-Cas9 technology, we ablated LIMD1 expression and observed a switch in AGO dependency for silencing from AGO2 to AGO3. In the *LIMD1*$^{-/-}$ cell line, we found that phospho-AGO2 S387 and total AGO2 interaction

with TNRC6A and DDX6 was significantly impaired, and AGO2 miRNA function was entirely ablated irrespective of its phosphorylation status (Figure 5). In parallel assays, LSM1, which is required for P-body assembly but not miRNA silencing (Chu and Rana, 2006; Eulalio et al., 2007, 2009a), affected neither AGO2 association with TNRC6A/DDX6 nor AGO2-dependent miRNA silencing (Figures 2C–2F). These data indicate that the effects observed upon LIMD1 loss occur specifically as a result of its scaffold role in the AGO2:TNRC6A association and not as a general effect of loss of P-body structural integrity. Indeed, re-expression of LIMD1 in the *LIMD1*$^{-/-}$ line not only enhanced silencing, but also rescued the interaction of AGO2 with TNRC6A and DDX6 (Figure 2J).

The contributions of each AGO protein to miRNA-mediated silencing has been attributed to their expression levels, with AGO2 being the most abundant and therefore of greatest importance (Wang et al., 2012). However, our data reveal that LIMD1 family proteins are important determinants of AGO utilization and show that AGO levels per se may not necessarily determine functional importance. The typically low AGO3 expression in various tissues and cell types has been ascribed to the high proportion of rarely used codons in its mRNA (Valdmanis et al., 2012). Despite this, we have shown AGO3 does function in miRNA silencing and specifically functions in the absence of the Akt3-AGO2-LIMD1 signaling axis. Determination of AGO utilization and the cooperation of different AGOs may, in addition to specific pairing with LIMD1 family proteins, be context dependent with respect to miRNA site geometry in the 3′ UTR and the distribution of a particular miRNA among AGO proteins expressed in the cell. These considerations warrant further investigation to more clearly define the regulation of miRNA-dependent silencing.

We may speculate that, for our miR-99/100 reporter, in the presence of LIMD1, the majority of targeted sites on the reporter mRNA are occupied by AGO2-LIMD1 with a minor contribution of AGO3-WTIP. Potential interactions between LIMD1 and WTIP may be required for overall stability of the multiple RISCs assembled on this 3′ UTR, and short-term loss of either one (in the context of siRNA knockdown) would lead to significant de-repression of silencing. With LIMD1 loss, AGO3-WTIP may increase in occupancy of remaining/free sites on the reporter thereby rendering silencing highly dependent on AGO3.

Loss of LIMD1 by genetic ablation did not affect silencing of the *let-7a* reporter, but the underlying AGO utilization was altered, with AGO2 no longer contributing to silencing (Figures S1E and S1F). In this respect, as seen with the miR-99/100 reporter, long-term loss of LIMD1 led to functional adaptation to maintain miRNA silencing and also revealed the particular involvement of LIMD1 in AGO2-dependent miRNA silencing. The *let-7a* reporter contained six tandem *let-7a* sites, and its repression was dependent on AGO1, 2, and 3. This result suggests that, for this particular synthetic 3′ UTR, a broad cooperative alliance of AGO1–3 and LIM domain proteins is necessary for silencing and that short-term loss of any component by siRNA knockdown is highly unfavorable.

Post-transcriptional gene silencing involving miRNAs was first discovered over 20 years ago but only now are its precise mechanism and place within the array of cellular signaling networks becoming more clearly defined. There is now intense interest in how post-translational modification of components of miRNA biogenesis and miRISC can regulate and fine-tune both siRNA and miRNA-mediated gene silencing. A particularly active topic in the field is how phosphorylation of miRISC proteins can affect miRNA loading and recruitment to miRISC, ultimately affecting functionality (Golden et al., 2017; Horman et al., 2013; Lopez-Orozco et al., 2015; McKenzie et al., 2016; Rüdel et al., 2011; Shen et al., 2013; Warner et al., 2016; Yang et al., 2014; Zeng et al., 2008). Our findings identify that phosphorylation causes AGO selection for silencing by recruitment of a LIMD1 family member and sequential assembly of AGO-TNRC6A miRISC.

EXPERIMENTAL PROCEDURES

Cell Culture

HEK293T, HeLa, and U2OS cells were routinely cultured in DMEM (Sigma) supplemented with 10% fetal calf serum (FCS), 100 U/mL penicillin, and 100 μg/mL streptomycin. Akt inhibitor MK-2206 was used at a final concentration of 10 μM in complete DMEM, for 16 hr.

Plasmids

The generation of constructs used in this study are detailed in the Supplemental Experimental Procedures.

Luciferase Reporter Assays

Firefly and Renilla luciferase activities were assayed with the Dual-Luciferase Reporter Assay System (Promega) according to the manufacturer's instructions. Further details are provided in the Supplemental Experimental Procedures.

Immunoprecipitation and Western Blotting

Routine methods to immunoprecipitate proteins were employed and detailed in the Supplemental Experimental Procedures. Antibodies used in the study are detailed in Table S2.

Expression of Recombinant Proteins and Purification

Details are provided in Supplemental Experimental Procedures.

MBP Direct Binding Assay

Details are provided in Supplemental Experimental Procedures.

GST-Pull-Down Assays

Details are provided in Supplemental Experimental Procedures.

Real-Time qPCR

Details are provided in Supplemental Experimental Procedures.

Lentiviral Line Generation

To obtain pseudotyped lentivirus (recombinant HIV-1 with vesicular stomatitis virus G [VSV-G] envelope protein), we used the gene delivery and production system developed by Naldini et al. (1996). Details for selection are in the Supplemental Experimental Procedures.

Protein Mass Spectrometry

Details are provided in Supplemental Experimental Procedures.

IF Microscopy

For analysis of miRISC protein localization, cells were fixed with 4% paraformaldehyde and stained according to standard procedures, which are detailed in the Supplemental Experimental Procedures.

PLA

PLAs were performed on HeLa cells prepared as for IF assays, including primary antibody incubation. Subsequently, Duolink In Situ PLA probes (1:10 in 2% BSA/0.025% Tween PBS) and orange detection reagents were used in accordance with the manufacturer's instructions. For negative controls, each of the primary antibodies was added with an immunoglobulin G (IgG) antibody for the corresponding antibody species (rabbit, mouse, or goat, as appropriate). PLA signal in images has been digitally intensified for visual clarity in printed figures.

CRISPR-Cas9 Cell Line Generation

LIMD1 knockout CRISPR cell lines (HeLa) were generated using the lenti-CRISPR v2 plasmid (Sanjana et al., 2014), acquired from Addgene (plasmid 52961). Further details for guide RNA sequence and clonal selection are in the Supplemental Experimental Procedures.

Quantification and Statistical Analysis

PLA interaction events were counted using the ImageJ "Analyze Particles" function. Data were stratified according to quartiles for the control condition and displayed as a stacked histogram. Statistical significance was calculated using chi-square analysis (for grouped data).

Quantification of western blots was performed by densitometric analysis using ImageJ software. For quantification of relative binding in co-immunoprecipitation assays, co-immunoprecipitated proteins were double normalized first against their input levels and second against levels of immunoprecipitated protein species. Binding data displayed are relative to WT or untreated conditions.

Statistical significance was calculated using the Student's t test, unless otherwise specified. Significance is represented as *$p < 0.05$, **$p < 0.001$, or ***$p < 0.0001$ throughout.

SUPPLEMENTAL INFORMATION

Supplemental Information includes Supplemental Experimental Procedures, seven figures, and two tables and can be found with this article online at http://dx.doi.org/10.1016/j.celrep.2017.06.027.

AUTHOR CONTRIBUTIONS

K.S.B., K.M.S., Y.L., S.C.K.W., D.E.F., D.C.M., M.R.H., M.J.P., D.L., and T.V.S. designed and performed experiments and analyzed the data. K.M.D., J.G.F., P.R.G., K.Y., R.R., P.S.R., X.W., A.A.A., and M.J.P. provided reagents, experimental advice, and design. All authors contributed to editing and proofreading the manuscript. K.S.B., K.M.S., M.J.P., D.L., and T.V.S. wrote the manuscript. T.V.S. supervised and managed all research.

ACKNOWLEDGMENTS

We thank Prof Ian MacRae and Jessica Sheu-Gruttadauria from The Scripps Research Institute, San Diego for providing purified recombinant AGO2 protein. We also thank Dr. Incheol Shin from Hanyang University in South Korea for providing the Myristoylated-Akt3 construct. K.S.B. is supported by funds from the BBSRC (Grant Code BB/L027755/1). K.M.S. was supported by funds from the BBSRC (Grant Code BB/I007571/1). J.G.F. was supported by funds from CRUK (C8932/A12733). M.J.P. is a BBSRC New Investigator (BB/N018818/1). D.L. is a Medical Research Council New Investigator Research Grant holder (MR/L008505/1) with further funding from The Wellcome Trust through the Centre for Chronic Diseases and Disorders at the University of York (097829). This work was supported by funds awarded to T.V.S. from the BBSRC (Grant Code BB/I007571/1).

Received: December 11, 2015
Revised: April 27, 2017
Accepted: June 9, 2017
Published: July 5, 2017

REFERENCES

Bartel, D.P. (2004). MicroRNAs: Genomics, biogenesis, mechanism, and function. Cell *116*, 281–297.

Bazzini, A.A., Lee, M.T., and Giraldez, A.J. (2012). Ribosome profiling shows that miR-430 reduces translation before causing mRNA decay in zebrafish. Science *336*, 233–237.

Behm-Ansmant, I., Rehwinkel, J., Doerks, T., Stark, A., Bork, P., and Izaurralde, E. (2006). mRNA degradation by miRNAs and GW182 requires both CCR4:NOT deadenylase and DCP1:DCP2 decapping complexes. Genes Dev. *20*, 1885–1898.

Chekulaeva, M., Mathys, H., Zipprich, J.T., Attig, J., Colic, M., Parker, R., and Filipowicz, W. (2011). miRNA repression involves GW182-mediated recruitment of CCR4-NOT through conserved W-containing motifs. Nat. Struct. Mol. Biol. *18*, 1218–1226.

Chen, Y., Boland, A., Kuzuoğlu-Öztürk, D., Bawankar, P., Loh, B., Chang, C.T., Weichenrieder, O., and Izaurralde, E. (2014). A DDX6-CNOT1 complex and W-binding pockets in CNOT9 reveal direct links between miRNA target recognition and silencing. Mol. Cell *54*, 737–750.

Chu, C.Y., and Rana, T.M. (2006). Translation repression in human cells by microRNA-induced gene silencing requires RCK/p54. PLoS Biol. *4*, e210.

De, N., and Macrae, I.J. (2011). Purification and assembly of human Argonaute, Dicer, and TRBP complexes. Methods Mol. Biol. *725*, 107–119.

Dueck, A., Ziegler, C., Eichner, A., Berezikov, E., and Meister, G. (2012). microRNAs associated with the different human Argonaute proteins. Nucleic Acids Res. *40*, 9850–9862.

Editorial. (2009). Beyond the diffraction limit. Nat. Photon *3*, 361–361.

Elkayam, E., Kuhn, C.D., Tocilj, A., Haase, A.D., Greene, E.M., Hannon, G.J., and Joshua-Tor, L. (2012). The structure of human argonaute-2 in complex with miR-20a. Cell *150*, 100–110.

Eulalio, A., Behm-Ansmant, I., Schweizer, D., and Izaurralde, E. (2007). P-body formation is a consequence, not the cause, of RNA-mediated gene silencing. Mol. Cell. Biol. *27*, 3970–3981.

Eulalio, A., Helms, S., Fritzsch, C., Fauser, M., and Izaurralde, E. (2009a). A C-terminal silencing domain in GW182 is essential for miRNA function. RNA *15*, 1067–1077.

Eulalio, A., Tritschler, F., and Izaurralde, E. (2009b). The GW182 protein family in animal cells: New insights into domains required for miRNA-mediated gene silencing. RNA *15*, 1433–1442.

Förstemann, K., Horwich, M.D., Wee, L., Tomari, Y., and Zamore, P.D. (2007). Drosophila microRNAs are sorted into functionally distinct argonaute complexes after production by dicer-1. Cell *130*, 287–297.

Golden, R.J., Chen, B., Li, T., Braun, J., Manjunath, H., Chen, X., Wu, J., Schmid, V., Chang, T.C., Kopp, F., et al. (2017). An Argonaute phosphorylation cycle promotes microRNA-mediated silencing. Nature *542*, 197–202.

Horman, S.R., Janas, M.M., Litterst, C., Wang, B., MacRae, I.J., Sever, M.J., Morrissey, D.V., Graves, P., Luo, B., Umesalma, S., et al. (2013). Akt-mediated phosphorylation of argonaute 2 downregulates cleavage and upregulates translational repression of MicroRNA targets. Mol. Cell *50*, 356–367.

Huntzinger, E., and Izaurralde, E. (2011). Gene silencing by microRNAs: Contributions of translational repression and mRNA decay. Nat. Rev. Genet. *12*, 99–110.

Hutvagner, G., and Simard, M.J. (2008). Argonaute proteins: Key players in RNA silencing. Nat. Rev. Mol. Cell Biol. *9*, 22–32.

James, V., Zhang, Y., Foxler, D.E., de Moor, C.H., Kong, Y.W., Webb, T.M., Self, T.J., Feng, Y., Lagos, D., Chu, C.Y., et al. (2010). LIM-domain proteins, LIMD1, Ajuba, and WTIP are required for microRNA-mediated gene silencing. Proc. Natl. Acad. Sci. USA *107*, 12499–12504.

Jee, D., and Lai, E.C. (2014). Alteration of miRNA activity via context-specific modifications of Argonaute proteins. Trends Cell Biol. *24*, 546–553.

Koch, B.J., Ryan, J.F., and Baxevanis, A.D. (2012). The diversification of the LIM superclass at the base of the metazoa increased subcellular complexity and promoted multicellular specialization. PLoS ONE *7*, e33261.

La Rocca, G., Olejniczak, S.H., González, A.J., Briskin, D., Vidigal, J.A., Spraggon, L., DeMatteo, R.G., Radler, M.R., Lindsten, T., Ventura, A., et al. (2015). In vivo, Argonaute-bound microRNAs exist predominantly in a reservoir of low molecular weight complexes not associated with mRNA. Proc. Natl. Acad. Sci. USA *112*, 767–772.

Lian, S.L., Li, S., Abadal, G.X., Pauley, B.A., Fritzler, M.J., and Chan, E.K. (2009). The C-terminal half of human Ago2 binds to multiple GW-rich regions of GW182 and requires GW182 to mediate silencing. RNA *15*, 804–813.

Liu, J., Rivas, F.V., Wohlschlegel, J., Yates, J.R., 3rd, Parker, R., and Hannon, G.J. (2005). A role for the P-body component GW182 in microRNA function. Nat. Cell Biol. *7*, 1261–1266.

Lopez-Orozco, J., Pare, J.M., Holme, A.L., Chaulk, S.G., Fahlman, R.P., and Hobman, T.C. (2015). Functional analyses of phosphorylation events in human Argonaute 2. RNA *21*, 2030–2038.

Mathys, H., Basquin, J., Ozgur, S., Czarnocki-Cieciura, M., Bonneau, F., Aartse, A., Dziembowski, A., Nowotny, M., Conti, E., and Filipowicz, W. (2014). Structural and biochemical insights to the role of the CCR4-NOT complex and DDX6 ATPase in microRNA repression. Mol. Cell *54*, 751–765.

McKenzie, A.J., Hoshino, D., Hong, N.H., Cha, D.J., Franklin, J.L., Coffey, R.J., Patton, J.G., and Weaver, A.M. (2016). KRAS-MEK Signaling Controls Ago2 Sorting into Exosomes. Cell Rep. *15*, 978–987.

Naldini, L., Blömer, U., Gallay, P., Ory, D., Mulligan, R., Gage, F.H., Verma, I.M., and Trono, D. (1996). In vivo gene delivery and stable transduction of nondividing cells by a lentiviral vector. Science *272*, 263–267.

Patranabis, S., and Bhattacharyya, S.N. (2016). Phosphorylation of Ago2 and subsequent inactivation of let-7a RNP-specific MicroRNAs control differentiation of mammalian sympathetic neurons. Mol. Cell. Biol. *36*, 1260–1271.

Petri, S., Dueck, A., Lehmann, G., Putz, N., Rüdel, S., Kremmer, E., and Meister, G. (2011). Increased siRNA duplex stability correlates with reduced off-target and elevated on-target effects. RNA *17*, 737–749.

Pfaff, J., Hennig, J., Herzog, F., Aebersold, R., Sattler, M., Niessing, D., and Meister, G. (2013). Structural features of Argonaute-GW182 protein interactions. Proc. Natl. Acad. Sci. USA *110*, E3770–E3779.

Rouya, C., Siddiqui, N., Morita, M., Duchaine, T.F., Fabian, M.R., and Sonenberg, N. (2014). Human DDX6 effects miRNA-mediated gene silencing via direct binding to CNOT1. RNA *20*, 1398–1409.

Rüdel, S., Wang, Y., Lenobel, R., Körner, R., Hsiao, H.H., Urlaub, H., Patel, D., and Meister, G. (2011). Phosphorylation of human Argonaute proteins affects small RNA binding. Nucleic Acids Res. *39*, 2330–2343.

Sahin, U., Lapaquette, P., Andrieux, A., Faure, G., and Dejean, A. (2014). Sumoylation of human argonaute 2 at lysine-402 regulates its stability. PLoS ONE *9*, e102957.

Sanjana, N.E., Shalem, O., and Zhang, F. (2014). Improved vectors and genome-wide libraries for CRISPR screening. Nat Methods. *11*, 783–784.

Schirle, N.T., and MacRae, I.J. (2012). The crystal structure of human Argonaute2. Science *336*, 1037–1040.

Shen, J., Xia, W., Khotskaya, Y.B., Huo, L., Nakanishi, K., Lim, S.O., Du, Y., Wang, Y., Chang, W.C., Chen, C.H., et al. (2013). EGFR modulates microRNA maturation in response to hypoxia through phosphorylation of AGO2. Nature *497*, 383–387.

Söderberg, O., Gullberg, M., Jarvius, M., Ridderstråle, K., Leuchowius, K.J., Jarvius, J., Wester, K., Hydbring, P., Bahram, F., Larsson, L.G., and Landegren, U. (2006). Direct observation of individual endogenous protein complexes in situ by proximity ligation. Nat. Methods *3*, 995–1000.

Song, J., Park, S., Kim, M., and Shin, I. (2008). Down-regulation of Notch-dependent transcription by Akt in vitro. FEBS Lett. *582*, 1693–1699.

Su, H., Trombly, M.I., Chen, J., and Wang, X. (2009). Essential and overlapping functions for mammalian Argonautes in microRNA silencing. Genes Dev. *23*, 304–317.

Takimoto, K., Wakiyama, M., and Yokoyama, S. (2009). Mammalian GW182 contains multiple Argonaute-binding sites and functions in microRNA-mediated translational repression. RNA *15*, 1078–1089.

Tomari, Y., Du, T., and Zamore, P.D. (2007). Sorting of Drosophila small silencing RNAs. Cell *130*, 299–308.

Valdmanis, P.N., Gu, S., Schüermann, N., Sethupathy, P., Grimm, D., and Kay, M.A. (2012). Expression determinants of mammalian argonaute proteins in mediating gene silencing. Nucleic Acids Res. *40*, 3704–3713.

Wang, D., Zhang, Z., O'Loughlin, E., Lee, T., Houel, S., O'Carroll, D., Tarakhovsky, A., Ahn, N.G., and Yi, R. (2012). Quantitative functions of Argonaute proteins in mammalian development. Genes Dev. *26*, 693–704.

Warner, M.J., Bridge, K.S., Hewitson, J.P., Hodgkinson, M.R., Heyam, A., Massa, B.C., Haslam, J.C., Chatzifrangkeskou, M., Evans, G.J., Plevin, M.J., et al. (2016). S6K2-mediated regulation of TRBP as a determinant of miRNA expression in human primary lymphatic endothelial cells. Nucleic Acids Res. *44*, 9942–9955.

Yang, M., Haase, A.D., Huang, F.K., Coulis, G., Rivera, K.D., Dickinson, B.C., Chang, C.J., Pappin, D.J., Neubert, T.A., Hannon, G.J., et al. (2014). Dephosphorylation of tyrosine 393 in argonaute 2 by protein tyrosine phosphatase 1B regulates gene silencing in oncogenic RAS-induced senescence. Mol. Cell *55*, 782–790.

Zeng, Y., Sankala, H., Zhang, X., and Graves, P.R. (2008). Phosphorylation of Argonaute 2 at serine-387 facilitates its localization to processing bodies. Biochem. J. *413*, 429–436.

Cell Reports, Volume *20*

Supplemental Information

Argonaute Utilization for miRNA Silencing Is Determined by Phosphorylation-Dependent Recruitment of LIM-Domain-Containing Proteins

Katherine S. Bridge, Kunal M. Shah, Yigen Li, Daniel E. Foxler, Sybil C.K. Wong, Duncan C. Miller, Kathryn M. Davidson, John G. Foster, Ruth Rose, Michael R. Hodgkinson, Paulo S. Ribeiro, A. Aziz Aboobaker, Kenta Yashiro, Xiaozhong Wang, Paul R. Graves, Michael J. Plevin, Dimitris Lagos, and Tyson V. Sharp

Figure S1.

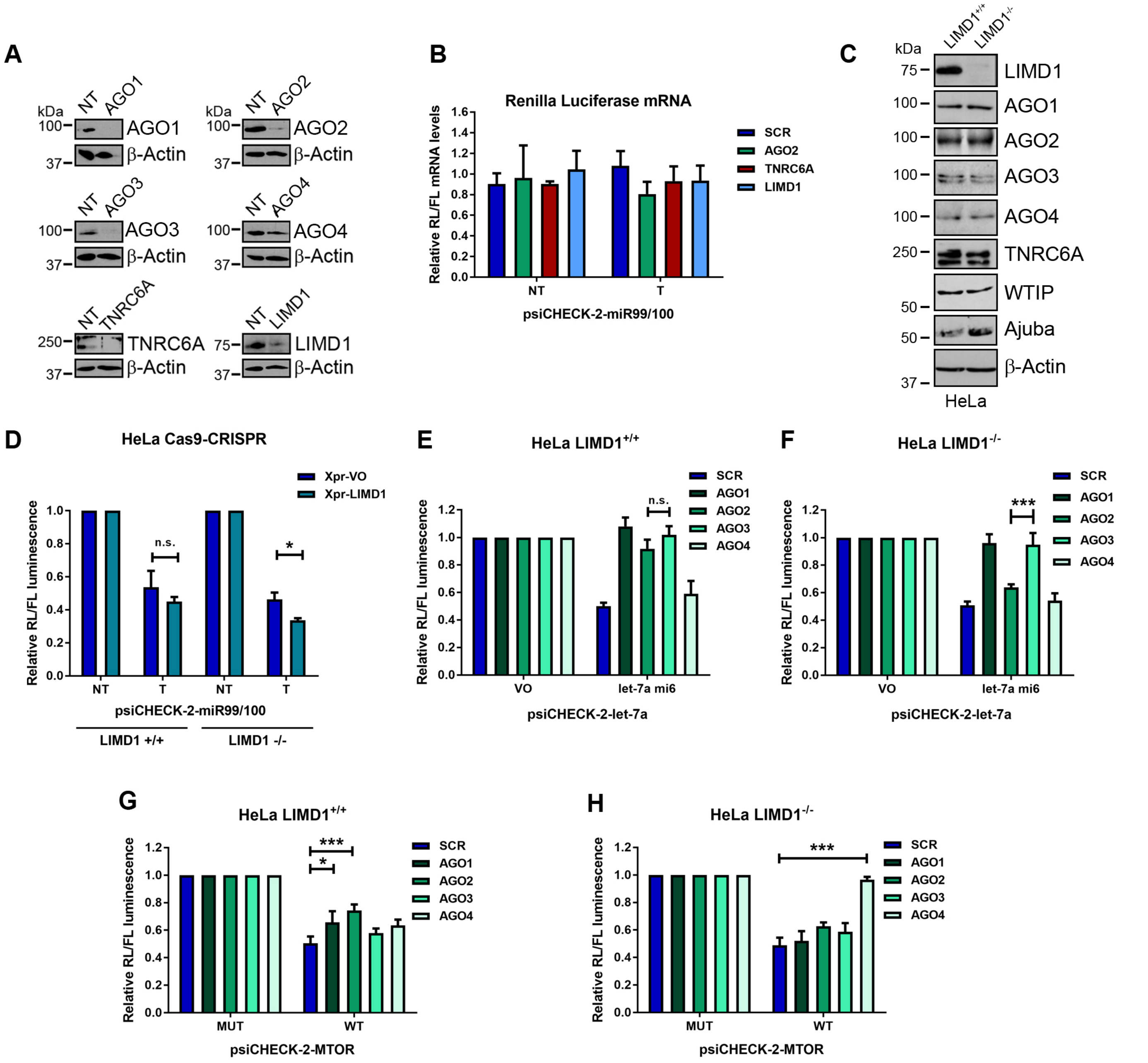

Figure S1. Related to Figure 1. Genetic ablation of LIMD1 switches AGO dependency within miRNA silencing. **(A)** Western blot analysis of siRNA-mediated knockdown of the indicated proteins of interest in HeLa cells. β-Actin loading control. **(B)** Quantitative real-time PCR (qRT-PCR) of Renilla Luciferase (RL) mRNA levels, normalised to Firefly Luciferase (FL) mRNA in HeLa cells. **(C)** Western blot analysis of the indicated proteins in CRISPR-Cas9 gene-edited HeLa cells; LIMD1 +/+ (Cas9 alone control) or LIMD1 -/-. **(D)** psiCHECK-2-miR-99/100 reporter assay in CRISPR-Cas9 gene-edited HeLa transfected with Xpress (Xpr)-tagged vector only (VO) or LIMD1. **(E)** psiCHECK-2-*let-7a* (non-targeting [NT], targeting [T]) reporter in CRISPR-Cas9 gene-edited HeLa cells with Cas9 alone control ($LIMD1^{+/+}$) or **(F)** LIMD1 knockout ($LIMD1^{-/-}$) cells, treated with the indicated siRNAs. **(G)** psiCHECK-2-MTOR (endogenous mutant [MUT] or wild type [WT] 3'-UTR) reporter in CRISPR-Cas9 gene-edited HeLa cells with Cas9 alone control ($LIMD1^{+/+}$) or **(H)** LIMD1 knockout ($LIMD1^{-/-}$) cells, treated with the indicated siRNAs. Unless otherwise stated, data shown are mean ±SEM, n=3,* $p<0.05$, ** $p<0.001$, *** $p<0.0001$.

Figure S2.

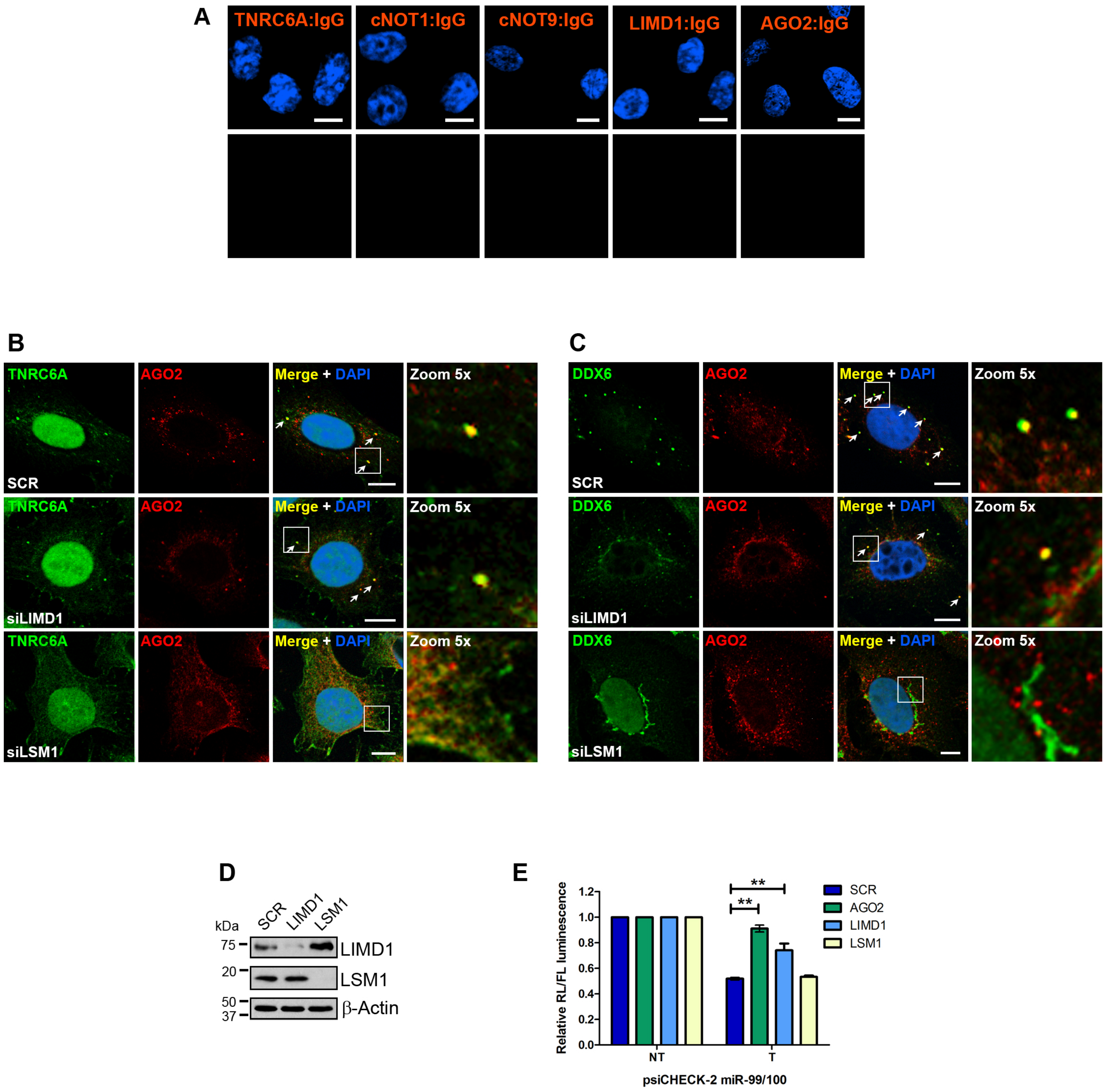

Figure S2. Related to Figure 2. Loss of P-body component LSM1 does not affect AGO2 interaction with TNRC6A/DDX6 or miRNA silencing activity.

(A) Negative control for endogenous *in situ* interaction determined by proximity ligation assay (PLA) of the indicated proteins. Cells stained with DAPI (top). **(B)** Endogenous immunofluorescence analysis of AGO2 with TNRC6A or **(C)** DDX6 in HeLa cells treated with the indicated siRNAs (SCR, non-targeting control). **(D)** Western blot analysis of HeLa cells in (B) and (C). **(E)** psiCHECK-2-miR-99/100 (NT [non-targeting], T [targeting]) reporter assay in HeLa cells treated with the indicated siRNAs. Data shown are mean ±SEM, n=3,* p<0.05, ** p<0.001, *** p<0.0001. Scale bars, 10 µm.

Figure S3.

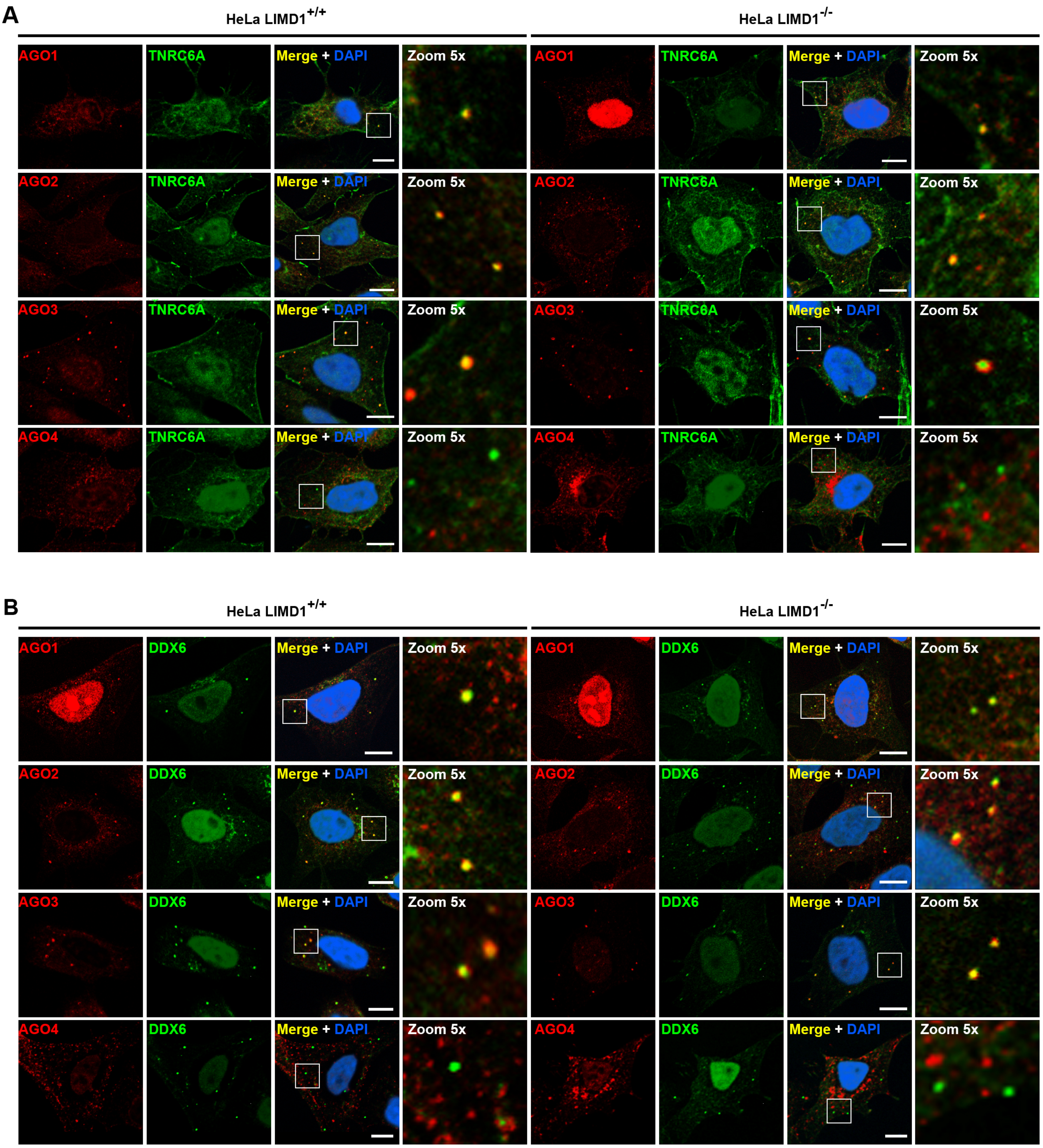

Figure S3. Related to Figure 2. Immunofluorescent colocalization of miRISC components is not a definitive indication of direct interactions.

(A) Endogenous immunofluorescence analysis in HeLa CRISPR LIMD1$^{+/+}$ and LIMD1$^{-/-}$ cells of AGO1, 2, 3 and 4 with TNRC6A and **(B)** DDX6. Scale bars, 10µm.

Figure S4.

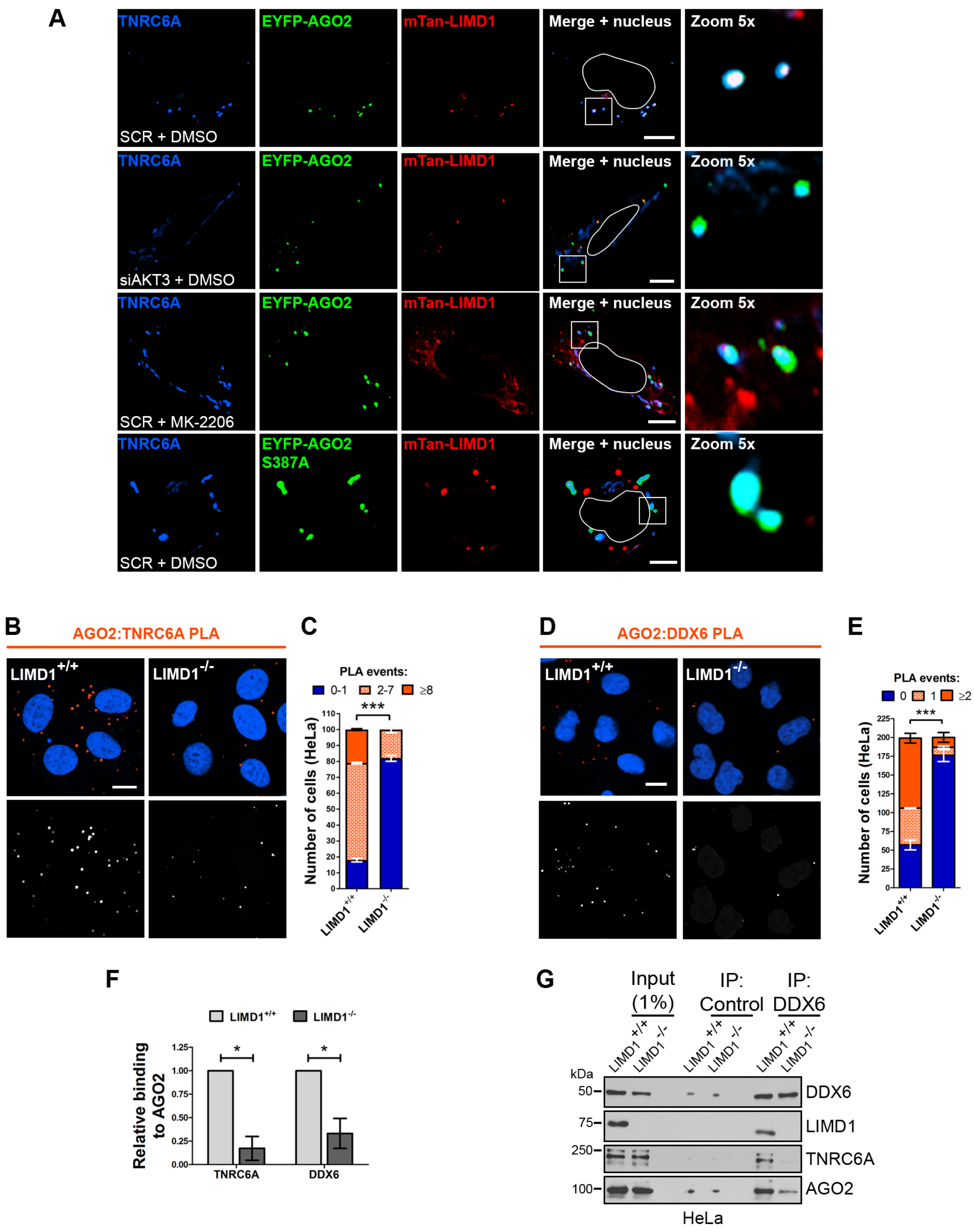

Figure S4. Related to Figure 2. Loss of LIMD1 significantly impairs AGO2 interaction with miRISC components TNRC6A and DDX6

A) Immunofluorescence of endogenous TNRC6A with EYFP-AGO2 or -AGO2 S387A and mTan-LIMD1. Cells treated with the indicated siRNAs (SCR, non-targeting control) or pan Akt-inhibitor (MK-2206). Scale bars, 10µm. **(B)** PLA analysis of endogenous AGO2 and TNRC6A interaction in CRISPR-Cas9 gene-edited HeLa cells. PLA signal orange, cells stained with DAPI (top); PLA signal white for visual clarity (bottom). **(C)** Quantification of PLA interaction events in (B), displayed as a stacked histogram. Data shown is mean ±SEM, n=3, total of 100 cells. ** $p<0.001$, *** $p<0.0001$, determined using the Chi-squared test. Scale bars, 10µm. **(D)** PLA analysis of AGO2:DDX6 interaction in above CRISPR-Cas9 gene-edited HeLa cell lines. **(E)** Quantification of (D) as in (C). **(F)** Quantification of relative binding of TNRC6A and DDX6 to AGO2 from CRISPR-Cas9 gene-edited HeLa LIMD1$^{+/+}$ or LIMD1$^{-/-}$ cells. Data shown is mean ±SEM, n=3, * $p<0.05$, determined using the Student's t-test **(G)** Immunoprecipiration of DDX6 from HeLa LIMD1$^{+/+}$ or LIMD1$^{-/-}$, analysed for the indicated proteins by western blot.

Figure S5.

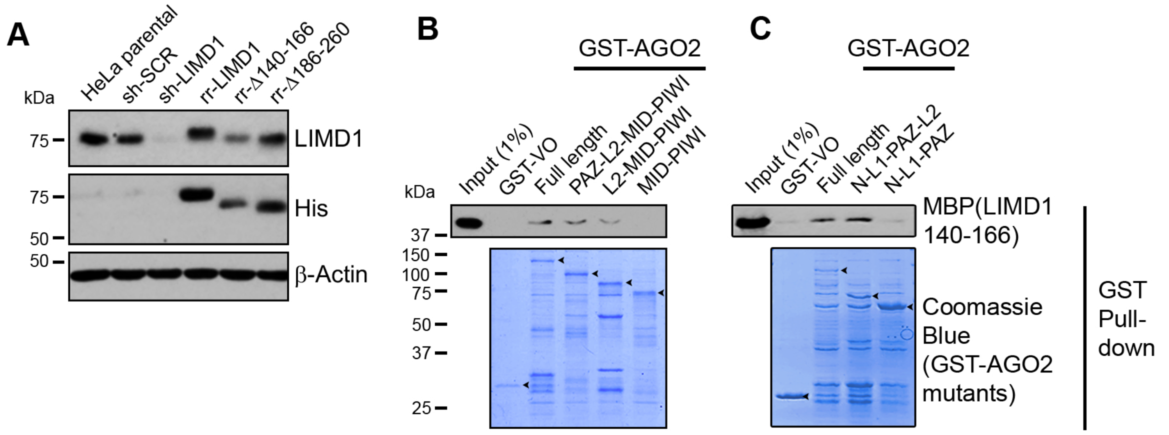

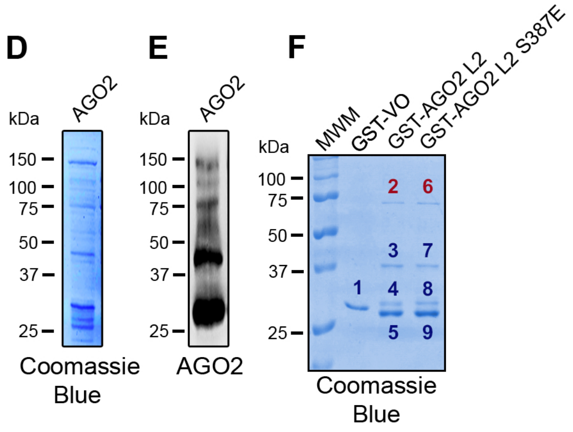

G

Band	Protein ID	# peptides	Score	Peptide sequences
1	GST	6	563	LTQSMAIIR<br>AEISMLEGAVLDIR<br>ERAEISMLEGAVLDIR<br>LLLEYLEEKYEEHLYER<br>YIAWPLQGWQATFGGGDHPPK<br>KFELGLEFPNLPYYIDGDVK
2	*E. Coli* Chaperone protein DNA K	5	626	TAEDYLGEPVTEAVITVPAYFNDAQR<br>AKLESLVEDLVNR<br>VALQDAGLSVSDIDDVILVGGQTR<br>HSQVFSTAEDNQSAVTIHVLQGER<br>SLGQFNLDGINPAPR
3	GST	4	427	AEISMLEGAVLDIR<br>ERAEISMLEGAVLDIR<br>LLLEYLEEKYEEHLYER<br>YIAWPLQGWQATFGGGDHPPK
	AGO2	3	289	SASFNTDPYVR<br>VLQPPSILYGGR<br>AIATPVQGVWDMR
4	GST	6	624	YEEHLYER<br>AEISMLEGAVLDIR<br>ERAEISMLEGAVLDIR<br>LLLEYLEEKYEEHLYER<br>YIAWPLQGWQATFGGGDHPPK<br>KFELGLEFPNLPYYIDGDVK
5	GST	6	565	LTQSMAIIR<br>AEISMLEGAVLDIR<br>ERAEISMLEGAVLDIR<br>LLLEYLEEKYEEHLYER<br>YIAWPLQGWQATFGGGDHPPK<br>KFELGLEFPNLPYYIDGDVK
6	*E. Coli* Chaperone protein DNA K	9	928	TAEDYLGEPVTEAVITVPAYFNDAQR<br>LINYLVEEFKK<br>AKLESLVEDLVNR<br>LESLVEDLVNR<br>VALQDAGLSVSDIDDVILVGGQTR<br>HSQVFSTAEDNQSAVTIHVLQGER<br>SLGQFNLDGINPAPR<br>KFEELVQTR<br>NQGDHLLHSTR
7	GST	5	477	YEEHLYER<br>AEISMLEGAVLDIR<br>ERAEISMLEGAVLDIR<br>LLLEYLEEKYEEHLYER<br>YIAWPLQGWQATFGGGDHPPK
	AGO2	2	180	VLQPPSILYGGR<br>AIATPVQGVWDMR
8	GST	6	543	LTQSMAIIR<br>AEISMLEGAVLDIR<br>ERAEISMLEGAVLDIR<br>LLLEYLEEKYEEHLYER<br>YIAWPLQGWQATFGGGDHPPK<br>KFELGLEFPNLPYYIDGDVK
9	GST	5	579	AEISMLEGAVLDIR<br>ERAEISMLEGAVLDIR<br>LLLEYLEEKYEEHLYER<br>YIAWPLQGWQATFGGGDHPPK<br>KFELGLEFPNLPYYIDGDVK

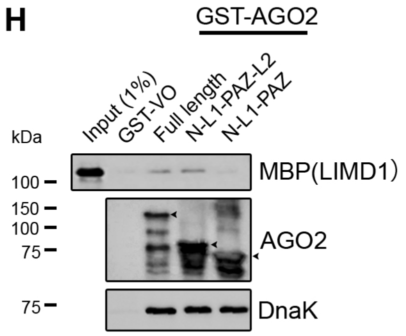

Figure S5. Related to Figure 3. LIMD1 AB-motif (a.a. 140-166) directly binds AGO2 L2 domain.
(A) sh-RNA mediated knock-down and rescue of RNAi resistant (rr) LIMD1 or deletion mutants, HeLa cell lines. sh-SCR is non-targeting control. **(B)** Direct binding assay of N-terminal and **(C)** C-terminal truncation mutants of GST-AGO2 with MBP-LIMD1 140-166 (ABD). **(D)** Purified GST-AGO2 analysed by Coomassie Blue staining and **(E)** western blot analysis for AGO2. **(F)** Degradation products of GST-AGO2 L2/ L2 S387E were identified by **(G)** mass spectrometry analysis. **(H)** DnaK identified as present in purified GST-AGO2 samples did not affect binding to LIMD1.

Figure S6.

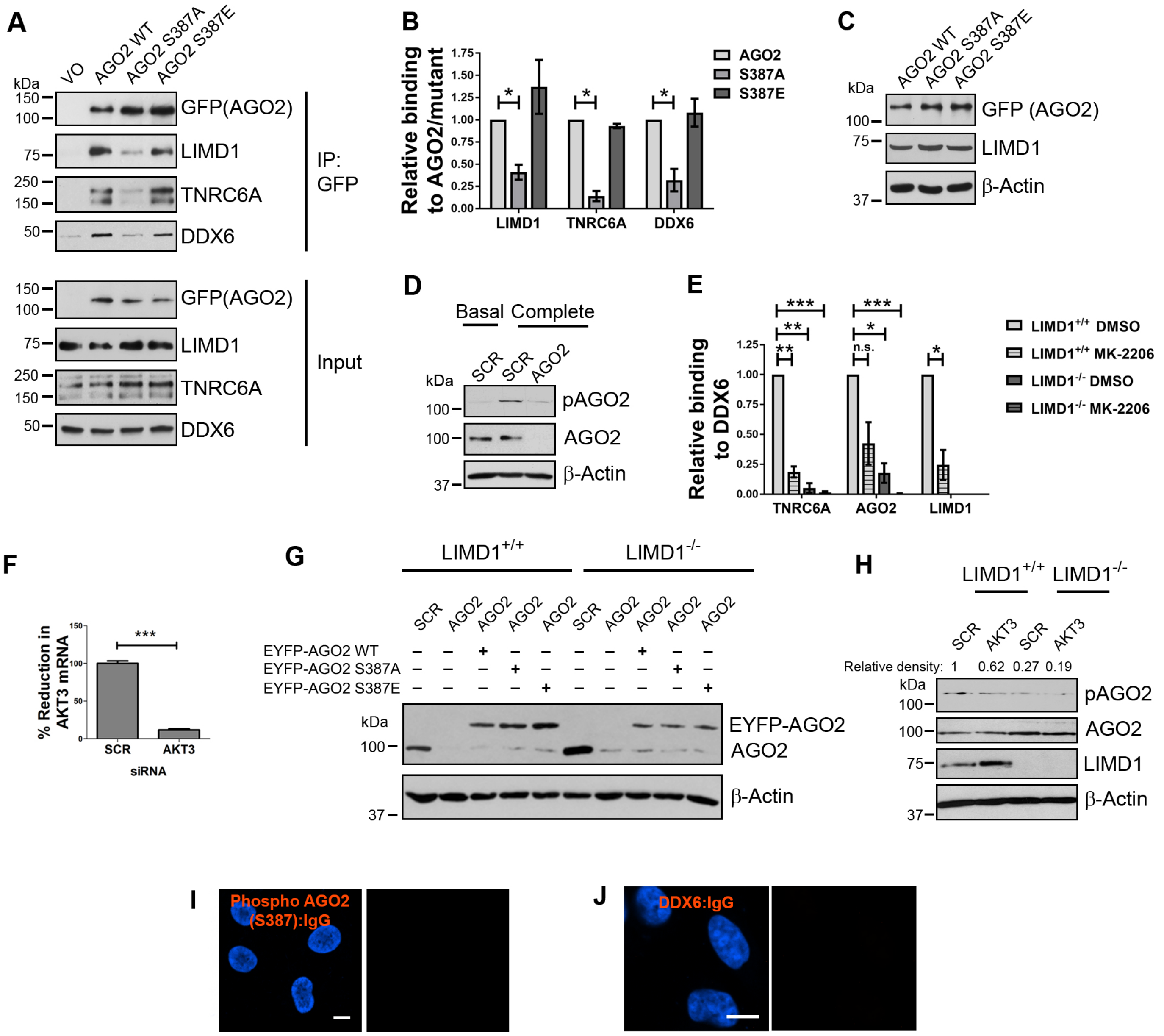

Figure S6. Related to Figures 4 and 5. Akt3-mediated phosphorylation of AGO2 S387 directs interaction with LIMD1 and recruitment of miRISC components TNRC6A and DDX6.

(A) Immunoprecipiration of GFP-VO, AGO2 and S387A/S387E point mutants from HEK293T lysate co-transfected with Xpr-LIMD1, analysed for the indicated proteins by western blot. **(B)** Quantification of co-IP in (A), data shown is mean ±SEM, n=4. **(C)** Western blot analysis of indicated overexpressed proteins in U2OS cells used for immunofluorescence analysis. β-Actin loading control. **(D)** Western blot analysis of phospho-AGO2 (S387) antibody specificity, in serum starved (basal) and complete medium conditions, treated with the indicated siRNAs. **(E)** Quantification of TNRC6A, AGO2 and LIMD1 interaction with DDX6 immunoprecipitated from HeLa $LIMD1^{+/+}$ or $LIMD1^{-/-}$ cells treated with DMSO or MK-2206. **(F)** qRT-PCR analysis of AKT3 mRNA in U2OS upon treatment with non-targeting (SCR) or AKT3 siRNA. **(G)** Western blot analysis of Cas9 control ($LIMD1^{+/+}$) and $LIMD1^{-/-}$ HeLa cell lines transfected with the indicated siRNAs and EYFP-VO/AGO2 constructs. AGO2 antibody detects both endogenous and EYFP-tagged AGO2. β-Actin loading control. **(H)** Western blot analysis of above HeLa CRISPR-Cas9 cell lines transfected with non-targeting (SCR) or AKT3 siRNA. Density of phospho-AGO2 (S387) band is calculated relative to SCR in the $LIMD1^{+/+}$ line. **(I)** Negative control PLA for the Phospho-S387-Ago2 antibody performed with goat IgG. PLA signal orange, cells stained with DAPI (top); PLA signal white for visual clarity (bottom). **(J)** Negative control PLA for the DDX6 antibody performed with rabbit IgG. Unless otherwise stated, data shown is mean ±SEM, n=3, * $p<0.05$, ** $p<0.01$, *** $p<0.001$, n.s. = not significant. Scale bars, 10μm.

Figure S7.

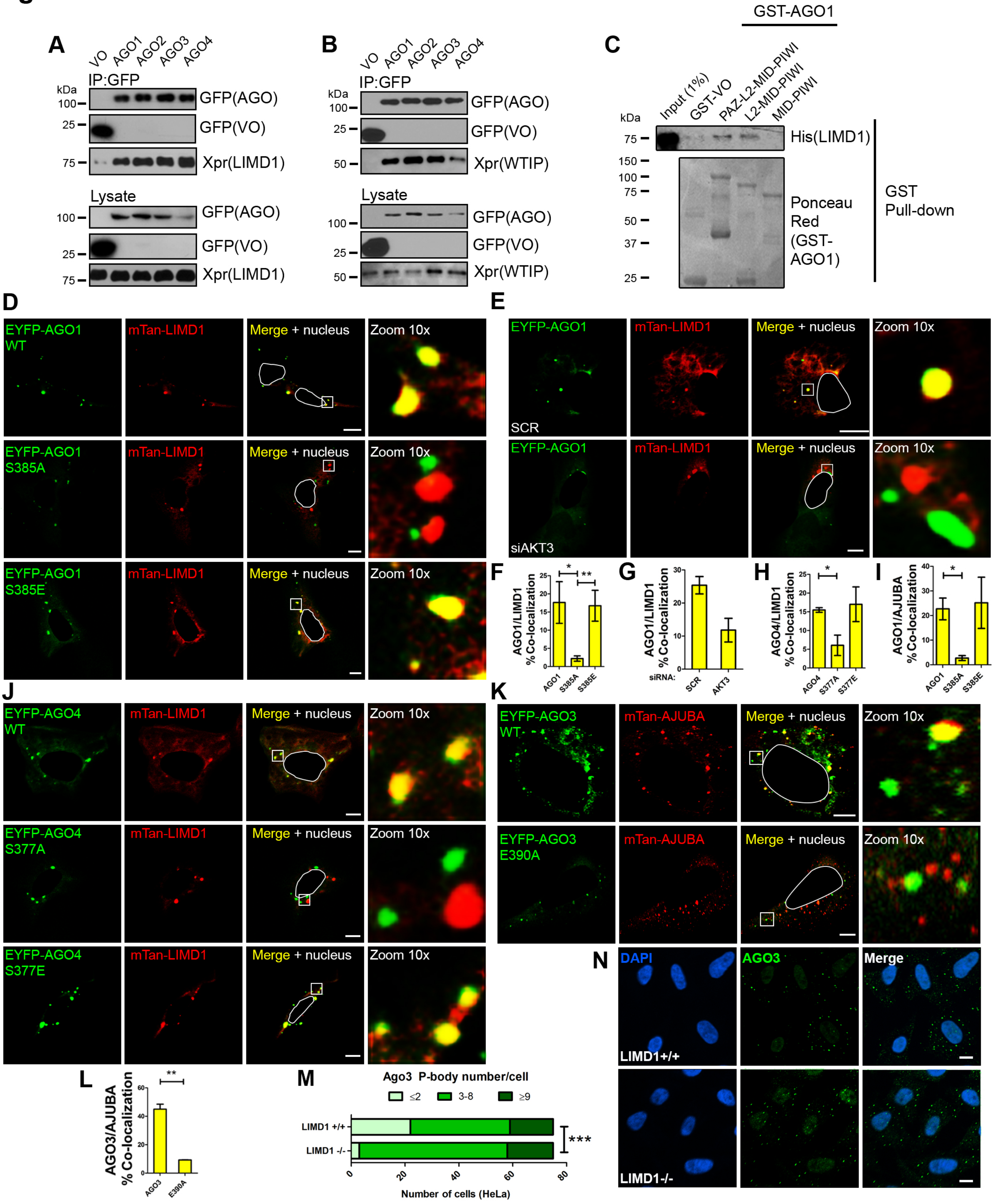

Figure S7. Related to Figures 6 and 7. AGO1-4 interact with LIMD1/Ajuba/WTIP in a phospho-dependent mechanism.

(A) Xpress-tagged (Xpr) LIMD1 co-immunoprecipitates with GFP-AGO1-4 from HEK293T lysate. **(B)** Xpr-WTIP co-IPs with GFP-AGO1-3 from HEK293T lysate. **(C)** The L2 domain of purified GST-AGO1 is required to directly bind His-LIMD1. **(D)** Co-localization of EYFP-AGO1/S385 point mutants with mTan-LIMD1 in U2OS cells. Nucleus outline in white. **(E)** Co-expression of EYFP-AGO1 with mTan-LIMD1 in U20S treated with non-targeting (SCR) or AKT3 siRNA. **(F)** Quantification of (D). **(G)** Quantification of (E). **(H)** Quantification of (J). **(I)** Quantification of Ago1/point mutant colocalization with AJUBA (images not shown). **(J)** Co-localization of EYFP-AGO4/S377 point mutants with mTan-LIMD1 in U2OS cells. **(K)** Co-expression of mTan-AJUBA with EYFP-AGO3 WT/E390 point mutant. **(L)** Quantification of (K). **(M)** Stacked histogram quantification of number of AGO3 P-bodies in (N). **(N)** Immunofluorescence staining for endogenous AGO3 in HeLa CRISPR-Cas9 cell lines. DAPI, nuclear staining. Unless otherwise stated, scale bars, 10μm. p<0.001, *** p<0.0001.

Supplemental Experimental Procedures

Luciferase Reporter Assays

For siRNA knockdown experiments, cells were reverse transfected in 96 well plates with siRNA at 40 nM using INTERFERin reagent (Polyplus transfection Illkirch, France). After 48h cells were transfected with 20 ng/well of psiCheck2 plasmid using Fugene 6 reagent or Viafect reagent (Promega). After a further 24h cells were harvested in 1x Passive Lysis buffer. Lysates were assayed for Firefly and Renilla luciferase activities with the Dual-Luciferase Reporter Assay System (Promega) according to the manufacturer’s instructions on the Wallac Victor2 1420 multilabel counter (Perkin-Elmer, Waltham, MA, USA).

For reporter assays without prior siRNA treatment, cells were plated in 96 well plates and 24h later transfected with the reporter plasmids as above. After 24h cells were harvested in 1x Passive Lysis buffer and assayed as above.

For reporter assays that included co-transfection of miRNA mimic, cells were transfected with reporter plasmids and miRNA mimic at 20 nM using JetPrime Reagent (Polyplus) and harvested 24h later in 1X Passive Lysis buffer.

Plasmids

Annealed oligonucleotides containing five tandem miR-99/100 sites with seed matches (T) or seed mismatches (NT) were cloned into the 3’ UTR region of the Renilla luciferase cassette in psiCheck2 (Promega, WI Madison USA) using the XhoI and PmeI restriction sites. Matched sites contained the sequence 5’AGCAAGTGTAACGG**TACGGGT**A-3’ (seed sequence in bold). Mismatched sites contained the sequence 5’AGCAAGTGTAACGG**TAATAAC**A-3’. For the artificial let-7a reporter six seed-matched let-7a sites were cloned into the XhoI and NotI sites of psiCheck2 5’-AACTATACAACGT**CTACCTC**A-3’ (seed sequence in bold). For FGFR3 and MTOR 3’UTR reporters, the 3’UTRs were PCR amplified using Phusion PCR mastermix using human genomic DNA as a template and primers containing XhoI and PmeI sites. PCR products were cloned into XhoI and PmeI sites of psiCheck2. miR-99/100 site mutants were

generated by site-directed mutagenesis using the Q5 site directed mutagenesis kit (New England Biolabs), according to the manufacturer's instructions. psiCheck2-TGFBR2 3'UTR WT and mutant plasmids were obtained from Addgene (31882 and 31883)

pFLRu *h*LIMD1 shRNA rrLIMD1-FH construct has previously been described (Foxler et al., 2012). Deletion mutants for the rrLIMD1 were made by site directed mutagenesis.

Ligation-independent cloning was carried out as described by Eschenfeldt *et al* (Eschenfeldt et al., 2009). Briefly, PCR products were separated on 1% agarose gels and purified. pMCSG7 or pMCSG10 vectors were linearized by digestion with SspI, and pETFPP_2 by BseRI (for cloning of MBP-LIMD1). Both PCR products and vectors were then treated with T4 DNA polymerase in the presence of the specific single deoxynucleotide. The transformation was followed by the usual protocol. Positive clones were identified with colony PCR, using T7 promoter and T7 terminator primers. Positive colonies were grown in 5 ml of LB media overnight and plasmids were prepared using a Plasmid Miniprep Kit (Roche).

Plasmids expressing mTan-LIMD1, mTan-WTIP and mTan-Ajuba have been described previously (James et al., 2010). pEYFP-C1-Ago1 and Ago2 were obtained from Dr. Tariq Rana (Chu and Rana, 2006) . Ago3 and Ago4 cDNAs were cloned into the EcoRI and XbaI sites of pEYFP-C1.

Expression of recombinant proteins and purification

Recombinant plasmids containing the target clones were transformed into Rosetta (DE3) expression strain. Single colonies from each plate were transferred into 5 ml of LB and incubated at 37°C, 250 rpm for 2.5 h until the optical density at 600 nm reached 0.6-0.8. IPTG was then added to reach a final concentration of 1 mmol/L to induce the protein expression at 25°C overnight. After overnight expression, the bacteria were collected and frozen on dry ice. The pellets were then thawed on ice and re-suspended in RIPA buffer, followed by sonication.

The supernatant was collected after centrifugation at 4°C for 15 min at 21,500 g and stored at -80°C for later use. For MBP-tagged proteins, a small amount of supernatant was incubated with 25 µl of His-Select Nickel Affinity Gel (SIGMA, P6611) and incubated at 4°C rotating for 30 min. The resin was then washed three times with His binding buffer (50 mM Tris-HCL, pH 8.0; 500 mM NaCl; 5 mM imidazole), followed by eluting in elution buffer (50 mM Tris-HCL, pH 8.0; 500 mM NaCl; 50 mM imidazole) by rotating at 4°C overnight. After overnight incubation, the supernatant was collected after centrifugation at 4°C for 1 min at 500 g and the imidazole was reduced by Amicon Ultra (Amicon, UFC501024). The purified lysates was then stored at -20°C for later use.

MBP direct binding assay

10ul of anti-MBP magnetic beads was washed once in PBS and incubated with cell lysates at 4 °C with rotation for 30 min to conjugate the MBP-tagged proteins onto the beads. The beads was then washed three times with 200µl RIPA with vortex and applied to magnet for 30s to remove the supernatant, followed by incubating with crystallography grade un-tagged AGO2 at 4 °C with rotation for 2h. After incubation, the beads were washed three times with 500µl binding buffer with vortex and the supernatant was removed. Proteins were finally eluted in 20µl 2xSDS-PAGE sample loading buffer and then analysed by Western Blot.

GST-pulldown assays

20µl of pre-blocked Glutathione Sepharose 4B resin (GE Healthcare, 17-0756-01) was blocked at 4°C with rotation for overnight. Bacterial cell lysates were incubated with the resin at 4°C with rotation for 30min. The resin was then washed three times with 200µl RIPA with gentle vortex and centrifugation at 4 °C for 1 min, 500 g. The supernatant was carefully removed. The pre-purified MBP-tagged protein in binding buffer (150 mM NaCl, 20 mM Tris (pH 8.0), 1 mM $MgCl_2$, 0.1 % (v/v) IGEPAL CA-630 and 10 % (v/v) glycerol (Fisher Chemical, G/0650/17)) was added and incubated at 4 °C with rotation for 2 h. After incubation, the beads were washed three times with 500µl the binding buffer with vortex and centrifugation at 4 °C

for 1 min, 500 g to remove the supernatant. Proteins were finally eluted in 20µl 2xSDS-PAGE sample loading buffer and then analysed by Western Blot.

Lentiviral Line Generation

HeLa cells were transduced with viral supernatants and after three days, cells were subcultured into medium containing puromycin (1.0 µg/ml) to select for transduced cells. Medium containing puromycin was replaced every two to three days and following ten days of selection frozen stocks were made. Cells were maintained in medium containing puromycin, but when performing experiments with the lines, puromycin was removed. Western blotting was used to validate the lentiviral lines.

Immunofluorescence microscopy

For endogenous protein detection, following fixation cells were permeabilised with 0.3% Triton and blocked with 2% BSA/0.025% Tween. Primary antibodies were diluted in 2% BSA/0.025% Tween in PBS as follows: TNRC6A (1:300), AGO1 (1:400 Millipore) AGO2 (Rabbit 1:200, Goat 1:100, Mouse 1:400), AGO3 (1:400 Millipore), AGO4 (1:400 Millipore) LIMD1 (1:300), cNOT1 (1:100), cNOT9 (1:50), DDX6 (1:200), Phospho-AGO2 (1:100). Cells were washed in PBS and stained with Alexa-Fluor conjugated secondary antibodies (1:500 or 1:2000) (Life Technologies). Images were captured at x40 or x100 magnification with a Zeiss LSM 710 confocal microscope.

Immunoprecipitation

For immunoprecipitation of endogenous proteins, Dynabeads® Co-immunoprecipitation Kit including Dynabeads® M-270 Epoxy beads was used. 1.5mg of beads/IP were conjugated with 7.5µg/mg beads of immunoprecipitating antibody overnight at 37ºC with shaking. Cells were collected, centrifuged, and resuspended in P-body lysis buffer (Chu and Rana, 2006) supplemented with 'Complete' protease inhibitors (Roche) and PhosSTOP phosphatase inhibitors (Roche) at a 1:9 ratio of cell pellet weight (mg) to lysis buffer volume (µl). Lysates

were incubated on ice for 15 minutes and centrifuged at 2,600 rpm for 5 minutes. The cleared lysate was rotated at 4ºC for 30 minutes with the antibody-conjugated Dynabeads®, which were subsequently washed 4 times with unsupplemented P-body lysis buffer. Protein complexes were eluted in 2x SDS-PAGE sample buffer or 0.1M glycine pH 2.5 and analysed by western blot.

For immunoprecipitation of ectopically expressed proteins, transfected HEK293T cells were lysed by the addition of ice-cold RIPA buffer (150mM NaCl, 1%(v/v) IGEPAL-630, 0.5%(w/v) sodium deoxycholate, 0.1%(w/v) SDS, 50mM Tris, pH 8) supplemented with 'Complete' protease inhibitors (Roche) and PhosSTOP phosphatase inhibitors (Roche) and scraped using a cell scraper. Lysates were rotated at 4°C for 20min and centrifuged at 14,800 rpm for 10min to pellet debris. Protein concentration of cleared lysate was determined by Bradford assay and 500 µg of protein was used for IP. The cleared lysate was rotated at 4°C for 4h with immunoprecipitation matrix (Santa Cruz sc-45042) previously conjugated to 2µg of immunoprecipitating antibody (2% BSA/PBS, 4ºC for 2 hours). The immunoprecipitation matrix–antibody complex was then washed three times with ice-cold RIPA, and protein complexes were eluted in 5×SDS–PAGE sample buffer and analysed by Western blot.

Protein Mass Spectrometry

Protein bands were excised from the gel, destained, reduced, alkylated and digested with trypsin following standard protocols. The resulting peptide solutions were desalted using U-C18 ZipTips (Millipore) following the manufacturer's instructions. Samples were spotted directly onto the MALDI target plate in addition to matrix solution (α-Cyano-4-hydroxycinnamic acid).

MS/MS analysis was performed on a Bruker Ultraflex in positive ion, reflector mode. MS spectra were acquired in the mass range m/z 800-4000. The 10 most intense peaks in the MS spectra for each sample were then selected for MS/MS analysis. The MS/MS datawere submitted to database searching against NCBInr, using a locally running copy of the Mascot

software (Matrix Science) through a Biotools (Bruker Daltonics) interface. Scores greater than 62 indicate identity or extensive homology ($p<0.05$).

Real-time quantitative PCR

RNA was extracted from cells using TriReagent (Sigma) or Reliaprep RNA miniprep (Promega) according to manufacturers' instructions. Following treatment with DNase I, RNA was diluted and added to GoTaq 1-step RT-qPCR (Promega) reactions along with specific primers. RT-qPCR was performed on the Applied Biosystems 7500 Real-Time PCR System and relative amounts of targets were quantified using the $2^{-\Delta\Delta Ct}$ method.

CRISPR-Cas9 Cell Line Generation

The gRNA sequence (5'-GGTCTTCCAAGATCAAACTC) targets Exon 1 of LIMD1. Transduced HeLa cells were initially selected with puromycin (1 µg/ml) prior to screening of single cell colonies for LIMD1 knockout by Western blot. Genetic mutation was confirmed by Sanger sequencing of genomic DNA utilising the primers 5' GAGTAGAGGCCCTGTCAATGG and 5' CACAGATCCCAGGCTACCATC.
